# Design, Conduct, Analysis, and Reporting of Therapeutic Efficacy Studies in Visceral Leishmaniasis: A Systematic Review of Published Reports, 2000–2021

**DOI:** 10.4269/ajtmh.23-0458

**Published:** 2024-07-02

**Authors:** Prabin Dahal, Sauman Singh-Phulgenda, Caitlin Naylor, Matthew Brack, Mitali Chatterjee, Fabiana Alves, Philippe J. Guerin, Kasia Stepniewska

**Affiliations:** ^1^ Infectious Diseases Data Observatory, Oxford, United Kingdom;; ^2^ Centre for Tropical Medicine and Global Health, Nuffield Department of Medicine, University of Oxford, Oxford, United Kingdom;; ^3^ Institute of Postgraduate Medical Education & Research, Kolkata, India;; ^4^ Drugs for Neglected Diseases initiative, Geneva, Switzerland

## Abstract

A systematic review (SR) of published efficacy studies in visceral leishmaniasis (VL) was undertaken to describe methodological aspects of design, conduct, analysis, and reporting. Studies published during 2000–2021 and indexed in the Infectious Diseases Data Observatory VL library of clinical studies were eligible for inclusion (*N* = 89 studies). Of the 89 studies, 40 (44.9%) were randomized, 33 (37.1%) were single-armed, 14 (15.7%) were nonrandomized multiarmed studies, and randomization status was unclear in two (2.2%). After initial screening, disease confirmation was done by microscopy in 26 (29.2%) and by a combination of serology and microscopy in 63 (70.8%). Post-treatment follow-up duration was <6 months in three (3.3%) studies, 6 months in 75 (84.3%), and >6 months in 11 (12.4%) studies. Confirmation of relapse was solely based on clinical suspicion in four (4.5%) studies, parasitological demonstration in 64 (71.9%), using molecular/serological/parasitological method in 6 (6.7%), and there was no information in 15 (16.9%). Of the 40 randomized studies, sample size calculation was reported in only 22 (55.0%) studies. This review highlights substantial variations in definitions adopted for disease diagnosis and therapeutic outcomes suggesting a need for a harmonized trials protocol.

## INTRODUCTION

Visceral leishmaniasis (VL) is the most severe of the three forms of leishmaniasis and is almost always fatal without treatment.^1^ Antileishmanial drugs substantially reduce mortality, and their safety and efficacy have been evaluated in several randomized and nonrandomized studies.^2^ Synthesis of evidence from published VL literature have usually focused on description of the spectrum of the patient population enrolled, the heterogeneity in treatment regimens, and the characterization of drug efficacy and safety.^2^^–^^4^ Assessment of variability in methodological aspects of the studies included is a crucial component in gauging the robustness of findings from such evidence synthesis efforts.^5^^,^^6^ However, the scale and impact of such methodological variation remains largely underappreciated in the context of VL efficacy studies. This systematic review aimed to present the landscape of the design, conduct, analysis, and reporting of published efficacy studies in VL since 2000 (2000–2021).

## MATERIALS AND METHODS

### Information sources and search strategy.

This review synthesizes data from studies indexed in the Infectious Diseases Data Observatory (IDDO) VL living systematic review (VL LSR) library of clinical studies.^7^ The IDDO VL LSR indexes prospective clinical efficacy studies on antileishmanial therapies published since 1980; a detailed description on the databases searched and the search strategy adopted is presented elsewhere.^8^ Briefly, the VL LSR is updated on a periodic basis and searches the following databases: Ovid Embase, Scopus, Web of Science Core Collection, Cochrane Central Register of Controlled Trials, clinicaltrials.gov, WHO ICTRP, as well as IMEMR, IMSEAR, and LILACS from the WHO Global Index Medicus.^8^

### Study selection and data extraction.

For the purpose of this review, studies published after 2000 (inclusive) were considered. Data on the following aspects were extracted: inclusion and exclusion criteria adopted, case definition, sample source (tissue aspirate) used for VL confirmation, details of randomization procedures, blinding status, follow-up duration, the number of participants enrolled, clinical endpoints adopted and their definition, details of the laboratory procedures adopted, methodological details on statistical approaches such as sample size estimation, assessment of lost-to-follow-up, and description of the analytical approaches used for deriving efficacy estimate.

### Data summary.

Descriptive summaries were presented for the characteristics of the studies included in the review. No patient-related outcome data were analyzed, and hence risk of bias assessment in studies included was not carried out.

## RESULTS

The IDDO VL LSR has indexed 89 studies published from January 1, 2000 through November 17, 2021.^7^ The library included 61 (68.5%) studies from the Indian subcontinent, 16 (18.0%) from East Africa, four (4.5%) from the Mediterranean region, four (4.5%) from South America, three (3.4%) from Central Asia (the Middle East), and one (1.1%) was a multiregional study. Of the 89 studies, 28 (31.5%) studies were published during 2000–2004, 20 (22.5%) during 2005–2009, 23 (25.8%) during 2010–2014, and 18 (20.2%) were published in or after 2015. There were 27,070 patients enrolled in 187 drug arms with a median sample size of 51 (range: 1–3,126) patients per arm. Further description of the studies included is presented in Supplemental Table 1.

### Study design and conduct.

Of the included studies, 40 (44.9%) were randomized, 33 (37.1%) were single-armed, 14 (15.7%) were nonrandomized multiarmed studies, and randomization status was unclear in two (2.2%). The blinding status of the studies were as follows: 59 (66.3%) were open-label studies, two (2.2%) were blinded, and blinding status was not stated in 28 (31.5%) studies. The median sample size per study was 152 (interquartile range [IQR]: 84–400; range: 25–1,485] for randomized studies and 120 (IQR: 60–309; range: 12–3,126) for nonrandomized studies.

### Randomized studies (*N* = 40 studies).

**Figure 1.**
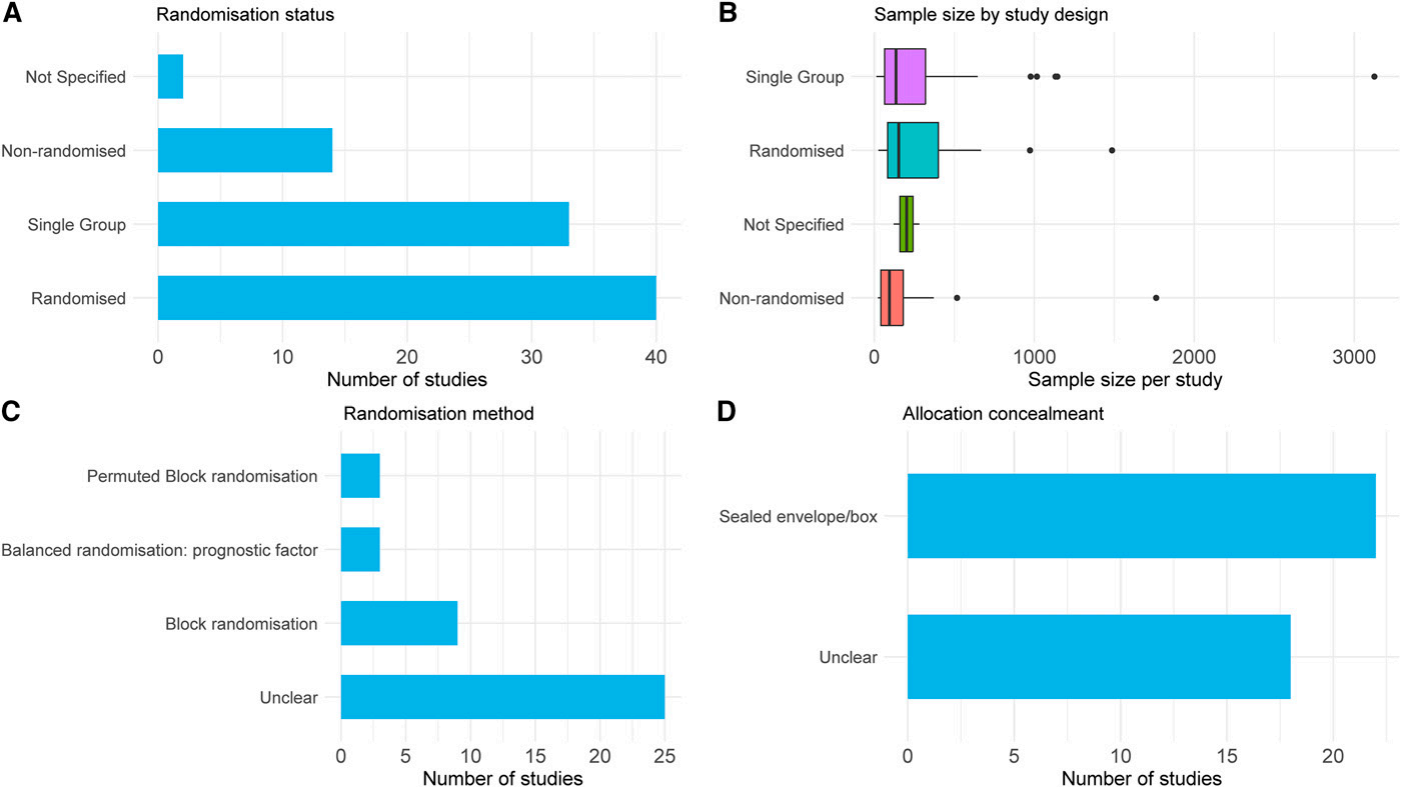
Design aspects of studies included in the review.

Block randomization was used in 12 studies (three used permuted block randomization) with the block sizes ranging from 4 to 28 (Figure 1). Randomization was carried out by balancing the treatment regimens on at least one prognostic factor in three studies, whereas such details were missing in 25 studies (Supplemental Table 2). Sequence generation was carried out using a computerized system in 22 (55.0%) studies, using a random number table in two (5.0%) studies, and was unclear in 16 (40.0%) studies (Figure 1). Allocation was concealed using a sealed, opaque envelope/box in 22 (55.0%) studies (Figure 1). Of the 40 randomized studies, two were blinded, 33 were open labeled, and blinding status was not stated in five studies.

### Inclusion and exclusion criteria adopted.

A complete list of inclusion and exclusion criteria adopted for patient enrollment is presented in Supplemental Table 3.

### Informed consent.

Requirement of informed consent (or assent for children) was stated as a part of inclusion/exclusion criteria in 21 (23.6%) studies, it was not explicitly stated as a part of the inclusion/exclusion criteria but was collected before patient enrollment in 57 (64.0%) studies, and there was no statement regarding consent in 11 (12.4%) studies (Table 1).

**Table 1:** Inclusion/exclusion criteria reported

Criteria	Number of Studies (*N* = 89)	%
Patient demographics		
Eligible age range		
Less than 15 years	12	13.5
Adults	6	6.7
All ages	53	59.6
Unclear	18	20.2
Female patients		
Included	86	96.6
Excluded	1	1.1
Unclear	2	2.2
Pregnancy and lactation		
Excluded	54	60.7
Included	6	6.7
Unclear	29	32.6
Inclusion of women susceptible to becoming pregnant		
Included	26	29.2
Inclusion conditional on pregnancy test and/or contraception usage	15	16.8
Excluded	3	3.4
Unclear	45	50.6
Disease and treatment history		
Type of infection		
Primary	49	55.1
Secondary	7	7.9
Mixture	15	16.9
Unclear	18	20.2
Patients with severe/critical VL		
Excluded	15	16.9
Included	8	8.9
Unclear	66	74.2
Previously treated patients		
Excluded	46	51.7
Included	21	23.6
Unclear	22	24.7
Time from last exposure to VL drugs		
<2 months (10–60 days) (or 5 half-lives)	11	12.4
2–6 months	12	13.5
>6–12 months	3	3.4
Unclear	63	70.8
History of allergy/hypersensitivity to antileishmanial drugs		
Excluded	34	38.2
Unclear	55	61.8
Biological range adopted		
At least one was part of study exclusion		
Yes	49	55.1
Unclear	40	44.9
Minimum hemoglobin concentration		
3 g/dL	1	1.1
>3 to 5 g/dL	29	32.6
>5 to 7 g/dL	12	13.5
Unclear	47	52.8
Prothrombin time (above control values), seconds		
>4	3	3.4
>5	13	14.6
>15	2	2.2
Prothrombin activity <40%	1	1.1
INR >2	1	1.1
Unclear	69	77.5
Minimum platelets concentration		
>40,000/*µ*L	22	24.7
>50,000/*µ*L	14	15.7
Combination of ranges	3	3.3
Unclear	50	56.2
Other characteristics		
Informed consent/assent		
Required	21	23.6
Not a part of I/E criteria but collected before enrollment	57	64.0
Unclear (not mentioned)	11	12.4
Inability to follow study protocol*		
Excluded	19	21.3
Unclear	70	78.7

I/E = inclusion/exclusion criteria; INR = International normalization ratio; VL = visceral leishmaniasis. Percentages may add to 99.9% or 100.1% due to rounding.

* Includes inability to come for return visit or attend scheduled visits, living far away from the study site, or inability to comply with medication.

### Eligible age range and included age range.

Eligible age range for inclusion were children <15 years in 12 (13.5%) studies, adults (≥18 years) in six (6.7%), patients of all ages were eligible in 53 (59.6%), and age range was not defined as a part of inclusion/exclusion criteria in 18 (20.2%) studies (Table 1). The age range of included patients were children <15 years in 13 (14.6%), adults in seven (7.9%), and patients of all ages in 69 (77.5%) studies. Infants were included in five (5.6%) studies, excluded in 72 (80.9%), and their inclusion was unclear in the remaining 12 (13.5%) studies. The maximum of eligible/included age was 65 years in 16 (18.0%) studies, the upper range was 66 to 80 years in 10 (11.2%) studies, and the age-range was unclear in 18 (20.2%) studies (Supplemental Table 1).

### Enrollment of female, pregnant women, and women susceptible to becoming pregnant.

Females were excluded in one (1.1%) study, included in 86 (96.6%) studies, and the inclusion/exclusion status was unclear in two (2.2%) studies. Pregnant and lactating women were excluded in 54 (60.7%) studies, included in six (6.7%), and their inclusion/exclusion could not be discerned in 29 (32.6%) studies (Table 1 and Supplemental Table 3). Women of childbearing age (or those who had reached menarche) were excluded in three (3.4%) studies, their inclusion was conditional on negative pregnancy test or willingness to undertake contraception in 15 (16.9%) studies, were included without description of pregnancy test/contraception use requirements in 26 (29.2%) studies, and the information was unclear in 45 (50.6%) studies.

### Comorbidities.

**Figure 2.**
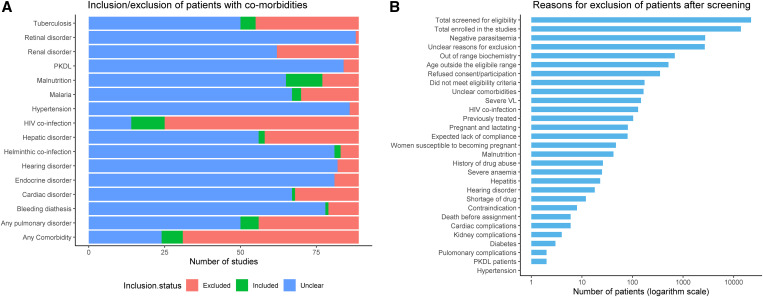
Eligibility criteria and reasons for patient exclusion. (**A**) Endocrine disorders included patients with diabetes and pancreatitis. Hepatic disorders including jaundice, hepatitis, or hepatic encephalopathy. Any pulmonary condition included pneumonia, tuberculosis, or any respiratory illness. Bleeding diathesis including coagulation disorders, G6PD deficiency, or any hematological disorders. (**B**) Presents data from 46 studies that clearly reported the patient flow. Sample size by study design on logarithm (base10) scale is shown. PKDL = post–kala-azar dermal leishmaniasis; VL = visceral leishmaniasis.

Patients with HIV; tuberculosis (TB); and hepatic, renal, and cardiac disorders were the most common comorbidities as a part of the exclusion criteria adopted (Figure 2). Patients with at least one comorbidity were clearly excluded in 58 (65.2%) studies, included in seven (7.9%) studies, and this could not be discerned in 24 (27.0%) studies. In particular, patients coinfected with HIV were excluded in 64 (71.9%) studies, included in 11 (12.4%) studies, and their inclusion status was unclear in 14 (15.7%) studies. Those with hepatic disorders were excluded in 31 (34.8%) and included in two (2.2%) studies (unclear in 56 studies). Patients with TB coinfection were excluded in 34 (38.2%) studies and included in five (5.6%) (unclear in 50 studies). Patients with renal disorders were excluded in 27 (30.3%) studies, and those with cardiac disorders were excluded in 21 (23.6%) studies. Other comorbidities reported that formed the part of the exclusion criteria included malnutrition, helminth coinfections, malaria, endocrine disorders such as diabetes and pancreatitis, hypertension, post–kala-azar dermal leishmaniasis, and hearing and bleeding disorders (Figure 2).

### Nutritional status.

Details of nutritional status was extracted from either eligibility criteria stated in the study protocol or from the description of patient characteristics reported in the study. Overall, malnourished patients were included in 13 (14.6%) studies, excluded in eight (9.0%), and the information was unclear in 68 (76.4%) studies. Of the 21 studies that clearly indicated inclusion/exclusion of malnourished patients, assessment of malnutrition was based on anthropometric indicators in 14 studies, clinical definition was used in one study, and the criteria used for assessment of malnutrition was unclear in six studies (Supplemental Table 4).

### Treatment history and disease severity.

Patients with a history of prior antileishmanial treatment within a predefined period (time range: 10 days–12 months) were excluded in 46 (51.7%) studies, included in 21 (23.6%) studies, and this was unclear in 22 (24.7%) studies. Those with a history of hypersensitivity/allergy to the study medications were excluded in 34 (38.2%) studies (Table 1). Patients who were described as cases of severe VL or disease with critical illness were excluded in 15 (16.9%) studies, included in eight (8.9%), and this was unclear in 66 (74.2%) studies (Table 1 and Supplemental Table 5).

### Parasite-related criteria.

Patients with freshly diagnosed VL (primary infection) were enrolled in 49 (55.1%) studies, patients with secondary cases (including resistant, previously unresponsive, or relapsing cases) were enrolled in seven (7.9%) studies, a mixture of primary and secondary cases were included in 15 (16.9%), and this was unclear in 18 (20.2%) studies (Table 1).

### Hematological measures.

*Hemoglobin*. The minimum hemoglobin concentration required for inclusion was 3 g/dL in one (1.1%) study, between >3 and 5 g/dL in 29 (32.6%) studies, >5 and 7 g/dL in 12 (13.5%) studies, and was unclear in 47 (52.8%) studies (Table 1).

*Platelets*. The exclusion threshold for platelet counts was <40,000/*µ*L in 22 (24.7%) studies, <50,000/*µ*L in 14 (15.7%), a range of other thresholds were used in three (3.4%) studies, and the eligible range was not reported in 50 (56.2%) studies (Table 1).

*White blood cells (WBC)*. An exclusion threshold of granulocytes <1,000/*µ*L was adopted in 13 (14.6%) studies, WBC counts <750/*µ*L was adopted in one (1.1%) study, WBC counts <1,000/*µ*L in 18 (20.2%) studies, WBC <2,000/*µ*L in four (4.5%) studies, and WBC threshold was not specified in eligibility criteria in 53 (59.6%) studies (Supplemental Table 3).

### Liver enzymes and liver function.

*Albumin*. Serum albumin concentration <2.0 g/dL was an exclusion criterion in two (2.2%) studies, those with >3 times the upper limit of normal (ULN) were excluded in three (3.4%) studies, those with significant proteinuria were excluded in one (1.1%) study, and albumin concentration was not part of eligibility criteria in 83 (93.3%) studies (Supplemental Table 3).

*Bilirubin*. The exclusion range for bilirubin concentration was >3 times the ULN in nine (10.1%) studies, >2 ULN in seven (7.9%), >1.5 ULN in two (2.2%), >ULN in one (1.1%), >2 normal range in three (3.4%), >5 normal range in 1 (1.1%) study, bilirubin concentration higher than 34.2 to 221 *µ*mol/L were used in six (6.7%) studies, and bilirubin was not stated as eligibility criteria in 60 (67.4%) studies (Supplemental Table 3).

*Aminotransferases*. The exclusion ranges for aminotransferase (aspartate aminotransferase/alanine aminotransferase) measurements were >2.5 ULN in three (3.4%) studies, >3 ULN in 23 (25.8%) studies, >4 ULN in two (2.2%) studies, >5 ULN in three (3.4%), >10 ULN in one (1.1%), >3 times the normal range in three (3.4%) studies, >5 times the normal range in one (1.1%), >200 IU in one (1.1%), and aminotransferase levels were not part of eligibility criteria in 52 (58.4%) studies (Supplemental Table 3).

*Prothrombin time (PT)*. PT was clearly reported as a part of exclusion criteria in 20 (22.5%) studies: PT >4 seconds above control was required in three studies, >5 seconds was required in 13 studies, PT >15 seconds in two studies, and international normalized ratio >2 was required in two studies. Further breakdown in presented in Table 1.

### Renal function.

*Serum creatinine*. The exclusion ranges adopted for creatinine concentration were as follows: >1.5 mg/dL in one (1.1%) study, >2.0 mg/dL in seven (7.9%), outside or above the normal range (without further details) in 10 (11.2%) studies, >1.5 ULN/normal range in 14 (15.7%) studies, >2 ULN in three (3.4%), >3 ULN in one (1.1%), and >1.5 normal limit in three (3.4%). Creatinine was not part of the eligibility criteria in 50 (56.2%) studies (Supplemental Table 3).

*Blood urea nitrogen (BUN)*. The exclusion ranges adopted for BUN were >1.5 ULN/normal range in 11 (12.4%) studies, and BUN measurements were not part of eligibility criteria in 78 (87.6%) studies.

*Urine urea concentration*. Patients with urine urea concentration >2 ULN were excluded in two (2.2%) studies, and this was not mentioned in 87 (97.8%) studies.

### Other characteristics.

Ability to comply with the scheduled follow-up or proximity to the study center was stated as an essential criterion for inclusion in 19 (21.3%) studies. Other occasionally adopted exclusion criteria were use of prohibited/contraindicated drugs (seven studies), alcohol/drug abuse (five studies), life expectancy of <6 months (two studies), undergoing major surgical procedure (two studies), contraindication for splenic/bone marrow aspirate (three studies), and abnormal potassium concentration (*n* = 1 study) (Supplemental Table 3).

## PATIENT SCREENING

### Case definition.

Case definition adopted for patient screening was presented in 77 (86.5%) studies, with no information available in 12 (13.5%) studies. Overall, 17 signs and symptoms were part of the case definition (Table 2). A combination of fever and splenomegaly/hepatomegaly was adopted in 24 (27.0%) studies; a combination of fever and hepatosplenomegaly and weight loss/loss of appetite in 14 (15.7%) studies; a combination of fever, splenomegaly, and a hematological measure (cytopenia/anemia/thrombocytopenia) in six (6.7%) studies; case definition was unclear in 30 (33.7%) studies; and a combination of clinical factors was used in the remaining 15 (16.9%) studies (Table 2 and Supplemental Table 6).

**Table 2:** Case definition used for patient screening and confirmation of disease status

Case Definition and Confirmation Method	Number of Studies (*N* = 89)	%
Case definition for patient screening		
Compatible clinical diagnosis* (fever, splenomegaly etc.)	77	86.5
Not specified (no defined criteria stated)	12	13.5
Definition of compatible clinical diagnosis		
Fever or splenomegaly or cytopenia	3	3.4
Fever + splenomegaly	18	20.2
Fever + hepatomegaly/splenomegaly	3	3.4
Fever + splenomegaly/wasting + weight loss/loss of appetite	19	21.3
Fever + splenomegaly + hematological measurement	6	6.6
Fever + splenomegaly + chills + rigor	3	3.4
Fever + splenomegaly/wasting + weight loss/loss of appetite + hematological measurement	7	7.9
Not specified	30	33.7
Length of fever used in case definition for patient screening		
>1 week	1	1.1
≥2 weeks	19	21.3
Not specified	69	77.6
Case confirmation method		
Parasitological	26	29.2
Serological and/or parasitological	56	62.9
Serological + parasitological	7	7.9

Percentages may add to 99.9% or 100.1% due to rounding.

* The following were part of the definition of suspected cases of visceral leishmaniasis: fever, splenomegaly, hepatomegaly, hepatosplenomegaly, chills, rigor, weight loss, loss of appetite, wasting, epistaxis, anemia, weakness, asthenia, cytopenia, leukopenia, thrombocytopenia, or lymphadenopathy.

### Disease confirmation and reasons for patient exclusion.

Among patients meeting the case definition of VL, disease confirmation required parasitological demonstration using a tissue aspirate in 26 (29.2%) studies, and a combination of serological and/or parasitological method was adopted in 63 (70.8%) studies.

Of the 89 studies included, 46 (51.7%) clearly presented a patient flow diagram (or CONSORT [Consolidated Standards of Reporting Trials] checklist) (Supplemental Table 7). Overall, 22,056 patients were screened in these 46 studies, and a total of 13,878 (62.9%) patients were enrolled. Of the latter, 8,178 patients were excluded. The documented reasons for exclusion were as follows: 2,723 (33.3%) were deemed not to have VL upon further parasitological/serological examination (2,454 had negative bone-marrow/splenic aspirate and 269 had negative serology), 687 (8.4%) had biochemistry/biological measurements outside of permissible range, 515 (6.3%) of the patients were outside the inclusion age range, and 350 (4.3%) patients refused to participate or give consent (Figure 2). Further diagnostic details of these studies are presented in Supplemental Table 8.

## DETAILS OF THE PARASITOLOGICAL AND SEROLOGICAL PROCEDURES

### Parasite speciation and *Leishmania* zymodeme (isoenzyme) characterization.

*Leishmania donovani* (LD) was the stated causative parasite in 23 (25.8%) studies, *L. infantum/L. chagasi* in three (3.4%) studies, and the parasites species (genus *Leishmania*) was not stated in 63 studies (70.8%) (VL is caused by *L. donovani* in East Africa and the Indian subcontinent and by *L. infantum* in the Mediterranean region and South America^9^) (Table 3). Isoenzyme characterization of the parasite strains was explicitly carried out in two studies; one was from the Mediterranean region,^10^ and the other was from East Africa.^11^

**Table 3:** Details of the laboratory procedures adopted

Parasite and Laboratory Variables	Number of Studies (*N* = 89)	%
Parasite species		
*Leishmania donovani*	23	25.8
*Leishmania infantum*/*Leishmania chagasi*	3	3.4
Not explicitly reported	63	70.8
Staining method used in studies using parasitology		
Giemsa	23	25.8
Giemsa and/or Leishman stain or Diff-Quik stain	2	2.2
Unclear	58	65.2
Tissue aspirate not used	6	6.7
Details of serological tests used		
rk39	19	21.3
DAT	5	5.6
IFA/IFAT/ELISA	4	4.5
Combination of rk39 with DAT/IFA	4	4.5
Serology not used	57	64.0
Blinding of laboratory procedures for parasitology		
Yes	21	23.6
Not blinded	2	2.2
Unclear	66	74.2
Quality control of laboratory procedures for parasitology		
Slides read by two readers or independent microscopist	4	4.5
Slides read by a single microscopist/single laboratory	1	1.1
Slides read by trained/experienced technician	2	2.2
All/sample of the slides were reread by an external or the same microscopist	3	3.4
Two slides per sample read	1	1.1
Not specified	78	87.6
Was parasite gradation carried out?		
Graded	49	55.1
Not graded	3	3.4
No information	37	41.6
Was promastigotes culture carried out?		
Yes*	11	12.4
No information	78	87.6
Was parasite strain diversity (zymodeme) characterized?		
Yes	2	2.2
No	87	97.8
Was the presence of amastigotes demonstrated?		
Yes	15	16.9
Not explicitly stated	74	83.1

DAT = direct agglutination test; IFA = immunofluorescence assay; IFAT = immunofluorescence antibody test; QC = quality control; rk39 = recombinant K39 antigen (rk39) based rapid diagnostic test. Percentages may add to 99.9% or 100.1% due to rounding.

* Cultures using biphasic medium: Novy–MacNeal–Nicolle medium.

### Parasite staging (smear and culture).

Parasitological demonstration was based on the identification of amastigotes form of the parasites using a tissue aspirate in 15 (16.9%) studies, and this was not explicitly stated in 74 (83.1%) studies. In addition, promastigotes culture (biphasic medium: Novy–MacNeal–Nicolle medium) was carried out in 11 (12.4%) studies (Table 3).

### Tissue aspiration.

**Figure 3.**
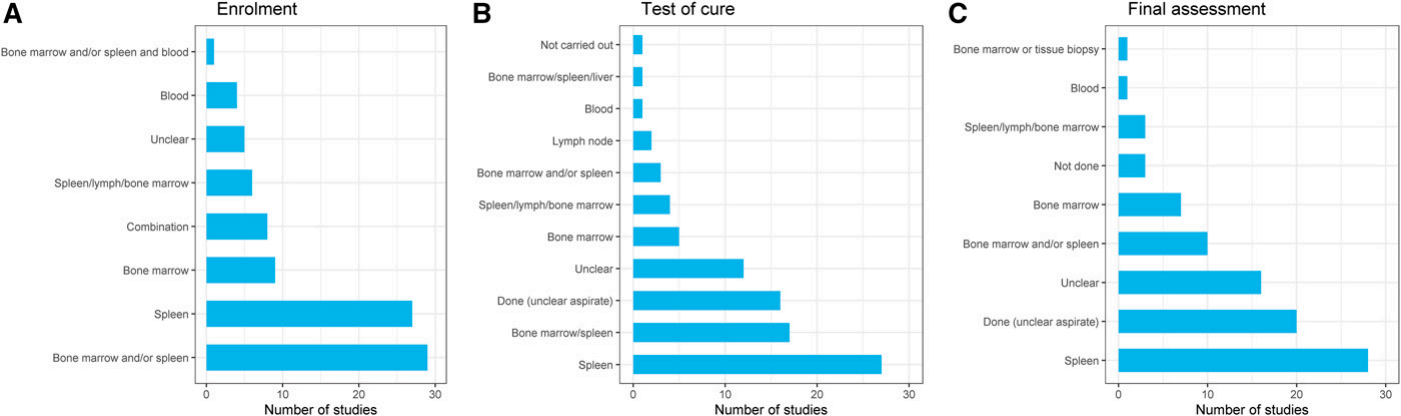
Tissue aspiration used. Supplemental File 1 provides the details for each of the studies included separately.

Overall, 83 studies used parasitological (microscopy) method (either solely or in combination with serological, culture or molecular method) for confirming the presence of parasites, and six studies used only serological tests. Of the 83 studies that used parasitological method, Giemsa stain was used in 23, Giemsa or Leishman stain or Diff-Quik stain was used in two studies, and the staining method was not stated in 58 studies (Table 3). Splenic tissue aspiration was used in 27 (30.3%) studies; a combination of bone marrow, spleen, or lymph node aspirate was used in 53 (59.6%) studies; blood sample was used in four (4.5%); and the tissue used was unclear in five (5.6%) (Figure 3). Further details are presented in Supplemental Table 9.

### Parasite quantitation.

Parasite density was graded in 49 (55.1%) studies, gradation was not done in three (3.4%) studies, and the information was not reported in 37 (41.6%) studies (Table 3). Parasite gradation used microscopy in 38 (42.7%) studies (using a semi-quantitative scale), polymerase chain reaction (PCR) in two (2.2%) studies, gradation was not done in three (3.4%) studies, and the information was unclear in 46 (51.7%) studies. Of the two studies that adopted PCR, one study quantified parasite density using peripheral blood,^12^ and the other used both semi-quantitative microscopy counts on tissue aspirate and parasite load in peripheral blood.^11^

### Details of serological method.

A total of 32 studies used serological methods for VL confirmation (in combination with other methods or solely). Of the 32 studies, recombinant K39 antigen (rk39) based rapid diagnostic test was used in 19, direct agglutination test (DAT) was used in five, immunofluorescence assay (IFA/immunofluorescence antibody test or ELISA) in four and a combination of rk39 with DAT or IFA in the remaining four studies (Table 3).

### Quality control of laboratory procedures.

In 21 (23.6%) studies, the laboratory procedures were blinded to the treatment regimen, two (2.2%) studies did not blind the laboratory procedures, and this information was not reported in 66 (74.2%) studies.

Only 11 (12.4%) studies reported carrying out quality control (QC) of laboratory procedures, and this information was not reported in 78 (87.6%) studies. Of the 11 studies that reported QC, slides were read by two readers/independent microscopist in four studies, all or a random sample of the slides were reread by an external or the same microscopist in three studies, two slides were read per sample in one study, the slides were read by a single reader in one study, and slides were read by a trained/experienced technician in two studies (Table 3).

## PATIENT OUTCOME ASSESSMENTS

### Initial assessment of test of cure and tissue aspiration.

Initial assessment upon completion of the treatment regimen was carried out solely based on clinical signs and symptoms in 13 (14.6%) studies, parasitological assessment (with or without clinical assessment) was carried out in 71 (79.8%) studies, and the details were not reported in five (5.6%) studies. Test of cure evaluation (clinical cure, parasitological cure, or clinical and parasitological cure) was carried out within 14 days of treatment completion in three (3.4%) studies, between 15 to 30 days in 68 (76.4%) studies, between 31 to 70 days in seven (7.9%) studies, a mixture of different time-points was adopted in two (2.2%) studies, and the time of assessment was unclear in nine (10.1%) (Table 4).

**Table 4:** Variability in outcome assessments

Treatment Outcomes and Definitions Adopted	Number of Studies (*N* = 89)	%
Primary endpoint adopted		
Initial cure or negative parasitemia at the end of therapy	12	13.5
Ultimate cure/definitive cure/final cure at end of ≥6 months	27	30.3
Pharmacokinetics measurements at day 21	1	1.1
Safety outcomes	5	5.6
Not explicitly stated	44	49.4
Assessment at end of the treatment (test of cure assessment)*		
Clinical	13	14.6
Clinical + parasitological	69	77.5
Clinical and/or parasitological	2	2.2
No information	5	5.6
Time point of test-of-cure assessment		
<15 days	3	3.4
15–30 days	68	76.4
31–70 days	7	7.9
One or more of the above	2	2.2
Not stated	9	10.1
Assessment at end of the study follow-up (for final outcome)^†^		
Clinical assessment	14	15.7
Clinical and parasitological^†^	65	73.0
Clinical and/or parasitological^†^	2	2.2
Clinical and parasitological and molecular	1	1.1
Clinical and parasitological/serological	1	1.1
Not stated	6	6.7
Time point of end of the study assessment		
<6 months	3	3.4
6 months	75	84.3
>6 to <12 months	2	2.2
12 months	8	9.0
>12 months	1	1.1
Definition of relapse		
Based on clinical suspicion	4	4.5
Clinical suspicion and/or parasitological demonstration	2	2.2
Parasitological demonstration	64	71.9
Parasitological or serological	1	1.1
Molecular and/or parasitological	3	3.4
Not explicitly stated	15	16.9

Percentages may add to 99.9% or 100.1% due to rounding.

* Achievement of clinical and parasitological resolution at the end of the treatment was usually defined as “initial cure.” Equivalent terminology adopted were apparent cure, clinical and parasitological cure, apparent response or cure.

^†^ Achievement of clinical and parasitological cure at the end of the study follow-up was usually defined as “definitive cure.” Equivalent terminology adopted were: full cure, final cure, definite cure, ultimate cure, complete cure or cure.

Tissue aspiration used at test of cure included splenic sample in 27 (30.3%) studies, bone marrow in five (5.6%), lymph node in two (2.2%), a combination of bone marrow/spleen/lymph was used in 25 (28.1%) studies, the aspirate used was unclear in 16 (18.0%) studies, information was not reported in 12 (13.5%), and aspiration was not done in one (1.1%) study (Figure 3 and Supplemental Table 10).

### Duration of post-treatment follow-up.

Post-treatment follow-up duration was <6 months in three (3.3%) studies, 6 months in 75 (84.3%) studies, 7 months in one (1.1%) study, 9 months in one (1.1%), 12 months in eight (9.0%), and 2.8 years in one (1.1%) study (Table 4).

### Assessment of relapse and tissue aspiration.

Relapse was defined solely based on clinical suspicion in four (4.5%) studies, parasitological demonstration upon clinical suspicion in 64 (71.9%), based on molecular method (solely or in combination with parasitological method) in three (3.4%) studies, using clinical and/or parasitological assessments in two (2.2%), using parasitological or serological assessment in one (1.1%) study, and the definition of relapse was unclear in 15 (16.9%) studies.

Tissue aspiration used for confirmation of relapse included splenic aspiration in 28 (31.5%) studies, bone marrow in seven (7.9%) studies, bone marrow and/or spleen/lymph in 14 (15.7%) studies, peripheral blood in one (1.1%) study, tissue aspiration was done but the sample used was unclear in 30 (33.7%), aspiration was not done in three (3.4%) studies, and aspiration status was unclear in six (6.7%) studies (Figure 3). Further details are presented in Supplemental Table 10.

## STATISTICAL ANALYSES

### Sample-size calculation.

Sample-size calculation was carried out in 34 (38.2%) studies, not done in 10 (11.2%), and no information was presented in 45 (50.6%) studies (Table 5). Of the 40 randomized studies, sample size calculation was reported in only 22 (55.0%) studies.

**Table 5:** Statistical considerations

Analytical Considerations	Number of Studies (*N* = 89)	%
Objective of sample size estimation		
To detect difference in cure rate (difference: 8%–40%)	13	14.6
Demonstration of noninferiority (5%–20% noninferiority margin)	10	11.2
To estimate cure rate with precision (or 95% CI)	4	4.5
To detect desired efficacy/safety	4	4.5
To detect pharmacokinetics exposure	1	1.1
Unclear objective	2	2.2
Sample size calculation not carried out	10	11.2
No information presented regarding sample size estimation	45	50.6
Anticipated LFU during sample size calculation		
Sample-size adjusted for possible LFU (adjustment: 5%–20%)	10	11.2
Not adjusted (assumed no LFU)	2	2.2
Sample size calculation carried out but no information on LFU adjustment	22	24.7
Sample size calculation not carried out	10	11.2
No information on sample size estimation	45	50.6
Analysis approach for deriving drug efficacy		
Only PP explicitly reported	1	1.1
Only ITT explicitly reported	15	16.9
A modified ITT approach explicitly reported	1	1.1
Both ITT and PP analysis reported	22	24.7
ITT and complete case analysis reported	1	1.1
ITT and evaluation population analysis reported	2	2.2
ITT and on-treatment analysis reported	2	2.2
No explicit distinction/description of ITT or PP analysis	45	50.6
Was an uncertainty interval (95% CI) presented along with the point estimate of cured proportion/efficacy estimate?		
Point estimate presented with a 95% CI	50	56.2
Only point estimate presented	39	43.8
Was survival analysis used?		
Yes: Kaplan–Meier approach to estimate relapse	3	3.4
Yes: Kaplan–Meier approach to fever clearance	2	2.2
Not used	84	94.4

ITT = intention to treat; LFU = lost to follow-up; PP = per protocol analysis. Percentages may add to 99.9% or 100.1% due to rounding.

Of the 34 studies that reported undertaking sample size estimation, two studies reported estimation based on safety endpoint, one based on pharmacokinetics endpoint, one based on a combination of safety and efficacy endpoint, 29 based on efficacy (or cure rate) endpoint, and the endpoint was unclear in one study. In studies that presented the details of sample size estimation, 13 studies aimed to detect a mean difference in cured proportion (effect size: 8%–40%), and 10 studies aimed to demonstrate noninferiority (margin adopted: 5%–20%); further details are presented in Table 5. Sample size adjustment for potential lost-to-follow up was explicitly reported in 10 studies (adjustment range: 5%–20%), and no adjustment was made in two studies (Table 5). Further details are presented in Supplemental Table 11.

### Estimation of drug efficacy.

Cured proportion at the end of the study was presented in all 89 studies included. Point estimate of the efficacy was presented along with an uncertainty interval (95% CI) in only 50 (56.2%) studies. The Kaplan–Meier method was used in five (5.6%) studies; three studies used this method for estimating incidence of relapse and two studies for describing fever clearance (Table 5). In 15 (16.9%) studies, efficacy estimate was reported using an intention-to-treat (ITT) principle, only a per-protocol (PP) approach was reported in one (1.1%) study, a modified ITT was reported in one (1.1%) study, and both ITT and PP analyses were undertaken in 22 (24.7%) studies. In five (5.6%) studies, ITT analysis was reported along with analysis of data from complete cases/evaluable population/on-treatment population. In 45 (50.6%) studies, there was no explicit description of the ITT or PP analysis (Table 5). Further details are presented in Supplemental Table 12.

## DISCUSSION

Our review has characterized the variability in design, conduct, analysis, and reporting of prospective therapeutic efficacy studies conducted in VL for the past 20 years. Several guidelines and checklists (such as CONSORT) have been proposed and developed during this period to facilitate robust reporting of trial design and conduct. Despite this, of the 89 studies included in the review, patient flow diagram was presented in only 46 studies. Systematic evaluation of the patient flow from these studies indicated that one-third of those who were screened for eligibility were excluded. A major reason for such exclusion was testing negative for VL upon further assessment, predominantly using parasitological assessment of bone marrow/splenic aspirate. In general, the clinical manifestations of VL are nonspecific (fever, anemia, and splenomegaly) and are also commonly seen in other infectious diseases, leading to the diagnostic challanges.^13^ However, identification of large proportion of patients who fulfilled the case definition but were eventually negative for VL upon further tissue examination warrants further investigation. Details on the quality control of the microscopy procedures were largely missing. Hence, it is difficult to gauge whether the finding of a large proportion of patients meeting case definition as negative for VL reflects a need for a more sensitive case definition, the limitations of the diagnostic tools, or simply reflects a finding only in a subset of studies with complete reporting (only 46 or 89 studies presented patient flow).

Splenic aspirate was the most commonly used tissue sample for disease confirmation at enrollment. Approximately one-third of the studies used splenic sample for patient enrollment. However, undertaking splenic aspiration is not always feasible in peripheral centers because this procedure requires considerable technical expertise and leads to life-threatening hemorrhages in approximately 0.1% of the procedures and therefore requires blood transfusion provisions and nursing surveillance to be in place.^9^^,^^14^ In patients with low-grade parasitemia, nonpalpable spleen can lead to further difficulty in splenic aspiration. Overall, two-thirds of the studies used bone marrow aspirates. However, the sensitivity of the tissue aspiration is much lower for bone marrow (60%–85%) than for spleen (>95%).^15^ A reliable diagnosis of VL can be challenging, and this urgently necessitates the development of a highly sensitive, noninvasive tools such as molecular methods.^12^ The specificity of immunological tests such as DAT and rK39 remains suboptimal, and approximately 10% to 20% of the patients without disease test positive with rK39 rapid diagnostic tests.^16^^,^^17^ Such tests are also not suitable for assessment of test of cure or for diagnosis of relapse. A recent evaluation of the recombinase polymerase amplification had a high concordance with PCR based on peripheral blood sample^18^; such tool can serve as an alternative approach for disease confirmation and monitoring parasite load. Further development of existing proposed noninvasive tools for disease diagnosis and monitoring of treatment outcomes should be a research priority.^19^^–^^23^

There was a notable variability in the inclusion and exclusion criteria adopted across the studies, including the ranges of hemoglobin, platelets, and liver enzymes tests and coinfection with major comorbidities such as HIV or TB and hepatic, cardiac, and renal complications. Overall, these criteria will generally exclude patients who present with a severe kala-azar. This suggests that current evidence base for VL is derived from a patient population with a relatively mild–moderate form of VL. Innovative trial designs are required to provide further information on drug effectiveness in patient populations excluded from the standard therapeutic efficacy studies. In particular, there is a paucity of information regarding treatment in pregnancy,^24^ very young patients, and among patients with severe disease. Responsible inclusion of these patients in a pragmatic trial^25^ or the use of registries or observational/surveillance databases might facilitate further assessment of drug effectiveness in these excluded populations. In particular, large surveillance databases such as the Kala-Azar Management Information System (KAMIS)^26^ and Reportable Disease Information System-SINAN (Brazilian Ministry of Health)^27^ can provide critical information regarding the outcomes among the patient population excluded in trials, although this would require careful epidemiological assessment of different biases.

Several elements of the studies were poorly reported. For example, 40% of the randomized studies did not report the approaches used for sequence generation, 45% did not report on the allocation concealment, and 55% did not report details of sample size calculation. Age range of the patients included was also not reported in one-fifth of the studies. The primary endpoint was not explicit in 44% of the studies, and when it was stated and reported, different terminologies were used to refer to the same endpoints. For example, initial cure was most commonly defined as a composite of initial parasitological and clinical cure at the end of the therapy period in majority of the studies; alternative terminology adopted included initial apparent cure, apparent response, or cure (Table 4). Definitive cure assessed at the end of the study follow-up required demonstration of clinical and parasitological cure with absence of relapse; alternative terminologies used included ultimate cure, definite cure, final cure, and full cure (Table 4). These findings, taken together with observation from a previous review,^28^ suggest a need for harmonization of terminologies and reporting practices.

There was also variation in the time point at which drug efficacy was assessed. The majority of studies assessed the test of cure within 30 days of treatment completion and assessed definitive cure at 6 months. In particular, the assessment of definitive cure requires absence of relapse. Asymptomatic relapses^29^ or those with low-grade parasite load with nonpalpable spleen can be potentially misclassified as definitive cure. This can have important ramifications for VL control because disease relapse provides an infective parasite pool to sustain further transmission. It is thus important to develop an accurate diagnostic algorithm for identifying relapses or to identify patients at high risk of relapse after treatment; such an algorithm can be a complementary tool to aid in diagnosis in settings where it might be difficult to carry out aspiration or if long-term follow-up is not feasible.

Development of a clear case definition of VL for patient screening, defining severe disease, defining relapse and suspected case of relapse, and development of a standardized protocol for design and conduct of VL studies would help in harmonization of clinical practices. The global VL community has come together to develop an individual patient data platform that is hosted at IDDO.^30^ The IDDO data platform is currently being used to address some of the challenges identified in this review.^31^^,^^32^ Recently a checklist was developed for reporting malaria therapeutic efficacy studies.^33^ Lessons learned from such an initiative can also be applied in the context of VL. The development of Clinical Data Interchange Standards Consortium standards for capturing data in clinical studies through the IDDO-Drugs for Neglected Diseases initiative collaboration and the development of standardized case report forms are important steps in this direction.^34^ Such harmonization is important to maximize the information collected from trials, as conducting new therapeutic studies is becoming increasingly challenging in the Indian subcontinent because of the declining burden of the disease^35^ and in East Africa because of political instability.^36^

Some of the challenges identified in this review and potential solutions are summarized in Box 1. A reporting checklist has been developed based on the completeness of reporting of different aspects of trial design, conduct, analysis, and reporting identified in this review and is presented in Box 2.

**Box 1:** Key methodological challenges and potential solutions

Key methodological challenges	Potential solutions
Harmonization of terminologies	Development of a harmonized reporting checklist (see Box 2).
Lack of evidence regarding treatment efficacy among severe cases and those with complicated disease profile such as comorbidity	Use of observational/programmatic epidemiological data (KAMIS^26^) to understand the outcomes among the patient groups who are currently excluded in the trial settings. This can inform if future trials can broaden the inclusion range.
Optimal duration of post-treatment follow-up	Longitudinal studies with long-term follow-up required to assess the timing of relapse (such as KAMIS^26^). IDDO VL database can be explored to characterize the temporal trend of relapse during the follow-up phase of the study^30^; synthesis of aggregate data can provide further complementary insights.
Impact of LFU and those who withdraw/LAMA	Usually excluded in per protocol analysis leading to substantial loss in statistical power. Cured proportion currently used in studies but information from those who are LFU or those who are LAMA can be incorporated using survival analysis.^37^ Only two of the 89 studies adopted survival analysis as an approach for estimated drug efficacy.
Challenges in detecting asymptomatic relapses^29^	Identification of relapse is conditional on clinical suspicion. Therefore, asymptomatic relapses can be missed, and these can provide an infecting pool for onward transmission of the disease. Therefore, prompt identification and treatment of asymptomatic relapses remains crucial. Developing of highly specific molecular/serological based tests can help in identification of such cases. In the absence of such tests, understanding the determinants of asymptomatic relapses can be useful; this can potentially be explored using the IDDO database of clinical studies^30^ or through assessment of data generated from surveillance studies.
Defining relapse: reinfection or “true relapse”	PCR genotyping approach can potentially be used but limited availability in remote areas. Development of a diagnostic model for identification of key patient characteristics who are likely to relapse can provide complementary information for clinical decision making. Such efforts can be facilitated by the IDDO data platform and is currently being undertaken.^30^
Parasite quantitation (current parasite gradation is based on semi-quantitative range)	Molecular methodology can be adopted; Roy et al.18 evaluated recombinase polymerase amplification, which had a high concordance with PCR based on peripheral blood sample^18^; such a tool can serve as alternative approach for disease confirmation and monitoring parasite load in clinical studies.

IDDO = Infectious Diseases Data Observatory; LAMA = leave against medical advice; LFU = lost-to-follow-up; PCR = polymerase chain reaction; VL = visceral leishmaniasis.

**Box 2:** Suggested reporting checklist for therapeutic efficacy studies in visceral leishmaniasis

Manuscript Section	Item	Subitem	Description
Title	1	a	Identify the study design in the title (e.g., RCT, cohort)
Methodology			
Site description	2	a	Provide site details (country and city)
		b	Provide details of VL endemicity at study site
Study design	3	a	State the phase of the study
		b	Specify the aim of the trial: equivalence, noninferiority (with margin), superiority
Sample size calculation	4	a	Provide sample size calculation and desired statistical power including the endpoint adopted for sample size estimation
		b	State anticipated proportion of patients who are lost to follow-up
		c	Specify the margin for noninferiority trials
		d	Details of the software used for estimation of sample size
Study procedures	5	a	Details on informed consent/assent
		a	State if the study is randomized
		b	Describe details of sequence generation of randomized list (computer generated, block size, etc.)
		c	Describe details of blinding with details of who are blinded to allocation (investigators, patients, laboratory technicians)
Treatment details	6	a	Describe the total target dose and mode of administration (intramuscular, intravenous, oral)
		b	Specify drug manufacturer and batch number
		c	Specify the duration of infusion for intravenous or intramuscular drugs
		d	Specify if the administration of oral tablets were supervised
Inclusion and exclusion criteria	7	a	State case definition for patient screening
		b	Give eligible age range
		c	State any comorbidity that was excluded: malnutrition, HIV, tuberculosis, malaria, helminthic infections, hepatic conditions, cardiac conditions, retinal disease, endocrine disorders (including pancreatitis and diabetes), post–kala-azar dermal leishmaniasis, para-kala-azar
		d	State the inclusion of females and women susceptible to becoming pregnant, requirements of contraception usage including the length of usage
		e	State if the following are eligibility criteria: patients with history of VL, prior treatment, previously unresponsive cases
		f	Specify biological ranges: hemoglobin, white blood cell count, platelets, albumin, blood urea nitrogen, bilirubin, aminotransferase
Diagnostics for patient enrollment	8	a	State the causative parasite species (*Leishmania infantum*, *Leishmania donovani*)
		b	Method used for confirmation of VL (microscopy for detection of amastigotes, serology or molecular method for parasite DNA detection)
		c	Sample source used for VL confirmation (blood, tissue aspirate: spleen, bone marrow, lymph node)
		d	Staining used for microscopy (Giemsa, field, Leishman)
		e	State if promastigotes culture was used (including the details of the medium used)
		f	State if parasite quantitation was done including the methodology used (e.g., Chulay–Bryceson method)
		g	Details on quality control of the laboratory procedures used (external validation, double reading of slides, etc.)
		h	State if laboratory procedures were blinded
		i	Details regarding distinction between relapse and reinfection (if done)
Outcome definition	9	a	State and define the primary and secondary endpoints adopted
		b	State the definition of initial cure and definitive cure
		c	State the definition of relapse
		d	Describe the method used for confirmation of parasitological cure at test of cure assessment and at end of study follow-up
Patient follow-up	10	a	Details of the patient follow-up schedule including any prespecified visits
		b	State the time-point of assessment for test of cure and final outcome (e.g., clarity regarding if 6 months means 6 months since randomization or 6 months post-discharge from hospital)
Results			
Analysis and reporting	11	a	Clearly present the patient screening logs including the number screened and reasons for exclusion (CONSORT flow diagram)
		b	Clearly present the number of patients who are lost to follow-up, withdrawn, requiring rescue therapy
		c	Report all patient outcomes (relapse, failure, rescue therapy) and the time of their occurrence
		d	Report any adverse and severe adverse events and the time point of their occurrence
		e	Present the effect size estimate including the denominators used for calculation for proportions
		f	Clearly present how patient attrition are handled when calculating cured proportion (e.g., were they censored in survival analysis, excluded in available case analysis, or included in extreme case analyses)
		g	Clearly state and define the analysis principle adopted (intention-to-treat, per-protocol); clearly define the details of evaluable population, on-treatment population
		h	Present all the estimates with 95% CIs
		i	Give details on the rescue therapy used for treating relapses or unresponsive cases and the outcomes of the retreatment
		J	Clearly report the approach used for handling missing data (those with missing outcome, lost to follow-up)

CONSORT = Consolidated Standards of Reporting Trials; RCT = randomized controlled trial; VL = visceral leishmaniasis.

## CONCLUSION

This review highlighted substantial methodological variability in definitions adopted for patient screening, disease diagnosis, and therapeutic outcomes, suggesting a need for a harmonized protocol for design and conduct of VL efficacy studies. This is particularly important because there is a need for innovative trial designs to facilitate future drug development as undertaking randomized trials for novel drug regimens in VL remains a major operational challenge.

## Supplemental Materials

10.4269/ajtmh.23-0458 Supplemental Materials
