# Supplementary material for: Design, Conduct, Analysis, and Reporting of Therapeutic Efficacy Studies in Visceral Leishmaniasis: A Systematic Review of Published Reports, 2000–2021
Source: Am J Trop Med Hyg. 2024 Jul 2;111(2):365–76. doi: 10.4269/ajtmh.23-0458 (PMC11310606; doi:10.4269/ajtmh.23-0458)
Supplement: Supplemental Materials [file tpmd230458.SD1.pdf]

Supplemental file S1: Further details on the methodological aspects of the studies included

Manuscript: Design, conduct, analysis, and reporting of therapeutic efficacy studies in Visceral Leishmaniasis: A systematic review of published reports, 2000-2021

Prabin Dahal[1,2*], Sauman Singh-Phulgenda[1,2], Caitlin Naylor[1,2], Matthew Brack[1,2], Mitali Chatterjee[3], Fabiana Alves[4], Philippe J Guerin[1,2], Kasia Stepniewska[1,2]

Table of Contents

Supplemental table 1: List of studies included in the review (n=89 studies) 2

Supplemental table 2: Details of randomisation methods adopted in the studies included (n=40 studies) 6

Supplemental table 3: Details of inclusion and exclusion criteria adopted in the studies (n=89 studies) 11

Supplemental table 4: Methodology adopted for assessment of nutritional status (n=21 studies)........ 28

Supplemental table 5: Inclusion of patients with critically illness from Visceral Leishmaniasis (VL) or with severe Visceral Leishmaniasis (n=89 studies) 31

Supplemental table 6: Case definitions and case confirmation approach (n=89 studies) 35

Supplemental table 7: Reasons for patient exclusion in studies that reported patient flow (n=46 studies)........ 42

Supplemental table 8: Description of the studies with patients excluded for negative VL upon parasitological examination (n=13 studies)........ 44

Supplemental table 9: Diagnostic methods used for enrolment and outcome assessment for relapse (n=89 studies) 45

Supplemental table 10: Methodology adopted for outcome assessments (n=89 studies)........ 53

Supplemental table 11: Details of sample size estimation (n=34 studies) 59

Supplemental table 12: Details of analytical approach used for efficacy estimation (n=89 studies)........ 65

Supplemental table 1: List of studies included in the review (n=89 studies)

Author-year	PubMed ID/ PubMed Central ID	Country	Randomisation	Follow-up (days)	Enrolled	Treatment blinding	Age-range	Patients with HIV co-infections	Pregnancy
Gaeta-2000	11200380	Italy	Single Group	180	12	Unclear	All ages	Excluded	Not clear
Sundar-2000	11049800	India	Randomised	180	54	Open	All ages	Excluded	Excluded
Thakur-2000	11127250	India	Randomised	180	150	Open	All ages	Not clear	Not clear
Sundar-2000	11049798	India	Single Group	180	320	Unclear	All ages	Not clear	Not clear
Sundar-2000	10897369	India	Single Group	180	70	Unclear	All ages	Excluded	Excluded
Thakur-2000	11127251	India	Randomised	180	120	Open	All ages	Not clear	Excluded
Veeken-2000	10886792	Sudan	Non-randomised	180	516	Open	All ages	Not clear	Not clear
Villanueva-2000	11117648	Not stated	Non-randomised	1020	32	Unclear	Not clear	Included	Not clear
Thakur-2001a	11137652	India	Randomised	180	34	Unclear	All ages	Excluded	Excluded
Moore-2001	11417033	Kenya	Non-randomised	180	102	Open	All ages	Not clear	Not clear
Ritmeijer-2001	11816442	Ethiopia	Non-randomised	180	199	Open	All ages	Included	Not clear
Thakur-2001b	11280166	India	Single Group	180	309	Unclear	All ages	Not clear	Not clear
Dietze-2001	11791957	Brazil	Non-randomised	360	22	Open	All ages	Excluded	Excluded
Sundar-2001	11520836	India	Randomised	180	91	Open	Not clear	Excluded	Excluded
Haidar-2001	11426243	Yemen	Single Group	20	32	Unclear	Less than 15y	Not clear	Not applicable
Das-2001	11584934	India	Randomised	180	158	Unclear	All ages	Not clear	Not clear
Sundar-2002a	12456849	India	Randomised	180	398	Open	All ages	Excluded	Excluded
Sundar-2002b	12135284	India	Randomised	180	84	Blinded	All ages	Excluded	Excluded
Laguna-2003	12888588	Spain	Randomised	180	57	Open	Adults	Included	Excluded
Sundar-2003	12955641	India	Single Group	180	203	Open	All ages	Excluded	Excluded

Figueras Nadal-2003	14636517	Spain	Single Group	180	32	Open	Less than 15y	Excluded	Not clear
Sundar-2003	12792385	India	Non-randomised	180	39	Open	Less than 15y	Excluded	Not applicable
Syriopoulou-2003	12594635	Greece	Non-randomised	180	123	Open	Less than 15y	Excluded	Not applicable
Rijal-2003	15228258	Nepal	Single Group	180	120	Open	All ages	Excluded	Not clear
Thakur-2004	15035723	India	Not Specified	180	120	Unclear	All ages	Excluded	Not clear
Sundar-2004	14727208	India	Randomised	180	153	Open	All ages	Excluded	Excluded
Thakur-2004b	15489554	India	Not Specified	180	282	Unclear	All ages	Excluded	Not clear
Bhattacharya-2004	14699453	India	Single Group	180	80	Open	Less than 15y	Excluded	Excluded
Wasunna-2005	16282296	Kenya	Non-randomised	180	97	Open	All ages	Excluded	Excluded
Jha2005	16354802	India	Randomised	180	120	Open	All ages	Excluded	Excluded
Das2005	16130613	India	Single Group	180	182	Unclear	All ages	Not clear	Not clear
Ritmeijer-2006	16804852	Ethiopia	Randomised	180	580	Open	All ages	Included	Not applicable
Sundar-2006	16447104	India	Randomised	180	405	Open	All ages	Excluded	Excluded
Singh-2006	17202605	India	Single Group	180	64	Unclear	Less than 15y	Excluded	Not applicable
Singh-2006b	17202633	India	Randomised	180	125	Unclear	Less than 15y	Excluded	Not applicable
Mueller-2006	16730363	Sudan	Single Group	30	64	Unclear	All ages	Included	Excluded
Sundar-2007	17682988	India	Randomised	180	1485	Open	All ages	Excluded	Excluded
Sundar-2007b	17582067	India	Randomised	180	667	Open	All ages	Excluded	Excluded
Bhattacharya-2007	17624846	India	Single Group	180	1132	Open	All ages	Excluded	Excluded
Sundar-2008	18781879	India	Randomised	270	226	Open	All ages	Excluded	Excluded
Sundar-2008b	18664241	India	Non-randomised	180	45	Open	All ages	Excluded	Excluded
Thakur-2008	18765878	India	Randomised	180	140	Blinded	All ages	Not clear	Not clear
Mueller-2008	18186974	Uganda	Non-randomised	180	371	Unclear	All ages	Not clear	Not clear

Sundar-2009	19407109	India	Non-randomised	180	60	Open	Adults	Excluded	Excluded
Das-2009	19436614	India	Randomised	180	82	Open	All ages	Excluded	Excluded
Sundar-2009b	19663597	India	Randomised	180	329	Open	All ages	Excluded	Excluded
Adam-2009	19766208	Sudan	Single Group	365	42	Unclear	All ages	Excluded	Excluded
Shahian-2009	19478699	Iran	Single Group	180	20	Unclear	Less than 15y	Excluded	Not applicable
Hailu-2010	21049059	Sudan, Kenya, Ethiopia	Randomised	180	270	Open	All ages	Not clear	Excluded
Thakur-2010	21036834	India	Randomised	360	230	Open	All ages	Excluded	Not clear
Musa-2010	21049063	Sudan	Randomised	180	42	Open	All ages	Excluded	Excluded
Sundar-2010	20147716	India	Randomised	180	412	Open	All ages	Excluded	Not clear
Mondal-2010	20668544	India	Randomised	180	25	Open	All ages	Excluded	Excluded
Rijal-2010	19726065	Nepal	Single Group	180	198	Unclear	All ages	Not clear	Not clear
Singh-2010	20065047	India	Randomised	180	605	Unclear	Less than 15y	Excluded	Not applicable
Sinha-2010	20682882	India	Single Group	180	251	Unclear	All ages	Excluded	Excluded
Sundar-2011	21255828	India	Randomised	180	634	Open	All ages	Excluded	Excluded
Sundar-2011b	21633025	India	Randomised	180	61	Open	All ages	Excluded	Excluded
Sundar-2011c	21129762	India	Single Group	180	135	Unclear	All ages	Excluded	Excluded
Rahman-2011	21734127	Bangladesh	Single Group	180	977	Open	All ages	Excluded	Excluded
Sinha-2011	22174722	India	Single Group	180	494	Open	All ages	Excluded	Excluded
Sudarshan-2011	21609983	India	Randomised	46	46	Unclear	All ages	Excluded	Not clear
Sundar-2012	22573856	India	Single Group	180	567	Open	All ages	Excluded	Excluded
Musa-2012	22724029	Sudan, Ethiopia, Kenya, Uganda	Randomised	180	972	Open	All ages	Included	Excluded
Patra-2012	23087513	India	Single Group	180	71	Unclear	All ages	Excluded	Excluded
Rijal-2013	23425958	Nepal	Single Group	360	120	Unclear	All ages	Included	Excluded
Khalil-2014	24454970	Ethiopia, Sudan	Randomised	180	124	Open	Not clear	Excluded	Excluded
Ostyn-2014	24941345	India, Nepal	Single Group	360	1016	Unclear	All ages	Excluded	Not clear
Cota-2014	24743472	Brazil	Non-randomised	180	90	Unclear	All ages	Included	Not clear
Sundar-2014	25233346	India	Randomised	180	500	Open	All ages	Excluded	Not clear

Mondal-2014	25104636	Bangladesh	Single Group	180	300	Unclear	All ages	Not clear	Excluded
Jamil-2015	26496648	Bangladesh	Single Group	180	120	Open	All ages	Excluded	Excluded
Sundar-2015	25510715	India	Non-randomised	180	30	Open	All ages	Excluded	Excluded
Goswami-2016	26526926	India	Randomised	180	120	Open	All ages	Excluded	Excluded
Wasunna-2016	27627654	Kenya, Sudan	Randomised	210	183	Open	All ages	Excluded	Not applicable
Pandey-2016	27645786	India	Single Group	270	646	Open	All ages	Excluded	Excluded
Borges-2017	28327804	Brazil	Randomised	180	101	Open	Less than 15y	Not clear	Not applicable
Rahman-2017	28558062	Bangladesh	Randomised	180	602	Open	All ages	Excluded	Excluded
Romero-2017	28662034	Brazil	Randomised	180	378	Open	All ages	Excluded	Excluded
Alborzi-2017	27879460	Iran	Randomised	180	75	Open	All ages	Not clear	Not clear
Pandey-2017	29016288	India	Single Group	180	100	Open	Not clear	Excluded	Not clear
Kimutai-2017	PMC5315726	Sudan, Ethiopia, Kenya, Uganda	Single Group	180	3126	Open	All ages	Included	Included
Mbui-2018	30188978	Kenya, Uganda	Single Group	210	30	Open	Less than 15y	Excluded	Excluded
Goyal-2018	30346949	India	Non-randomised	180	1761	Open	All ages	Excluded	Included
Sundar-2019	31436156	India	Single Group	360	1143	Unclear	All ages	Excluded	Not Clear
Diro-2019	PMC6336227	Ethiopia	Randomised	58	58	Open	Adults	Included	Excluded
Sinha-2019 [a]	Not indexed	India	Single Group	208	160	Open	Less than 15y	Excluded	Excluded
Goswami-2020	32394874	India	Randomised	1825	154	Open	All ages	Excluded	Excluded
Ekram-2021	34789971	Bangladesh	Single Group	180	31	Open	All ages	Excluded	Excluded

[a]Kishore Kumar Sinha, Ashish Basant, Amarjeet Patel. A Study on Efficacy and Tolerability of Miltefosine for Childhood Visceral Leishmaniasis in a Tertiary Care Centre in Eastern Bihar. Indian Journal of Applied Research. Volume-9 | Issue-2 | February-2019 | PRINT ISSN - 2249-555X.

Supplemental table 2: Details of randomisation methods adopted in the studies included (n=40 studies)

Author-year	Randomisation details	Sequence generation	Allocation Concealment
Ritmeijer-2006	The patient was randomized to receive miltefosine or SSG according to a computer-generated number list. The allocation ratio was 1:1. The study was unblinded; miltefosine is oral medication and SSG is injection medication.	Computerised	Unclear
Thakur-2001a	Matched for age and sex, the patients were randomly allocated into two treatment groups.	Unclear	Unclear
Laguna-2003	A randomization list was prepared using the SAS program, which stratified patients into two groups, depending on the CD4 cell count at inclusion: above or below 200 cells/mm^3. If this information was missing at the time of randomization, it was considered equivalent to the lymphocyte count: above or below 1000 cells/mm^3. The randomization process was blinded and centralized.	Computerised	Unclear
Sundar-2008b	An independent statistician generated a randomization schedule by use of a computer-based procedure; assumptions were a maximum number of 60 patients enrolled per arm (240 total patients) and 15 randomization blocks with a size of 15 patients each. Sealed randomization envelopes were prepared, and treatment was begun within 72h after diagnosis by splenic aspirate.	Computerised	Sealed envelope
Sundar-2002b	Patients were centrally registered and randomly assigned at each site to miltefosine or amphotericin therapy in a 3:1 ratio with the use of permuted blocks of four patients each.	Unclear	Unclear
Sundar-2000c	After completing initial diagnostic and baseline laboratory testing, 54 patients were randomized, by means of a computer- generated, sealed-envelope method, to receive one 50-mg capsule of miltefosine twice daily with meals for either 14 (group A), 21 (group B), or 28 days (group C).	Computerised	Sealed envelope
Das-2009	Treatment allocation was done by the biostatistician of the institute, who performed the allocation sequence using random number tables and accordingly assigned the test and control group. A total of 82 SSG unresponsive and parasitologically confirmed VL cases were divided randomly into two groups before the initiation of the treatment.	Random number table	Unclear
Thakur-2000a	Unclear	Unclear	Unclear
Jha-2005	Patients were randomized contemporaneously to receive sitamaquine daily for 28 days at one of four doses. The randomization schedule provided for an equal number of subjects in all four cohorts. However, to minimize the number of patients exposed to higher sitamaquine doses, the randomization schedule was not followed for the final block of 8 subjects (Subjects 113 to 120). These 8 subjects were entered into Cohorts 1 and 2.	Unclear	Unclear

Sundar-2006	An independent statistician prepared randomization envelope by use of a computer-generated random-number generator. The sealed envelopes were then distributed to the enrolled subjects, to randomly assign them to 1 of the 3 following ABCD total-dose groups: 7.5 mg/kg (group A), 10 mg/ kg (group B), and 15 mg/kg (group C).	Computerised	Sealed envelope
Sundar-2004	An independent statistician prepared randomization envelope using a computer-based random number generator. Enrolled subjects were randomly assigned by sealed envelope to receive 3 treatments.	Computerised	Sealed envelope
Sundar-2007b	For the purposes of this study, 15 alternate-day infusions of 1 or 0.75 mg/kg (groups A and B) were considered as conventional therapy. To compare responses to these same doses given once daily (groups C and D), we used a 1:2 ratio for random assignment to treatment arms and aimed to enrol 250 subjects each in groups A and B and 500 subjects each in groups C and D. An independent statistician prepared sealed randomization envelopes using a computer-based random number generator.	Computerised	Sealed envelope
Thakur-2008	Of the 181 patients screened, only 140 met the inclusion and exclusion criteria and gave written consent. These were divided randomly in two groups with 70 patients, each matched for age and sex; 55 men and 15 women were included in each group (Fig. 2). As there were some difficulties in culturing grade-1 amastigotes, patients with grade-1 amastigotes were excluded from both the groups to maintain parity.	Unclear	Unclear
Sundar-2007a	Enrolled patients were randomly assigned to treatment with paromomycin or amphotericin in a 3:1 ratio in permuted blocks of four. A fraction of the patients in the paromomycin group were also randomly assigned to a sub study in which pharmacokinetic sampling was performed.	Unclear	Unclear
Sundar-2002a	Patients were randomized into preassigned treatment groups by the sealed-envelope technique, and liposomal amphotericin B was administered by an independent coinvestigator who broke the seal of the envelope and prepared infusions	Unclear	Sealed envelope
Sundar-2009b	Patients were allocated to a randomization arm from a random table generated for this purpose.	Unclear (random number tables)	Unclear
Sundar-2001	Participants were randomised by sealed envelope to receive 5 mg/kg of liposomal amphotericin as a single infusion or as once daily infusions of 1 mg/kg for five consecutive days. An independent statistician prepared the randomisation envelopes using a computer-based random number generator. The study staff opened consecutively numbered envelopes containing the treatment assignment after eligible patients fulfilled the entry criteria.	Computerised	Sealed envelope
Thakur-2000b	Patient eligibility was evaluated before randomisation to treatment with a computer-generated randomisation list.	Computerised	Unclear

Singh-2006b	Patients were randomized into four groups by slips kept separately in two small boxes for both newly diagnosed patients and those who had received 30 days course of sodium antimony gluconate (SAG).	Unclear	Boxed
Das-2001	The patients were randomly allocated to two regimen groups, combination regimen of pentamidine (half dose) and allopurinol, and single regimen of pentamidine alone.	Unclear	Unclear
Sundar-2011b	A computer-generated, randomisation code was generated by the trial statistician by use of SAS 9.1 (SAS Institute, Cary, NC, USA). To ensure maximum balance of the numbers in each group at any time and to minimise bias, block sizes of 16 were generated for treatment allocation of patients to one of the four treatment groups, with equal allocation ratio and independently for each site. Individual, opaque, sealed, and sequentially numbered envelopes were provided to each trial site, one envelope per patient, to indicate the allocation of individual patients to treatment.	Computerised	Sealed envelope
Sundar-2011c	Patients were randomized into blocks of 12 in the ratio 1:1:1: 1:2 to one of four sitamaquine cohorts or Ambisome. Treatment was allocated by using the GlaxoSmithKline Registration and Medication Ordering System (RAMOS).	Computerised	Unclear
Hailu-2010	Allocation to treatment was by means of sequentially numbered, sealed envelopes, generated from a computerized randomization list. Each centre received a box of uniquely numbered sealed envelopes from the LEAP Trial Coordination Centre in Nairobi, where centralized randomization and envelope preparation were carried out in blocks of 15 to maintain randomization balance within centres.	Computerised	Sealed envelope
Thakur-2010	This study was conducted as an open-label, randomized trial of 230 patients at Balaji Utthan Sansthan, Patna. The study staff who treated the patients opened consecutively numbered envelopes containing the treatment assignment after eligible patients fulfilled the entry criteria. Clinicians who provided treatment were not blinded to the treatment given.	Unclear	Sealed envelope
Musa-2010	Randomization was done using sequentially numbered sealed envelopes that were prepared according to a centrally generated randomization list.	Unclear	Sealed envelope
Khalil-2014	Patients were randomized to receive either treatment using a computer-generated randomisation list, stratified by site. Individual treatment allocations were placed in sealed, opaque envelopes which were opened after a patient had been entered into the trial.	Computerised	Sealed envelope
Sundar-2010	To compare responses to liposomal amphotericin B versus amphotericin B deoxycholate, we used a 3:1 ratio for random assignment to treatment, aiming to assign 300 patients to receive liposomal amphotericin B (liposomal-therapy group) and 100 to receive amphotericin B deoxycholate (conventional-therapy group). An independent statistician prepared sealed randomization envelopes, using a computer-based random-number generator.	Computerised	Sealed envelope

Musa-2012	A computer-generated randomization list was produced with stratification by centre and block sizes of 15 until recruitment in the PM arm was completed, and block sizes of 10 thereafter. Allocation was concealed using opaque, sequentially numbered sealed envelopes. The randomization list and envelopes were prepared and stored securely at the LEAP Data Centre, based at the trial coordination centre in Nairobi.	Computerised	Sealed envelope
Mondal-2010	An independent statistician prepared randomization envelops by the use of a computer-generated random number table. The sealed envelopes were then distributed to the enrolled subjects, to randomly assign them to one of the following total dose groups; 5 mg/kg single shot (n = 10), 7.5 mg/kg single shot (n = 10) and 5 mg/kg double shot (total 10 mg/kg) (n = 10).	Computerised	Sealed envelope
Singh-2010	They were randomized into two treatment groups, Groups A and B by electronically generated random table.	Computerised	Unclear
Sudarshan-2011	No information.	Unclear	Unclear
Goswami-2016	Patients were randomized to receive either treatment using a computer-generated randomization list. Individual treatment allocations were placed in sealed, opaque envelopes, which were opened after a patient had been entered into the trial. It was not possible to blind patients or treating physicians because of the nature of the intervention.	Computerised	Sealed envelope
Sundar-2014	The permuted block randomization, with block size of 4, and ratio of 3:1 in the two groups were generated for each centre. Eligible patients were sequentially allotted to unique subject ID and treatment as per randomization schedule for that centre. The screening and randomization log were maintained.	Unclear	Unclear
Diro-2019	Subjects were allocated to treatment using random block sizes, stratified by site and by patient type (whether the VL episode at screening was a primary or relapse case). The randomization list was prepared by the data management team. Site investigators were blinded to block sizes. Randomization codes were prepared in sealed, sequentially numbered, opaque envelopes and were under the control of the site investigator.	Unclear	Sealed envelope
Borges-2017	Randomization procedure was performed in blocks of 20, using Graphpad Quickcalcs free software (GraphPad Software Inc., San Diego, CA). This procedure was under the responsibility of an independent researcher from Tropical Medicine Center at Universidade de Brasília, who was not directly involved in any other operational aspect of the study. The names of compared drugs were typed on a sheet and placed in dark envelopes, which were sealed, stamped, and signed by those responsible for the randomization. The envelopes were opened immediately after the consent of the participants.	Computerised	Sealed envelope
Wasunna-2016	Subjects were randomly allocated using block randomization, stratified by site. Site investigators were blinded to block size and codes were concealed in sealed sequentially numbered, opaque envelopes under the control of the site investigator.	Unclear	Sealed envelope

Rahman-2017	A computer-generated randomization code was used for patient treatment allocation. Individual, opaque, sealed and sequentially numbered envelopes were provided to each study site (one envelope per patient), indicating the individual patient allocation to treatment.	Computerised	Sealed envelope
Romero-2017	Computer-generated randomization into the four treatment arms was done using the software Quickcalcs-online calculators for scientists (www.graphpad.com/quickcalcs/randomize1.cfm). Blocks of28 treatment allocations were generated and placed in sealed, opaque envelopes that were sent to each clinical trial site, and only opened by trial clinicians or site investigators when a participant was included in the trial. After the AmphoB arm was withdrawn, if an enrolled patient was allocated to this treatment arm, the subsequent envelope containing a new code was designated to the patient until they were allocated to any of the three remaining arms. This type of approach did not allow for blinding.	Computerised	Sealed envelope
Goswami-2020	A computer-generated, randomization code was generated. Enrolled patients were randomly assigned to treatment with Miltefosine or with the combination chemotherapy in a 1:1 ratio. Microscopist were masked to the treatment given.	Computerised	Unclear
Alborzi-2017	The patients were randomized into three groups regardless of age, sex, and duration of the disease.	Unclear	Unclear

Supplemental table 3: Details of inclusion and exclusion criteria adopted in the studies (n=89 studies)

**Author year**	**Inclusion/Exclusion criteria**
Gaeta-2000	HIV-negative individuals over 18 y were eligible if Leishmania was visible in, or cultured from, a bone marrow aspirate
Sundar-2000c	Inclusion and exclusion criteria and baseline laboratory tests (as well as all trial procedures) were identical to those used in our prior studies of Miltefosine and are described in detail elsewhere. Patients were excluded from the study if they had another serious concurrent disease or infection or if they were <12 years of age or >65 years of age, were pregnant or breast-feeding, or were determined to be seropositive for HIV by means of ELISA testing (no screened subject was seropositive). Other exclusion criteria were a WBC count of <2000 cells/mm3, a haemoglobin level of <6 g/dL, a platelet count of <50,000 cells/mm3, levels of hepatic transaminases or total bilirubin >3 times the normal levels, a serum creatinine level of>2 mg/dL, and a prothrombin time >5 s above the control value.
Thakur-2000a	Refer to JHA et al. (1998) (IDDO TAG 69) for a complete description of materials and methods employed in this study.<br>From Jha-1998: Exclusion criteria were a known allergy to aminoglycosides, treatment in the previous 12 months with a drug with recognised or presumed anti-leishmanial action, serious concomitant diseases, pregnancy or lactation (women underwent a pregnancy test at initial assessment), failure to agree to return for all follow up evaluations, and being critically ill from leishmaniasis. Definitions for critical illness from leishmaniasis included the spleen reaching to the pelvic crest, haemoglobin concentration < 50 g/l, white cell count < 2 × 109/l,3 platelet count < 80 × 109 /l,3 aspartate aminotransferase concentration > 4 × upper limit of normal, serum albumin concentration < 20 g/l, and urine urea or creatinine concentration > 2 × upper limit of normal.
Sundar-2000b	Male and female patients of any age were eligible for the study if they had typical symptoms and signs of kala-azar (e.g., fever, weight loss, hepatosplenomegaly, and pancytopenia), if they had characteristic amastigotes observed microscopically on splenic or bone marrow aspirate smears, and if they had not received prior antileishmanial treatment.<br>Patients were excluded from this study if they had a serious underlying illness (e.g., congestive heart failure, renal dysfunction, or diabetes) or an associated infection (e.g., tuberculosis or malaria), if they were receiving immunosuppressive therapy, or if one of the following laboratory abnormalities was present: a serum glutamic oxaloacetic transaminase value >5 times the normal value, a serum creatinine level >2.0 mg/dL, or a prothrombin time >5 seconds above the control value.
Sundar-2000a	Patients were eligible for the study if they (a) had symptoms or signs of visceral leishmaniasis (fever, weight loss, splenomegaly),<br>(b) had amastigotes demonstrated on splenic aspirate smear, and (c) had not received antileishmanial therapy in the previous 3 months. Patients were excluded if they were aged <5 or >65 years, had a history of cardiac disease, were pregnant, had a serious concurrent infection such as tuberculosis or a serious bacterial pneumonia, or if 1 of the following laboratory abnormalities was present: granulocyte count: granulocyte count< 1000/mm3, haemoglobin <6 g/dL, platelet <50 000/mm3, prothrombin time >4 s above control values, bilirubin >2.0 mg/dL, serum glutamic oxaloacetic transaminase (SGOT) >200 IU, or creatinine >2.0 mg/dL. Together, these criteria would probably acetic transaminase (SGOT) >200 IU, or creatinine >2.0 mg/dL. None of the 70 enrolled subjects was HIV seropositive.

Thakur-2000b	Patients aged 6-50 years with symptoms and signs suggestive of visceral leishmaniasis and aspirates of spleen or bone marrow positive for leishmania amastigotes were eligible for inclusion in the study if they gave signed informed consent. Exclusion criteria were a known allergy to aminoglycosides, treatment in the previous 12 months with a drug with recognised or presumed anti-leishmanial action, serious concomitant diseases, pregnancy or lactation (women underwent a pregnancy test at initial assessment), failure to agree to return for all follow up evaluations, and being critically ill from leishmaniasis. Definitions for critical illness from leishmaniasis included the spleen reaching to the pelvic crest, haemoglobin concentration < 50 g/l, white cell count < 2 × 109 /l3, platelet count < 80 × 109 /l3, aspartate aminotransferase concentration > 4 × upper limit of normal, serum albumin concentration < 20 g/l, and urine urea or creatinine concentration > 2 × upper limit of normal.
Veeken-2000	The following clinical case definition was used: patients with fever for more than two weeks (with exclusion of malaria), in combination with either splenomegaly or wasting. In cases meeting the case definition, kala-azar was confirmed by a ≥ 1:6400 titre of antibodies against Leishmania by the Direct Agglutination Test (and a subsequent response to antimonial treatment); or by demonstration of Leishmania on aspirates of spleen or lymph node. Patients who had received any treatment for kala-azar in the past were excluded. Nomadic patients, in whom follow up would be impossible, were excluded. Informed written consent was given by the patient or their guardian/parent. Participation in the study was voluntary, and those who did not participate were treated with Pentostam. Patients were not excluded from treatment on the basis of severe disease. Malnourished patients received supplemental or therapeutic feeding according to severity.
Villanueva-2000	Concomitant infections were present in 11 patients: tuberculosis (4); Pneumocystis carinii pneumonia (PCP) (2); PCP plus esophageal candidiasis (1); PCP plus tuberculosis and Kaposi sarcoma (1); pneumococcal pneumonia (1);<br>Escherichia coli bacteraemia (1); and sinusitis (1).<br>Inclusion:<br>-Patients diagnosed with VL and HIV-1 coinfection between November 1996 and February 1999.<br>-Patients were considered positive for HIV infection if they had both a positive enzyme immunoassay and Western blot assay for HIV-1.<br>-Visceral leishmaniasis was diagnosed when amastigotes were identified in bone marrow aspirate smears on Giemsa staining or when growth of promastigotes occurred in bone marrow samples cultured in Novy-McNeal-Nicolle (NNN) medium.
Thakur-2001a	HIV positive cases, cases complicated with tuberculosis and renal, liver and heart diseases, patients unable to follow the protocol in all phases of study, pregnant and lactating women, refusal to give informed consent and cases previously treated with Ambisome were excluded. Certain precautions were taken in both the groups before starting treatment. The treatment was started only when the haemoglobin was above 5 g/dl, serum electrolytes deficiency, if any, was corrected [22], and myocardial damage assessed by ECG changes caused by prior treatment with sodium antimony gluconate stabilised [23]. It was decided to withdraw patients from the trial if they developed serious side effects.
Moore-2001	It may be noted that patients were included even if they had severe kala-azar, or comorbidity such as anaemia or cachexia. Pregnancy, breastfeeding, advanced age and infancy were not reasons for exclusion. Patients with a past history of kala-azar or who had received any antimonial in the past were excluded. Patients who were clinically suffering from kala-azar but had both a negative DAT test and a negative splenic aspirate were also excluded.

Ritmeijer-2001	Patients previously treated for VL were excluded. Participation in the study, including HIV testing, was voluntary and patients would receive treatment with Pentostam if they were to decline. HIV status was tested with 2 rapid tests. HIV testing was not possible for all patients: some patients died before blood was drawn, and during one period of the study no test kits were available.<br>Patients were identified using a modified WHO clinical case definition (WHO, 1996; MSF, 1999) of fever for >2 weeks, with exclusion of malaria, in combination with either splenomegaly or wasting. In cases meeting the case definition, VL was confirmed by a high titre Leishmania Direct Agglutination Test (DAT; titre 2 1: 6400). In cases with a borderline (1: 800- 1: 6400) or negative DAT (~1: 800), splenic aspiration was performed. Slides of splenic aspirates were checked by an independent microscopist at a later date. Given the harsh field conditions with failures in the cold chain and DAT antigen supply, the clinicians sometimes treated patients on clinical grounds, without parasitological or DAT confirmation.
Thakur-2001b	Patients with renal and cardiac complication were excluded.
Dietze-2001	The exclusion criteria included clinical contraindication to splenic aspiration, any history of anti-Leishmania therapy, evidence of serious underlying disease (cardiac, renal, hepatic, or pulmonary), including serious infection other than VL, acquired immunodeficiency syndrome or antibody to human immunodeficiency virus (HIV), severe protein and/ or caloric malnutrition (kwashiorkor or marasmus), glucose- 6 phosphate dehydrogenase deficiency, pregnancy, haemoglobin concentration < 5 g/100 mL, white blood cell count <1,000, platelet count < 30,000/mm3 , and a significant (> 3 times upper limit of normal) deviation in serum chemistries such as blood urea nitrogen, creatinine, alanine aminotransferase, and aspartate aminotransferase.
Sundar-2001	Patients of any age with visceral leishmaniasis caused by L donovani were eligible if they had symptoms or signs of the disease (fever, weight loss, splenomegaly) and parasites were found by micros- copy in a splenic aspirate smear. Patients were excluded if they were pregnant or breast feeding, HIV positive, had a serious concurrent infection such as tuberculosis or bacterial pneumonia, or if they had a granulocyte count < 1×109/l, haemoglobin concentration < 40 g/l, or platelet count < 40×109/l. Eight patients were excluded by these criteria.
Das-2001	Inclusion: VL patients unresponsive to antimony. Those patients who were critically ill, aged below five years and above 60 years, haemoglobin concentration below 40 g/1, having concurrent TB, renal impairment, cardiac disease or jaundice were excluded from the trial.
Sundar-2002	Potentially eligible patients were 12 years of age or older with visceral leishmaniasis suspected on the basis of clinical presentation (fever, splenomegaly, and cytopenia) and diagnosed by the presence of leishmania in splenic aspirates).<br>Criteria for exclusion were a platelet count below 50,000 per cubic millimeter, a white-cell count below 1000 per cubic millimeter, a hemoglobin concentration of less than 6 g per deciliter, results on liver-function tests (serum aspartate aminotransferase and alanine aminotransferase concentrations) more than three times the upper limit of normal, a bilirubin concentration more than twice the upper limit of normal, serum creatinine or blood urea nitrogen values more than 1.5 times the upper limit of normal, other major medical illness including human immunodeficiency virus infection or severe malaria, pregnancy or lactation, inability to maintain use of contraception for the period of treatment plus two months, and previous therapy with amphotericin B.
Sundar-2002a	Patients of any age or sex were eligible for enrolment into the trial if they had signs and symptoms of VL confirmed by the presence of parasites in splenic or marrow smears if they had failed to respond or VL relapsed after a full course of Sbv treatment. Pregnant or lactating women, HIV-positive patients, and intravenous drug abusers were excluded from the trial.
Laguna-2003	Exclusion criteria included patients with pancreatitis, prothrombin activity <40%, aminotransferase levels 10× the upper normal limit, myocardiopathy, heart failure, a Qt corrected interval >500 ms, creatinine levels >twice the upper normal limit, allergy to either ABLC or meglumine

	antimoniate, concomitant treatment with dideoxy- cytidine or dideoxyinosine and a life expectancy of <6 months. Women of childbearing potential were excluded if they were pregnant, might become pregnant, or were lactating. Active opportunistic infections were not an exclusion criterion.
Sundar-2003b	A total of 203 patients participated. Patients of all ages and both sexes were potentially eligible if they had symptoms and signs suggestive of VL (i.e., fever with chills, rigor, and splenomegaly) with demonstrable Leishmania parasites in splenic or bone marrow aspirate smears. Pregnant and lactating women, HIV-positive individuals, and individuals receiving concomitant antileishmanial drugs were excluded from the study. Patients were considered to have prior treatment failure if they had received adequate antileishmanial treatment, consisting of either sodium antimony gluconate (SAG; also known as sodium stibogluconate) at a dosage of 20 mg/kg for 30 days or 15 infusions of AmB administered at a dosage of >= 0.75 mg/kg.
Figueras Nadal-2003	Children between 0 and 14 years old diagnosed with visceral leishmaniosis through direct visualization of Leishmania amastigotes by Giemsa staining OR presence of Leishmania promastigotes in bone marrow aspirate culture OR detection of antibodies through indirect immunofluorescence (>1/80) OR ELISA analysis (>0,80). Exclusion: a) Previous or concomitant treatment with amphotericin B, antimonials derivatives or other drug used for the treatment of visceral leishmaniasis in the year prior to the enrolment in the study; b) Immunosuppression.
Sundar-2003	Children between the ages of 2 to 11 years with signs and symptoms suggestive of VL and demonstrable parasites in splenic aspirates were screened for eligibility in the study. Those with haemoglobin <6 g/dl, leukocyte count <2000/nl, platelet count <50 000/ nl, blood urea nitrogen (BUN) or serum creatinine >1.5 times of upper normal limit and alanine aminotransferase (ALT) >3 times upper limit was excluded from the study. Patients with HIV positivity or any concomitant non-compensated renal, hepatic, malignant or infectious diseases were excluded. Because ophthalmologic evaluation was to be done, children with retinal diseases were also excluded.
Syriopoulou-2003	Eligible participants were children up to 14 years of age who had clinical features that were consistent with active VL (e.g., fever, splenomegaly, anaemia, leukopenia, or thrombocytopenia) and who had visible parasites or amplified Leishmania DNA detected on bone marrow aspirates. Children with concomitant disease, known allergy to study compounds, known HIV seropositivity, or other immunosuppression, and children who had previously received treatment for leishmaniasis were excluded from the study.
Rijal-2003	Parasitologically proven VL cases with no history of previous treatment with SSG were included after obtaining informed consent from the patient or his/her guardian. Only patients from the 3 neighbouring districts of Sunsari, Morang, and Saptari were included as follow-up would not be practically possible for patients coming from more remote districts. There were no other exclusion criteria.<br>All the patients were negative to HIV testing
Thakur-2004a	Only patients who provided informed consent (or that of their guardians, if they were children) were enrolled. Patients were excluded if they had <6.0 g haemoglobin/dl blood, complications such as pneumonia, jaundice, tuberculosis, renal or cardiac disease, diabetes or HIV infection, serum concentrations of aspartate and/or alanine aminotransferase that were at least three times the upper limit of normal, a serum concentration of creatinine that was more than 1.5 times the upper limit of normal or had already received treatment with antimony, pentamidine, AMB or any other antileishmanial drug. Patients found to be infected with hookworm, Ascaris and/or Entamoeba histolytica/E. dispar were not excluded, as these parasites are very common in the study area but were treated appropriately (concurrently with antileishmanial treatment).

Sundar-2004	Patients were eligible if they had symptoms and signs of VL (fever, weight loss, and splenomegaly) and if microscopic analysis of a splenic aspirate smear revealed parasites.<br>Patients were excluded if they were pregnant or breast-feeding, were HIV seropositive, or had a serious concurrent infection, such as tuberculosis or bacterial pneumonia. Exclusion criteria also included a granulocyte count of <1000 granulocytes/mm3, a haemoglobin level of <3.5g/dL, or a platelet count <40,000 platelets/mm3; hepatic transaminase or total bilirubin levels that were >3 times the upper limit of normal; a serum creatinine level of 12.0 mg/dL; or a prothrombin time of >5 s above the control time
Thakur-2004b	Patients positive for HIV and other co-infections (n=28) were excluded.
Bhattacharya-2004	Potentially eligible patients were 2–11 years of age, were of either sex, and had VL suspected on the basis of clinical presentation (fever, splenomegaly determined by palpation, anaemia, and cytopenia) and confirmed by the demonstration of amastigotes in splenic aspirate specimens. Exclusion criteria were values for the formed elements of the blood suggesting a premorbid state and severe disease (platelet count, <50,000 platelets/mL; leukocyte count, <1000 leukocytes/mL; haemoglobin concentration, <6 g/dL; serum aspartate aminotransferase (ASAT) or alanine aminotransferase levels of >3 times the upper limit of normal; bilirubin level of more than twice the upper limit of normal; and serum creatinine or blood urea nitrogen levels of >1.5 times the upper limit of normal), documentation of other serious coincidental medical illnesses, HIV seropositivity, and, for patients capable of reproduction, pregnancy
Wasunna-2005	Subjects eligible for inclusion had a clinical diagnosis of VL with symptomatic disease and the diagnosis confirmed by the presence of Leishmania amastigotes in splenic aspirates. Subjects 5−65 years of age of either sex were included in the study.<br>Females that were pregnant or lactating were excluded. Additional exclusion criteria included known hypersensitivity to sitamaquine, use of an anti-leishmanial agent within 10 days or of an investigational compound within 30 days (or 5 half-lives) of the start of the study, subjects with marked deviations in serum chemistry or abnormal hematologic markers outside those expected to be caused by VL, patients with serious underlying disease, including human immunodeficiency virus (HIV) infection, malnutrition, or severe kala-azar, glucose-6-phosphate dehydrogenase deficiency, or previous inclusion in this study.
Jha-2005	Subjects aged 5−65 years of either sex were included in the study. Females of child-bearing age had to have a negative pregnancy test and agree to practice effective contraception throughout the study. Exclusion criteria included known hypersensitivity to sitamaquine, receipt of an antileishmanial agent within 10 days or of an investigational compound within 30 days (or 5 half-lives) of study start, sub- jects with marked deviations in serum chemistry, patients with serious underlying disease, including HIV infection, mal- nutrition or severe kala-azar, pregnancy or lactation, G6PD deficiency, or previous inclusion in this study.
Das-2005	All patients attending the outpatient department of our institution with fever, splenomegaly, Leishman-Donovan (LD) bodies in the bone marrow and/or splenic aspirate and without any history of treatment for kala-azar were included in the study.<br>Exclusion: Not reported
Ritmeijer-2006	Males aged >=15 years with parasitologically and/ or serologically confirmed VL attending Humera Hospital and Mycadra Health Center were enrolled in the study. Because of the potential teratogenicity of miltefosine, females were excluded. Previous antileishmanial treatment was recorded at hospital admission. Patients were enrolled in the study after giving informed consent. Potentially eligible patients were only excluded if they had such severe comorbidity that they were considered to be likely to die during the month’s treatment. Patients with previous antileishmanial treatment were only admitted if they had a positive aspirate result.

	The World Health Organization case definition of VL was used for initial screening: a history of fever for >2 weeks (with malaria excluded) in combination with wasting, and either splenomegaly or lymphadenopathy; A total of 580 adult male VL patients were enrolled in the study.
Sundar-2006	Patients were eligible for inclusion in the study if they had signs and symptoms of active VL (i.e., fever and splenomegaly) and if their splenic aspirate smear showed characteristic Leishmania amastigotes. Pregnant or lactating female subjects and patients who were found to be HIV positive by serological testing were excluded from the study. Patients who either were critically ill or had a concurrent serious infection/ illness also were excluded. Also excluded were patients who had a granulocyte count of <1000 granulocytes/mm3, a haemoglobin level of <3.5 g/dL, or a platelet count of<40,000 platelets/mm3; hepatic transaminase or total bilirubin levels that were 3 times the upper limit of the range considered to be normal; a serum creatinine level of 12.0 mg/dL, or a prothrombin time 15s above the control time.
Singh-2006	Children, who had received less than 30 days course of SAG, with bleeding diathesis, liver disorder (ALT & AST>3 times, serum bilirubin >2 times of normal), renal dysfunction (BUN and serum creatinine; 1.5 times of normal), co-existing malaria or HIV, neutrophil count less than 1000/cu mm and platelet count less than 40,000/cu mm were excluded from study.
Singh-2006	Children with bleeding diathesis, liver disorder (ALT & AST>3 times, serum bilirubin >2 times of normal), renal dysfunction (BUN and serum creatinine; 1.5 times of normal), co-existing malaria or HIV, neutrophil count less than 1000/cu mm and platelet count less than 40,000/cu mm were excluded from study. Patients who had received incomplete course of sodium stibogluconate (SAG) were also excluded from study.
Sundar-2007b	Pregnant or breast-feeding women, individuals who were seropositive for HIV, and individuals with a serious concurrent infection (e.g., tuberculosis or bacterial pneumonia) were excluded from the study. Exclusion criteria also included the following findings: a leukocyte count of <1000 cells/$mm^{3,}$ a haemoglobin concentration of<3.5 g/dL, or a platelet count of <40,000 platelets/mm3; hepatic transaminase or total bilirubin levels of >3 times the normal level; serum creatinine level, 12.0 mg/dL; and prothrombin time, >5 s greater than the control level.
Sundar-2007a	Inclusion criteria were parasitologically positive splenic or bone marrow smear; negative serologic testing for the human immunodeficiency virus (HIV); haemoglobin level of at least 5.0 g per decilitre; white blood count greater than or equal to 1×10exp9 per litre; platelet count greater than or equal to 50×10exp9 per litre; levels of aspartate aminotransferase, alanine aminotransferase, and alkaline phosphatase less than or equal to three times the upper limit of the normal range; prothrombin time less than or equal to 5 seconds greater than that among control subjects; and serum creatinine and potassium levels within the normal limits.<br>Exclusion criteria were treatment for visceral leishmaniasis during the 2 weeks before enrolment, a hearing loss of 75 dB in frequencies 1 through 8 kHz, a history of vestibular or auditory dysfunction, prior treatment with amphotericin without response, allergy or hypersensitivity to aminoglycosides, significant proteinuria (≥2+ on strip testing), significant coexisting diseases possibly affecting the response to the study treatment response, and pregnancy or lactation
Bhattacharya-2007	Eligible patients were of either sex between 2 and 65 years of age with a clinical diagnosis of active VL (e.g., fever or splenomegaly) and diagnosis confirmed by splenic aspirate showing characteristic amastigotes.<br>Exclusion criteria were ongoing pregnancy or breastfeeding, refusal to use contraceptive methods during the treatment period and 2 months thereafter (as advised by the clinical investigators), HIV-positive serology, or any condition or situation compromising with compliance to study procedures. Approximately 10% of patients did not fill eligibility criteria for inclusion in the study for various reasons (e.g., they had malaria; other causes of fever-like tuberculosis, typhoid fever, or urinary tract infection, etc.; or cirrhosis of liver or severe anaemia [haemoglobin level >=4 g/dL]). Patients with leukocyte count >1000/mm3 and haemoglobin level >=4 g/dL were included

Mueller-2007	Inclusion: Unresponsiveness to AmBisome in some Sudanese patients with kala-azar.<br>Exclusion: Not clear
Sundar-2008b	Patients >=12 years of age were eligible for the study if they had symptoms and signs of kala-azar (e.g., fever, weight loss, and splenomegaly) and parasites demonstrated by microscopic examination of splenic aspirate smear<br>Pregnant or breast-feeding women and individuals who were seropositive for HIV or who had a serious concurrent infection, such as tuberculosis or bacterial pneumonia, were excluded. Exclusion criteria also included granulocyte count !1000 cells/mL, haemoglobin level >3.5 g/dL or platelet count >40,000 platelets/mL, hepatic transaminase levels or total bilirubin >3 times the upper limit of normal, serum creatinine level >2.0 mg/dL, and prothrombin time 15 sec above control. If they were randomized to receive Miltefosine, women of child-bearing age with negative pregnancy test results were counselled about the potential teratogenic effects of Miltefosine and were offered contraception in the form of a depot preparation of progesterone. All such women enrolled in this study gave consent and received the injection
Sundar-2008a	Patients of either sex aged 12–65 years (both inclusive) with signs and symptoms of VL (fever, weight loss and splenomegaly), and demonstrable parasite in splenic smears were included in this study. Patients who had failed to respond to sodium stibogluconate treatment previously were also included after a washout period of 10 days<br><br>Patients were excluded if they were breast feeding or pregnant, if they were HIV positive or if they had previously failed treatment with amphotericin B for VL. Additional exclusion criteria were a granulocyte count of <1000 mm)3; a haemoglobin level <6 gm ⁄dl or a platelet count <50 000 mm)3; hepatic transaminases >3 times the normal upper limit and ⁄or total bilirubin >2 times of normal upper limit; serum creatinine ‡ 1.5 times of normal upper limit; or a prothrombin time >5 s above the control range. Patients with concomitant life-threatening disease or serious concurrent infection such as tuberculosis or bacterial pneumonia and those allergic to amphotericin B or any other ingredients of the emulsion formulation were also excluded from the study
Thakur-2008	Patients were excluded if they had a haemoglobin concentration below 50g/dl, had complications such as pneumonia, jaundice, tuberculosis, renal and cardiac diseases, or had received treatment with antimony or amphotericin B (AMB) or any treatment for kala-azar or refused to be included in the study. Patients with parasitological infections with hookworm, roundworm and Entamoeba histolytica were not excluded and these infections were treated concurrently
Mueller-2008	Twenty-five (11.9%) patients with malaria, 22 (10.5%) with respiratory tract infections, and 21 (10.0%) with other bacterial infections (such as typhoid fever, otitis media, pharyngitis, brucellosis or dysentery) were given specific treatments for their accompanying infections

Sundar-2009a	Patients of either sex and 18–65 years of age (both inclusive) with signs and symptoms of VL (fever,<br>weight loss, and splenomegaly) who had demonstrable parasites in splenic smears were included in this study. Patients<br>who had failed treatment with antileishmanial drugs other than amphotericin B were also included after completion of a<br>washout period of 10 days.<br><br>Patients were excluded if they were breast feeding or pregnant, and had positive serologic results for infection with human immunodeficiency virus (HIV). Exclusion criterion also included a granulocyte count < 1,000 cells/mm 3; a haemoglobin level < 6 g/dL, a platelet count < 50,000 cells/mm 3; levels of hepatic aminotransferases> 2.5 times the upper limits of normal levels, total bilirubin levels > 1.5 times the upper limit of normal ranges; pro- thrombin time > 5 seconds than that of controls, and serum creatinine levels ≥ 1.5 times the upper limit of normal ranges. Patients with concomitant life-threatening disease or serious concurrent infection such as malaria, tuberculosis, or bacterial pneumonia, and those allergic to amphotericin B or any other ingredients in the emulsion formulation were also excluded from the study
Das-2009	Patients of either sex aged between six to 60 years unresponsive to SSG after a full course of treatment and having com- plaints of continued fever with rigor, hepato-splenomegaly, anorexia, anaemia, and loss of weight, sputum for acid-fast bacilli (AFB) and chest x-ray (PA-view) negative for tuberculosis or pneumonia, and demonstration of parasite in bone marrow (BM)/splenic(SPL) aspiration were the inclusion criteria.<br><br>The confirmed VL cases with partial dosages of SSG and with associated diseases like tuberculosis (TB), HIV/AIDS, and malaria, pregnant women, lactating mother, and diabetics were not included in the study. The individuals who had past history of Kala-azar in last five years and having Hb< 6 g/dl, platelet count <50,000/mm3 with significant abnormalities in liver and renal function tests were the exclusion criteria.
Sundar-2009b	Patients of either sex who were 5–55 years of age with signs and symptoms of VL (i.e., fever, weight loss, and splenomegaly) who had demonstrable parasites in splenic smears were included in this study. Patients with relapsed VL or who experienced treatment failure while receiving a regimen that did not contain paromomycin were also included.<br>Women with child-bearing potential were excluded from the study if they were pregnant, or were not using any contraception. Exclusion criterion included a granulocyte count of less than 1000 cells/mm3, a haemoglobin level of less than 4 g/dL, a platelet count less than 40,000 platelets/ mm3, liver function test results greater than 3 times of upper limit of normal, normal, prothrombin time 5 sec more than that for control subjects, serum creatinine level above the upper limit of normal, abnormal audiometric, and/or vestibular function, serological test results positive for HIV, and concomitant severe medical conditions. Patients who were allergic to aminoglycosides, those treated with a parenteral aminoglycoside within 28 days prior to randomization or with paromomycin at any time and those who had taken VL treatment within the past 14 days were excluded.
Adam-2009	Pregnant women with symptoms and signs suggestive of VL, such as fever, weight loss, anaemia, and hepatosplenomegaly, were approached to participate in the study. Besides being pregnant, the inclusion criteria were confirmation of Leishmania infection by the presence of amastigotes in Giemsa-stained smears of bone marrow, and signing an informed written consent form. None of the patients had malaria or HIV
Shahian-2009	No patient had immunosuppression caused by HIV infection or renal transplantation and none of them had received medications inducing pancreatitis or prior antileishmanial therapy
Hailu-2010	Patients were excluded if they: (1) had taken any antileishmanial drug in the preceding 6 months; (2) showed severe protein or caloric malnutrition (Kwashiorkor or marasmus); (3) had previous hypersensitivity reaction to SSG or aminoglycosides; (4) had a concomitant severe infection (except HIV) or any other serious underlying (cardiac, renal, hepatic) disease; (5) had conditions associated with splenomegaly, such as schistosomiasis; (6) had a history of cardiac arrhythmia or an abnormal electrocardiogram (ECG); (7) were pregnant or lactating; (8) had any relevant outliers of safety

	laboratory parameters (haemoglobin ,5 g/dL, white blood cells ,16103/mm3, platelets ,40,000/mm3, liver function test values .3 times higher than upper limit of normal, serum creatinine values above upper limit of normal); or (9) had clinical hearing loss. HIV infection was not an exclusion criterion.<br>Eligible patients were 4-60 years old and had to have clinical symptoms (fever and splenomegaly) and a diagnosis of VL, confirmed by microscopic verification of parasites in spleen, lymph node or bone marrow tissue aspirates.
Thakur-2010	Exclusion criteria included patients who had tuberculosis, infection with human immunodeficiency virus, acquired immunodeficiency syndrome, kidney and heart disease, and leukocyte counts < 1,000 cells/µL, a haemoglobin concentration < 5 g/dL, serum aspartate aminotransferase and alanine aminotransferase levels > 3 times the upper limit of the reference range, thrombocyte counts < 60,000 cells/µL, or serum creatinine levels > 1.5 times the upper limit of the reference range. If patients had haemoglobin levels < 5 g/dL or a thrombocyte count < 65,000 cells/µL, whole blood transfusions or platelet transfusions were given, respectively. If these two parameters reached acceptable levels, only then were the patients included in the trial.
Musa-2010	Patients were excluded from the study if they: had negative bone-marrow smears; were clinically contraindicated to having a bone-marrow aspirate; received any anti-leishmania drug in the past 6 months; had severe protein or caloric malnutrition (Kwashiorkor or marasmus); had previous hypersensitivity reaction to aminoglycosides; suffered from a concomitant severe infection, i.e. tuberculosis, HIV, or any other serious underlying disease (cardiac, renal, hepatic); suffered from other conditions associated with splenomegaly such as schistosomiasis; had previous history of cardiac arrhythmia or an abnormal electrocardiogram (ECG); were pregnant or lactating; or had pre-existing clinical hearing loss. If tuberculosis or schistosomiasis were suspected, these were screened through laboratory testing. Additionally, patients with the following laboratory values were excluded: haemoglobin less than 5 g/dL; white blood cell less than 103/mm3; platelets less than 40,000/mm3; liver function test values more than three times the normal range; and serum creatinine values outside the normal range for age and gender.
Sundar-2010	Patients between the ages of 2 and 65 years were eligible if they had symptoms and signs of leishmaniasis (e.g., fever, weight loss, and splenomegaly) and if parasites were shown on microscopy of a splenic aspirate smear.<br>Patients who were seropositive for the human immunodeficiency virus (HIV) or who had a serious concurrent infection, such as tuberculosis or bacterial pneumonia, were excluded. Exclusion criteria also included a white-cell count of less than 750 per cubic millimetre, a haemoglobin level of less than 3.5 g per decilitre, and a platelet count under 40,000 per cubic millimetre; levels of hepatic aspartate aminotransferase or total bilirubin of more than five times the normal range; a serum creatinine level of more than 1.5 times the upper limit of the normal range; and a prothrombin time of more than 4 seconds above the control level.

Mondal-2010	Based on the inclusion exclusion criteria (Protocol S1): patients of all ages and both sexes were potentially eligible if they presented the clinical symptoms of prolonged fever, hepatosplenomegaly, and were confirmed to be VL by K39 strip test and detection of Leishmania parasites in the splenic or bone marrow aspirate.<br>- Patients of all ages and both sexes<br>-Consistent signs and symptoms of active VL.<br>-Confirmed diagnosis of VL with positive identification of parasite from bone marrow or splenic aspirate or K39 strip test.<br>-Confirmed VL diagnosis patients fully informed consent (self or parent or authorized relative) of their willingness to undergo treatment with the new drug (Fungisome) at the specific dose.<br>-Patients willing to participate in all treatment and follow-up visits regularly at monthly intervals for six months at the out-patient department at the school of Tropical Medicine<br><br>Excluded:<br>-HIV-positive individuals with VL<br>-VL Patients already receiving antileishmanial drugs will be excluded from the study.<br>-Patients who have hypersensitivity to the drug or its constituents.<br>-Patients who have associated disease altering liver function tests.<br>-Pregnant women.
Rijal-2010	Parasitologically proven kala-azar cases who were treatment-naïve to Sbv therapy were enrolled in the study after obtaining informed consent.<br>Exclusion: Not presented
Singh-2010	Children with bleeding diathesis, liver disorder [alanine aminotransferase (ALT) and aspartate aminotransferase (AST) >3 times, serum bilirubin >2 times of normal], renal dysfunction [Blood urea nitrogen (BUN) and serum creatinine, 1.5 times of normal], co-existing malaria or HIV, neutrophil count <1000mm-3 and platelet count less than 40 000mm-3 were excluded from study. Patients who had received incomplete course of SAG were also excluded from study.
Sinha-2010	Inclusion: (1) clinical symptoms consistent with kala-azar, (2) ≥ 2 years of age, (3) residence within Vaishali District, (4) provision of written informed consent, and (5) a positive rK39 rapid diagnostic test (DiaMed-IT-Leish; in northeast India, rk39 has shown 99% sensitivity and 100% specificity with whole-blood samples) or a parasitological diagnostic test result.<br><br>Exclusion: Patients excluded from the study were those with (1) post-kala-azar dermal leishmaniasis, (2) prior treatment of current kala-azar infection with ≥ 20 mg/kg of liposomal amphotericin B, (3) known allergic reaction to amphotericin B, or (4) human immunodeficiency virus, tuberculosis, or malaria co-infections.<br>Pregnancy was noted for 3 of 46 (6.5%) women of childbearing age.
Sundar-2011b	Reasons for exclusion were haemoglobin concentrations less than 50 g/L, total leucocyte count less than 1×10^9/L, serum creatinine concentration outside the normal range of 55–140 µmol/L, platelet count less than 40×10^9/L, serum aminotransferase concentration higher than three times the upper limit of the normal ranges, bilirubin concentration more than 34·2 µmol/L, prothrombin time more than 5 s longer than control, positive

	serology for HIV or hepatitis B or C viruses, severe concurrent illness, and receipt of any antileishmanial or antifungal drug in the previous 45 days. Pregnant and breast-feeding women, patients with known hypersensitivity to the study drugs, and those with diabetes, hypertension, or tuberculosis were also excluded.
Sundar-2011c	Eligible patients were men or women 16−50 years of age with VL symptoms or signs and confirmed VL<br><br>Exclusion criteria were renal, hepatic or biliary disease; renal or hepatic impairment; cardiac disease, arrhythmia, or conduction abnormalities; clinically relevant electrocardiogram (ECG) results or laboratory values; serious underlying disease or infection; glucose-6-phosphate dehydrogenase deficiency (based on phenotype testing); positive results for antibodies against human immunodeficiency virus, hepatitis B surface antigen, or antibodies to hepatitis C virus; contraindication to splenic or bone marrow aspiration; hypersensitivity to study treatments; treatment with an established anti-leishmania drug within 30 days or 5 half-lives (whichever was longer) of the start of the study; or treatment with prohibited medication. Pregnant or nursing women were excluded; a negative urine pregnancy test result was required from female patients at screening and before dosing, plus an agreement to use contraception for two weeks.
Sundar-2011a	Patients of age 2–65 yrs. with mild-moderate kala-azar; lack of concomitant diseases such as malaria, HIV, and tuberculosis; lack of pregnancy or lactation; no previous treatment with antimony or paromomycin unless the treatment terminated two months prior to the study and the patient was worsening.
Rahman-2011	Patients were 2–65 years of age. Both male and female patients participated, and patents were enrolled during October 2006–September 2007. Inclusion criteria were signs and symptoms compatible with VL (fever for at least two weeks, palpable splenomegaly, weight loss by history), positive serologic test result for leishmaniasis (rK39), haemoglobin level ≥ 6 g/dL, no infection with HIV, not pregnant or lactating, not being currently treated with an antileishmanial compound, and no significant concomitant medical condition. Malaria and enteric fever were ruled out by blood smears and the Widal test, respectively. If present, malaria and enteric fever were treated. These patients could then be admitted into the study. If tuberculosis was suspected on clinical grounds, the patient was not eligible for this study.
Sinha-2011	Exclusion criteria included concurrent illness such as HIV, malaria, and tuberculosis; a history of hearing loss that could confound clinical detection of potential ototoxicity; recent or current exposure to medications that could result in com- pounded toxicities. Before study enrolment, informed consent was obtained from every patient or a legally authorized representative
Sudarshan-2011	Forty-six patients with parasitologically confirmed VL (6–55 years, males and females, and HIV negative) were enrolled in the study. Confirmation of VL was done by demonstration of amastigotes in Giemsa-stained smears of splenic aspirate.
Sundar-2012	Criteria for exclusion were current pregnancy; current breast-feeding; seropositivity for human immunodeficiency virus; presence of a serious illness or concurrent infectious disease, such as tuberculosis or bacterial pneumonia; granulocyte count <1000 granulocytes/mm3; haemoglobin level <5.0 g/dL; platelet count <40 000 platelets/mm3; hepatic transaminase levels >5 the normal level; total bilirubin level >2.0 mg/dL; serum creatinine level above the upper normal limit; prothrombin time >5 seconds above control; and/or inability of the subject or guardian to provide written informed consent.<br>Because of the long half-life of Miltefosine and its known teratogenic potential, women of childbearing age were asked to practice contraception during treatment and for an additional 3 months after completion of treatment. Treatment was directly observed by a clinic nurse, who observed the swallowing of the pills.

Musa-2012	Briefly, patients aged 4–60 years with parasitologically confirmed VL were included, but patients with very severe VL or those with contraindications were excluded. Being HIV-positive was not an exclusion criterion but the original protocol stated that there was to be a sufficient number of patients for a subgroup analysis excluding HIV patients (if deemed necessary). Patients were excluded if they: (1) had taken any antileishmanial drug in the preceding 6 months; (2) showed severe protein or caloric malnutrition (Kwashiorkor or marasmus); (3) had previous hypersensitivity reaction to SSG or aminoglycosides; (4) had a concomitant severe infection (except HIV) or any other serious underlying (cardiac, renal, hepatic) disease; (5) had conditions associated with splenomegaly, such as schistosomiasis; (6) had a history of cardiac arrhythmia or an abnormal electrocardiogram (ECG); (7) were pregnant or lactating; (8) had any relevant outliers of safety laboratory parameters (haemoglobin <5 g/dL, white blood cells <1103/mm3, platelets <40,000/mm3, liver function test values >3 times higher than upper limit of normal, serum creatinine values above upper limit of normal); or (9) had clinical hearing loss.
Patra-2012	Patients with following were excluded from the study: platelet count < 50,000 /mm 3; total leukocyte count < 1000 /mm 3; haemoglobin concentration < 6 g/dL; bilirubin more than twice the upper limit of normal and serum creatinine or blood urea nitrogen levels of 1.5 times the upper limit of normal. No patients had been treated previously for VL. Cases with other serious concurrent medical illness, HIV seropositivity, and pregnancy were also excluded.
Rijal-2013	Exclusion criteria included pregnancy or breast feeding, as well as women of child-bearing age who refuse or are unable to maintain contraception for a period of two months after completion of treatment. Also, SGPT/SGOT > 3 times, serum bilirubin > 2 times and serum creatinine> 1 5 times the upper limit of normal values, and cases with severe anaemia or known kidney or liver disease were excluded. The patients that did not match the inclusion criteria were treated with amphotericin B (1 mg/kg/day for 14 days). MIL relapse cases were also not included.
Khalil-2014	Patients with age of at least 4 years, confirmed HIV-negative, parasitologically-confirmed non-severe VL, were enrolled in three centres .<br>Exclusion criteria were signs/symptoms of severe VL (patients who were very weak, unable to walk, bleeding, jaundiced, suffering from sepsis and other concomitant infections/illnesses); anti-leishmanial or unlicensed investigational<br>treatments within six months; underlying chronic disease such as severe cardiac, pulmonary, renal, or hepatic impairment; serum creatinine outside the normal range; liver function tests more than 3 times the normal range; platelet count less than 40,000/mm3; known alcohol abuse; pregnancy or lactation; concomitant acute drug usage for malaria and bacterial infection; pneumonia within last 7 days; known hypersensitivity to AmBisome or amphotericin B; any other condition which may invalidate the trial
Ostyn-2014	No exclusion criteria reported. In our study area HIV co-infection is rare, HIV is tested for and when positive, patients are treated with amphotericin B, so our results on Miltefosine-treatment concern an HIV-negative population.
Cota-2014	No information.
Sundar-2014	Other inclusion criteria were haemoglobin (Hb) >=5 g/dL, white blood cells count >=1000/cmm, platelet count >=50000/cmm, prothrombin time <=4 seconds above the control, and alkaline transaminase, aspartate transaminase, and alkaline phosphatase <=2.5 times the upper limit of normal. Patients with past history of treatment with AmB or any other drug for VL within 30 days prior to screening, major surgery within 2 weeks prior to screening, concurrent malaria, alcoholism or illicit drug use/abuse or any condition associated with poor compliance, hypersensitivity to AmB, inactive ingredients of ABLE and LAmB formulations were excluded from the study. Patients who received any of the prohibited medications (any other investigational drugs, antileishmanial drugs other than study drug, corticosteroids, skeletal muscle relaxants, cyclosporine, digoxin, vancomycin, aminoglycosides, antifungal, immunosuppressive agents, and all potentially nephrotoxic drugs), who were positive for human

	immunodeficiency virus, hepatitis C virus and hepatitis B surface antigen infections and immune-compromised, were also excluded from the study. The following is from study protocol (supplemental file): Inclusion: Non-pregnant, non-lactating females of age ≥5 years, and woman of childbearing potential (any woman who has reached menarche) who are willing to use acceptable methods of contraception like long term acting injections ex. Depo-Provera.
Mondal-2014	We excluded participants with a history of intercurrent or presence of clinical signs or symptoms of uncontrolled concurrent diseases or conditions before start of study treatment, any condition that might prevent the patient from completing the study therapy and subsequent follow-up (investigator assessed), a history of allergy or hypersensitivity to amphotericin B, previous treatment for visceral leishmaniasis within 2 months of enrolment, previous treatment failure with amphotericin B, post-kala-azar dermal leishmaniasis, and pregnant women. We excluded pregnant women from this study because present evidence about safety of liposomal amphotericin B during pregnancy is limited.
Jamil-2015	Exclusion criteria included pregnancy or lactation; active tuberculosis or taking antituberculotic medications; previous treatment with PMIM; clinically significant anaemia; current or history of clinically significant renal or hepatic dysfunction; serum creatinine above the upper limit of normal range; proteinuria; history of hepatitis B or C or HIV positive; history of hearing loss; significant coexisting disease; any history of VL or treatment for VL; history of hypersensitivity to aminoglycosides or sulphite; and concomitant use of other aminoglycosides, nephrotoxic and ototoxic drugs, or immunosuppressive drugs.
Sundar-2015	Patients were excluded in the study if they had haemoglobin of < 5 g/dL, serum creatinine or blood urea nitrogen (BUN) > 1.5 times the upper limit of normal, a platelet count < 40,000/m3, serum bilirubin > 2 mg/dL, prothrombin time of more than 5 seconds above the control levels, history of VL treatment in the last 45 days, hepatitis B or C, human immunodeficiency virus (HIV), active tuberculosis or another serious illness or associated disease known to alter liver/kidney functions, or known hypersensitivity/allergy to the study drug or their constituents or were women who were pregnant or lactating.
Goswami-2016	120 treatment naïve non-HIV; The inclusion criteria were as follows: all patients with symptoms and signs suggestive of VL (fever for more than 2 weeks and splenomegaly) with rK39 immunochromatographic strip test (Kalazar Detect™; InBios International Inc., Seattle, WA) positivity and demonstrable amastigotes of Leishmania donovani parasites in splenic aspirates between 2008 and 2011.<br>The following patients were excluded from the study: pregnant and lactating women, human immunodeficiency virus-positive individuals, those previously treated with or receiving concomitant antileishmanial drugs, patients with platelet count below 50,000/?L, white blood cell count below 1,000/?L, serum aspartate aminotransferase and alanine aminotransferase levels more than three times the upper limit of normal, serum creatinine or urea values more than 1.5 times the upper limit of normal, and patients with other major medical illness such as ischemic heart disease, congestive cardiac failure, and stroke.
Wasunna-2016	Eligible patients were HIV negative, and aged between 7 and 60 years with parasitologically confirmed VL who signed an informed consent (if aged 18y and over) or whom the parent or legal guardian consented to participate in the study (if under 18y). The target population was primary cases, so known relapse cases, or receipt of any anti-leishmanial drugs in the previous 6 months, was an exclusion criterion. Other exclusion criteria were: severe protein and/or caloric malnutrition defined as kwashiorkor or marasmus in children and BMI <15 in adults; previous history of hypersensitivity reaction to SSG or amphotericin B; concomitant severe infection such as TB or other serious underlying disease which would preclude evaluation of patients response to the study medication; other conditions associated with splenomegaly such as schistosomiasis; previous history of cardiac arrhythmia or an abnormal ECG; Hb<5 g/dL; WBC <103/mm3; platelets <40,000/mm3, abnormal liver function tests (ALT and AST) of more than three times the upper limit of the normal range, serum creatinine outside the normal range for age and gender, and major surgical intervention within two weeks prior to enrolment. Due to the potential teratogenicity of Miltefosine, females of child bearing age were also excluded.

Pandey-2016	Females and males between 6-70 years of age having signs and symptoms suggestive of VL (fever of 2 weeks with splenomegaly, not responding to antimalarials and antibiotics) and diagnosed for VL infection as per the prevailing guidelines of VL diagnosis in national kala-azar elimination program were included in this study.<br>Criteria for exclusion were pregnant/breast-feeding women, women of child-bearing age not consenting to avoid pregnancy during and after 6 months of treatment, seropositive for HIV, presence of a serious illness, concurrent diseases, such as tuberculosis or bacterial pneumonia, haemoglobin level < 5.0 g/dL, granulocyte count < 1,000 granulocytes/mm3, platelet count < 40,000 platelets/mm3, hepatic transaminase levels > 5 times of the normal limit, total bilirubin level > 2.0 mg/dL, serum creatinine level above the upper normal limit (> 1.5 mg/dL), prothrombin time > 5 seconds above control, and/or inability of the subject or guardian to provide written informed consent.
Borges-2017	Eligible patients were children aged 6 months-12 years who met the inclusion criteria for VL diagnosis (presence of fever for at least two weeks associated with splenomegaly). Participation in this study was voluntary upon the signature of the informed consent form by the patients' parents or legal guardians. Treatment naïve children and adolescents with VL without signs of severe illness were treated.<br>Exclusion criteria were the following: patients who underwent previous treatment with leishmanicidal drugs, clinically evident jaundice (total bilirubin > 2.5mg/dL), haemorrhages with coagulation disorders, generalized oedema, signs of toxaemia, severe malnutrition according to Gómez criteria9, presence of comorbidities or immunosuppressive conditions, and lack of informed consent.
Rahman-2017	HIV negative primary VL patients between 5 and 60 years screened with positive rK39 rapid immunochromatographic tests (InBios, Seattle, USA) and parasitologically confirmed via bone marrow or spleen aspirates (only at CBMC) were enrolled into the study after giving informed consent.<br><br>Because of the potential teratogenic effects of Miltefosine, women of childbearing age who were not using an assured method of contraception for the duration of treatment and three months afterwards were excluded, unless they agreed to receive an injection of medroxyprogesterone acetate (DepoProvera, Pfizer, NY, USA). One injection (which is effective for 3 months) was needed to ensure adequate coverage, taking into account Miltefosine long half-life of approximately 7 days. Single women of childbearing age were randomized to receive either AmBisome alone or AmBisome + paromomycin.<br><br>Other exclusion criteria were: known hepatitis B, hepatitis C, or HIV infection, Hb concentrations less than 5 g/dl, platelet count of less than 40,000/mm3 (at CBMC only), a prothrombin time of more than 5 seconds longer than the control (at CBMC only), severe malnutrition [for adults (> 18 years) defined as BMI <14; for children (< 18 years) defined as BMI for age z score < -3 in children measuring > 121.5cm; and weight for height less than 60% in children measuring <121.5 cm], known alcohol or drug abuse, use of any investigational (unlicensed) drug within the last 3 months, and severe concurrent illnesses (TB, malaria) or chronic conditions (diabetes, hypertension). Pregnant and breast-feeding women, and patients with known hypersensitivity to the study drugs were also excluded.

Romero-2017	Exclusion criteria were pregnancy; HIV infection; underlying chronic or acute disease which would preclude evaluation of the participant's response to study medication (e.g. diabetes, cardiac, renal, or hepatic impairment, schistosomiasis, malaria, tuberculosis); co-morbidities that may cause alterations of the immune system; any concomitant use of medication that may interfere with the therapeutic response or cause detrimental pharmacological interactions; previous treatment with any anti-leishmanial drugs<br>in the past 6 months; drug abuse; previous history of hypersensitivity reaction to tested interventions; any condition that may hinder compliance with the planned scheduled visits; relapse cases; clinical signs of severe VL disease, such as generalized edema, jaundice, toxemic signs, and severe malnutrition; serum creatinine and bilirubin above the upper normal limit (UNL); prolonged prothrombin time with international normalized ratio (INR) > 2.0; or a platelet count < 20,000/mm.
Alborzi-2017	Inclusion criteria were signs of infection (fever, splenomegaly, etc.), positive serology IFA titer 1/128 or rK39 strip test, and/or positive bone marrow microscopy for Leishman bodies. Exclusion criteria were presence of clinically obvious jaundice, disseminated intravascular coagulation (DIC), and/or shock.
Pandey-2017	Participants of either sex, aged less than 15 years, and having clinical signs and symptoms consistent with kala-azar and positive rK39 test (Inbios) were included. The diagnosis was confirmed by parasitological analysis of splenic or bone marrow aspiration. Only parasitologically confirmed cases were included in the study. Fourteen patients had a past history of VL for which they had been treated with different antileishmanial drugs (Table 1)<br><br>Exclusion criteria were haemoglobin (Hb) < 5 g/100 mL; white blood cells count < 1,000/mm3; thrombocyte count < 50,000/mm3; alanine transaminase, aspartate transaminase, and alkaline phosphatase > 2.5 times upper limit of normal range; bilirubin ³ 2 times upper limit of normal, serum creatinine or blood urea nitrogen ³ 1.5 times upper limit of normal. Patients seropositive for HIV, hepatitis B and C, hypersensitivity to amphotericin B or inactive ingredients of the amphotericin B formulation were also excluded.
Kimutai-2017	Only consenting patients treated with a SSG-PM combination were enrolled. Patients did not receive the combination<br>if they had severe renal, cardiac or other systemic disease based on clinician judgment, or if they were known<br>to be taking an aminoglycoside or were hypersensitive to either SSG or PM. As per MSF clinical protocol, HIV coinfected<br>patients and patients above 50 years of age received AmBisome rather than SSG-PM in MSF sites. In other sites, those patients who received SSG-PM were thus included in the PV programme.
Mbui-2018	Each recruited patient fulfilled the following inclusion criteria: (i) clinical signs and symptoms of VL and confirmatory parasitological microscopic diagnosis, (ii) aged ≥4 years and ≤12 years, (iii) able to comply with the study protocol, (iv) written informed consent signed by parents(s) or legal guardian, and children`s assent (>8-12 years, in Uganda only), (v) body weight of <30 kg. None suffered severe malnutrition or any serious underlying disease or concomitant severe infection. None had received anti-leishmanial drugs within the previous 6 months.<br><br>From Supplementary file: They each had none of the following exclusion criteria: (i) relapsed VL, (ii) received any anti-leishmanial drugs within the previous six months, (iii) severe malnutrition (= a z score of <-3 on weight-for-height WHO reference curves, if aged <5 years or a z score of <-3 on BMI-for-age WHO reference curves, if aged 5-12 years), (iv) positive HIV diagnosis, (v) previous history of hypersensitivity to miltefosine, (vi) concomitant severe infection or any other serious underlying disease, (vii) any condition associated with splenomegaly, such as schistosomiasis, (viii) had reached menarche (female patients), (ix) haemoglobin concentrations of <5 g/dL, (x) white blood cell count of <1x103/mL blood, (xi) platelet count of <40,000/mL blood, (xii) abnormal liver function (= blood alanine-transferase and aspartate transferase levels >3-times above upper limit of

	normal range), (xiii) blood bilirubin levels >1.5-times greater than upper limit of normal range, (xiv) serum creatinine levels above upper limit of normal range, (xv) clinical signs of severe VL (jaundice and bleeding), (xvi) unable to comply with study protocol.
Goyal-2018	All patients meeting a case definition of VL defined as fever for more than 2 weeks, splenomegaly, and confirmed with a positive rK-39 rapid diagnostic test (InBios, USA) were included in the study. Relapse cases with a confirmatory parasitological diagnosis were also eligible. Patients with concurrent PKDL, HIV and those reporting a history of hypersensitivity to the investigational drugs were excluded. Patients with haemoglobin <4 g/dl, serious concomitant infection (e.g. severe pneumonia), complicated severe malnutrition, TB/VL co-infection, or children <2 years of age were referred to the MSF VL treatment unit within Hajipur district hospital or RMRIMS for further specialist management. These patients were treated with SDA as per physician decision and included in the study.
Sundar-2019	Patients of all ages and of both genders, having signs and symptoms suggestive of VL, that is, fever<br>with chills and rigor for 2 weeks or more, not responding to antimalarial drugs, and splenomegaly were included in the study. An rK39 antigen–based immunochromatographic serological rapid test was performed. Patients with typical signs and symptoms with characteristic laboratory features (pancytopenia) and a positive strip test were included in this study. Patients with serious illnesses or concurrent infections, such as tuberculosis or bacterial pneumonia, HIV, and Hepatitis B/C, and known allergy to AmB were excluded.
Diro-2019	• Confirmed HIV positive test (2 rapid diagnostics tests (RDTs) followed by a confirmatory ELISA test).<br>• Diagnosis of VL (first episode or relapse) confirmed by bone marrow or spleen aspirate.*<br>• Male and female age: 18-60 years.<br>• Written informed consent from the patient.<br><br>(NOTE: information in supp. file)<br><br>The presence of any of the following will exclude a patient from study enrolment:<br>• Women of child-bearing potential (defined as women who have achieved menarche) who are not using an assured method of contraception or are unwilling to use an assured method of contraception for the duration of treatment and four months after.***<br>• Pregnant women or breast-feeding mothers.<br>• Patients with grade 2 or 3 post kala-azar dermal leishmaniasis (PKDL) lesions.<br>• Clinical or biological evidence of severe cardiac, renal or hepatic impairment.<br>• Known hypersensitivity to AmBisome® and/or miltefosine.<br>• Patients receiving allopurinol treatment

Sinha-2019	We included children, 2-11 years of age of both male and female sex, and had VL suspected on the basis of clinical presentation (fever, splenomegaly determined by palpation, anaemia, and cytopenia) and confirmed by the demonstration of amastigotes in splenic aspirate specimens.<br><br>Exclusion criteria were values for the formed elements of the blood suggesting a premorbid state and severe disease (platelet count, <50,000 platelets/µL; leukocyte count, <1000 leukocytes/µL; haemoglobin concentration, <6 g/dL; serum aspartate aminotransferase (ASAT) or alanine aminotransferase levels of >3 times the upper limit of normal; bilirubin level of more than twice the upper limit of normal; and serum creatinine or blood urea nitrogen levels of >1.5 times the upper limit of normal), documentation of other serious coincidental medical illnesses, HIV seropositivity, and, for patients capable of reproduction, pregnancy.
Goswami-2020	Patients of both genders aged between 5 and 65 years with a corroborative clinical history (patients from endemic areas with a prolonged fever not responding to antimalarials antibiotics), physical signs (anaemia, splenomegaly, and hepatomegaly), and presence of parasites confirmed by examination of Giemsa-stained slides of splenic or bone marrow aspirates were enrolled into the study. The following groups of patients were excluded: HIV-positive individuals, infants and children with body weight < 10 Kgs, patients with severe concurrent illnesses, patients who received any antileishmanial drugs or antifungal drugs in the previous 45 days, pregnant patients, and those with withdrawal of contraceptive measures. We also excluded patients with known hypersensitivity to the study drugs and those with diabetes, hypertension, tuberculosis, and heart, liver, or kidney disease.
Ekram-2021	After taking consent either from the patients or parents (or guardians) of the child, all consecutive adult and paediatric cases (age range 3-65 years) suspected of VL were approached for inclusion into the study. During final inclusion, parasites found on microscopy of a splenic aspirate smear or bone marrow was considered the prime selection criteria Included: patients who were seropositive for HIV, and had a serious concurrent infection (e.g. TB or bacterial pneumonia) (Zahra et al. 2018), a serum creatinine level of [1.5 times the upper limit of the normal range, or have a history of allergy or hypersensitivity to amphotericin B. Moreover, anti-leishmanial or unlicensed investigational treatments within 180 days, pregnant women and patient and/or attendant refusing to give consent to take part in the study were also excluded.
Haidar-2001	The inclusion criteria were: all the confirmed cases of visceral leishmaniasis up to 12 years of age with an agreement from the parents or relatives for follow-up. History of taking antimalarial drugs was positive in 47%.

Supplemental table 4: Methodology adopted for assessment of nutritional status (n=21 studies)

Author year	Inclusion of malnourished patients as part of eligibility criteria	Was malnutrition defined?	Definition of malnutrition	Indicators for assessing nutritional status
Ritmeijer-2006	Included [a]	Not explicitly defined	Malnutrition is not explicitly defined but nutritional status of the patients was measured based on BMI (as reported in Table 2 of the manuscript)	Anthropometric indicators
Wasunna-2005	Excluded	Not reported	Not reported	Not reported
Jha-2005	Excluded	Not reported	Not reported	Not reported
Hailu-2010	Included	Yes	Patients who showed severe protein or caloric malnutrition (Kwashiorkor or marasmus) were exclusion.<br><br>From the footnote on Table 1 of the article, the following definition of nutritional status is presented:<br><br><u>Children</u>: Weight for age defined as severely underweight if <60%, underweight if between 60% and 80%, normal if >80%.<br><u>Adults</u>: BMI defined as severely underweight if <16, underweight if between 16.0 and 18.4, normal if between 18.5 and 24.9	Anthropometric indicators
Musa-2010	Excluded	Yes	Patients who had severe protein or caloric malnutrition (Kwashiorkor or marasmus) were excluded.<br><br>From the footnote on Table 1 of the article, the following definition of nutritional status is presented:<br><br>Based on Weight for Height (WHO child growth standards) if age <6 years and BMI for age if age 6–19 years; defined as severely underweight if z-score < -3SD; underweight if -3 SD ≤ z-score < -2SD; normal if -2SD ≤ z-score < +1SD; and BMI if age >19 years: defined as severely underweight if <16, underweight: 16.0–18.4, normal: 18.5–24.9	Anthropometric indicators
Dietze-2001	Excluded	Yes	Severe protein and/or caloric malnutrition (kwashiorkor or marasmus)	Clinical definition

Mueller-2008	Included	Yes	Categorization of nutritional status was based either on weight-for-height, for girls who were <137 cm tall and boys who were <145 cm tall, or on body mass indexes (BMI).<br><br>Weights-for-height that were <70%, 70%–79%, 80%–89% and >89% of 'normal' (Anon. 1977) or BMI of <16, 16–17, >17–18, and >18 were taken as indications of severe, moderate, mild, and no malnutrition, respectively.	Anthropometric indicators
Khalil-2014	Included	Yes	From the footnote on Table 1 of the article, the following definition of nutritional status is presented:<br><br>Classified using weight for height and BMI for age in those aged ≤19 years and BMI in those aged >19: normal if -2SD ≤ weight for height or BMI for age <+1SD or 18.5 ≤ BMI <25.0; underweight if -3SD ≤ weight for height or BMI for age <-2SD or 16.0 ≤ BMI <18.5; severely underweight if weight for height or BMI for age <-3SD or BMI<16.0.	Anthropometric indicators
Musa-2012	Included	Yes	Nutritional status was classified as normal, underweight, or severely underweight according to WHO Child Growth Standards in those <19 years and body mass index (BMI) in those ≥20 years.	Anthropometric indicators
Veeken-2000	Included	Yes	Body mass index (BMI) (calculated for patients aged 16 years or older), weight-for-height (calculated for patients aged under 16 years). The patients had a range of disease severity, but many were in an advanced state of malnutrition and weakness.	Anthropometric indicators
Mueller-2007	Included	Yes	Defined based on weight for height (W/H) for patients aged 1-14 years and BMI for patients aged >14 years.<br>Not malnourished defined as BMI >18 kg/m2 or W/H >80%; moderately malnourished defined if BMI 16-18 or W/H 70-80%; severely malnourished defined as BMI <16 or W/H < 70%.	Anthropometric indicators
Cota-2014	Included	Not defined	Not defined	Not defined

Mbui-2018	Excluded	Yes	Based on WHO standardized nutritional status. Children with severe malnutrition were excluded (z-score <−3). For children aged <5 years, weight for height was used: children were considered underweight when their z-score <−2 and overweight when their z-score >2. For children aged 5–12 years, body mass index for age was used: children were considered underweight when their z-score <−2 and overweight when their z-score >1.	Anthropometric indicators
Borges-2017	Excluded	Yes	Severe malnutrition defined according to Gómez criteria, presence of comorbidities or immunosuppressive conditions	Anthropometric indicators
Wasunna-2016	Included	Yes	Patients with severe protein and/or caloric malnutrition defined as kwashiorkor or marasmus in children and BMI <15 in adults were excluded. Nutritional status categorization derived by post-hoc determination of WHO standardized value, as opposed to the BMI threshold in the severely underweight exclusion criterion.	Anthropometric indicators
Rahman-2017	Excluded	Yes	Severe malnutrition [for adults (> 18 years) defined as BMI <14; for children (< 18 years) defined as BMI for age z score < -3 in children measuring > 121.5cm; and weight for height less than 60% in children measuring <121.5 cm]	Anthropometric indicators
Romero-2017	Excluded	Not defined	Severe malnutrition was an exclusion criterion. Not defined.	Not defined
Alborzi-2017	Included	Not defined	Not defined	Not defined
Kimutai-2017	Included	Not defined	Not defined	Not defined
Goyal-2018	Included	Yes	Anthropometric indicators appropriate for patient age were calculated using the latest World Health Organization (WHO) Multicentre Growth Reference. Severe wasting was defined based on WHO criteria (weight for height Z-score <-3 for children <5 years; BMI-for-age Z-score <-3 for those 5–19 years; and BMI <16.0 for adults).	Anthropometric indicators
Ekram-2021	Included	Yes	Gross underweight was measured by BMI. Definition not presented.	Anthropometric indicators

[a] The discussion section of the manuscript states that many of the eligible patients were severely ill with massively enlarged spleens, anaemia, malnutrition, inability to walk unaided, and HIV coinfection.

Supplemental table 5: Inclusion of patients with critically illness from Visceral Leishmaniasis (VL) or with severe Visceral Leishmaniasis (n=89 studies)

**Author year**	**Were patients with severe VL or with critical illness included?**	**Terminology was used to refer disease severity**
Gaeta-2000	Unclear	Disease severity not mentioned
Sundar-2000c	Unclear	Disease severity not mentioned
Thakur-2000a	Excluded	Critical illness
Sundar-2000b	Unclear	Disease severity not mentioned
Sundar-2000a	Unclear	Disease severity not mentioned
Thakur-2000b	Excluded	Critical illness from VL
Veeken-2000	Not Excluded	Severe VL
Villanueva-2000	Unclear	Disease severity not mentioned
Thakur-2001a	Unclear0	Disease severity not mentioned
Moore-2001	Included	Severe VL
Ritmeijer-2001	Included	Severely ill
Thakur-2001b	Unclear	Disease severity not mentioned
Dietze-2001	Unclear	Disease severity not mentioned
Sundar-2001	Unclear	Disease severity not mentioned
Haidar-2001	Unclear	Disease severity not mentioned
Das-2001	Critical illness (not clear if critical illness was from VL or other disease)	Critical illness (not clear if critical illness was from VL or other disease)
Sundar-2002	Excluded	Severe VL
Sundar-2002a	Unclear	Disease severity not mentioned
Laguna-2003	Unclear	Disease severity not mentioned
Sundar-2003b	Included	Severe VL
Figueras Nadal-2003	Unclear	Disease severity not mentioned
Sundar-2003	Unclear	Disease severity not mentioned

Syriopoulou-2003	Unclear	Disease severity not mentioned
Rijal-2003	Unclear	Disease severity not mentioned
Thakur-2004a	Unclear	Disease severity not mentioned
Sundar-2004	Included	Clinically severe VL
Thakur-2004b	Unclear	Disease severity not mentioned
Bhattacharya-2004	Excluded	Severe VL
Wasunna-2005	Excluded	Severe VL
Jha-2005	Excluded	Severe VL
Das-2005	Unclear	Disease severity not mentioned
Ritmeijer-2006	Unclear	Disease severity not mentioned
Sundar-2006	Unclear	Disease severity not mentioned
Singh-2006	Unclear	Disease severity not mentioned
Singh-2006	Unclear	Disease severity not mentioned
Sundar-2007b	Included	Clinically severe VL
Sundar-2007a	Unclear	Disease severity not mentioned
Bhattacharya-2007	Unclear	Severity mentioned<br>(Not clear if Severity was used for referring to adverse events or for disease severity grading)
Mueller-2007	Included	Complicated cases<br>(Not explicit if this meant complicated VL or complications due to other infections)
Sundar-2008b	Unclear	Disease severity not mentioned
Sundar-2008a	Unclear	Disease severity not mentioned
Thakur-2008	Unclear	Disease severity not mentioned
Mueller-2008	Unclear	Disease severity not mentioned
Sundar-2009a	Unclear	Disease severity not mentioned
Das-2009	Unclear	Disease severity not mentioned
Sundar-2009b	Unclear	Disease severity not mentioned
Adam-2009	Unclear	Disease severity not mentioned

Shahian-2009	Unclear	Disease severity not mentioned
Hailu-2010	Unclear	Disease severity not mentioned
Thakur-2010	Unclear	Disease severity not mentioned
Musa-2010	Unclear	Disease severity not mentioned
Sundar-2010	Included	Clinically severe VL
Mondal-2010	Unclear	Disease severity not mentioned
Rijal-2010	Unclear	Disease severity not mentioned
Singh-2010	Unclear	Disease severity not mentioned
Sinha-2010	Unclear	Disease severity not mentioned
Sundar-2011b	Unclear	Disease severity not mentioned
Sundar-2011c	Unclear	Disease severity not mentioned
Sundar-2011a	Excluded	Mild-moderate VL
Rahman-2011	Unclear	Disease severity not mentioned
Sinha-2011	Unclear	Disease severity not mentioned
Sudarshan-2011	Unclear	Disease severity not mentioned
Sundar-2012	Unclear	Disease severity not mentioned
Musa-2012	Excluded	Very severe VL
Patra-2012	Unclear	Disease severity not mentioned
Rijal-2013	Unclear	Disease severity not mentioned
Khalil-2014	Excluded	Severe VL
Ostyn-2014	Unclear	Disease severity not mentioned
Cota-2014	Unclear	Disease severity not mentioned
Sundar-2014	Unclear	Disease severity not mentioned
Mondal-2014	Unclear	Disease severity not mentioned
Jamil-2015	Unclear	Disease severity not mentioned
Sundar-2015	Unclear	Disease severity not mentioned
Goswami-2016	Unclear	Disease severity not mentioned
Wasunna-2016	Unclear	Disease severity not mentioned

Pandey-2016	Unclear	Disease severity not mentioned
Borges-2017	Excluded	Severe illness/severe cases
Rahman-2017	Excluded	Non-severe VL
Romero-2017	Excluded	Severe VL
Alborzi-2017	Excluded	Severe VL
Pandey-2017	Unclear	Severely ill<br>(Not clear if this meant severe illness at presentation or severe illness during the follow-up phase)
Kimutai-2017	Unclear	Disease severity not mentioned
Mbui-2018	Excluded	Severe VL
Goyal-2018	Unclear	Disease severity not mentioned
Sundar-2019	Unclear	Disease severity not mentioned
Diro-2019	Unclear	Disease severity not mentioned
Sinha-2019	Excluded	Severe VL
Goswami-2020	Unclear	Disease severity not mentioned
Ekram-2021	Unclear	Disease severity not mentioned

Supplemental table 6: Case definitions and case confirmation approach (n=89 studies)

Author year	Case definition method	Compatible signs and symptoms	Case confirmation method
Gaeta-2000	Compatible clinical diagnosis	Fever + splenomegaly + pancytopenia	Parasitological
Sundar-2000c	Compatible clinical diagnosis	Fever + splenomegaly + weight loss + weakness	Parasitological
Thakur-2000a	Compatible clinical diagnosis	Fever + splenomegaly/hepatosplenomegaly + weight loss/loss of appetite	Parasitological
Sundar-2000b	Compatible clinical diagnosis	Fever + weight loss + hepatosplenomegaly + pancytopenia	Serological and/or parasitological
Sundar-2000a	Compatible clinical diagnosis	Fever + splenomegaly/hepatosplenomegaly + weight loss/loss of appetite	Serological and/or parasitological
Thakur-2000b	Compatible clinical diagnosis	Unclear	Serological and/or parasitological
Veeken-2000	Compatible clinical diagnosis	Fever + splenomegaly/wasting	Serological and/or parasitological
Villanueva-2000	Not presented	Unclear	Parasitological
Haidar-2001	Compatible clinical diagnosis	Unclear	Serological and/or parasitological
Thakur-2001a	Compatible clinical diagnosis	Fever + splenomegaly	Parasitological
Moore-2001	Compatible clinical diagnosis	Fever + splenomegaly/wasting	Serological and/or parasitological
Ritmeijer-2001	Compatible clinical diagnosis	Fever + splenomegaly/wasting	Serological and/or parasitological
Thakur-2001b	Not presented	Unclear	Parasitological

Dietze-2001	Compatible clinical diagnosis	Unclear	Serological and/or parasitological
Sundar-2001	Compatible clinical diagnosis	Fever + splenomegaly/hepatosplenomegaly + weight loss/loss of appetite	Serological and/or parasitological
Das-2001	Not presented	Unclear	Parasitological
Sundar-2002	Compatible clinical diagnosis	Fever + splenomegaly + cytopenia	Parasitological
Sundar-2002a	Compatible clinical diagnosis	Unclear	Serological and/or parasitological
Laguna-2003	Compatible clinical diagnosis	Unclear	Parasitological
Sundar-2003b	Compatible clinical diagnosis	Fever + chills + rigor + splenomegaly	Serological and/or parasitological
Figueras Nadal-2003	Compatible clinical diagnosis	Unclear	Serological and/or parasitological
Sundar-2003	Compatible clinical diagnosis	Unclear	Serological and/or parasitological
Syriopoulou-2003	Compatible clinical diagnosis	Fever + splenomegaly + anaemia + leukopenia/thrombocytopenia	Serological and/or parasitological
Rijal-2003	Compatible clinical diagnosis	Fever + splenomegaly	Serological and/or parasitological
Thakur-2004a	Not presented	Unclear	Parasitological
Sundar-2004	Compatible clinical diagnosis	Fever + splenomegaly/hepatosplenomegaly + weight loss/loss of appetite	Parasitological

Thakur-2004b	Not presented	Unclear	Parasitological
Bhattacharya-2004	Compatible clinical diagnosis	Fever + splenomegaly + anaemia + cytopenia	Serological and/or parasitological
Wasunna-2005	Compatible clinical diagnosis	Unclear	Parasitological
Jha-2005	Compatible clinical diagnosis	Unclear	Parasitological
Das-2005	Compatible clinical diagnosis	Fever + splenomegaly	Serological and/or parasitological
Ritmeijer-2006	Compatible clinical diagnosis	Fever + wasting + splenomegaly/lymphadenopathy	Serological and/or parasitological
Sundar-2006	Compatible clinical diagnosis	Fever + splenomegaly	Parasitological
Singh-2006	Compatible clinical diagnosis	Fever + splenomegaly	Serological and/or parasitological
Singh-2006	Compatible clinical diagnosis	Fever + splenomegaly	Serological and/or parasitological
Sundar-2007b	Compatible clinical diagnosis	Fever + splenomegaly/hepatosplenomegaly + weight loss/loss of appetite	Parasitological
Sundar-2007a	Compatible clinical diagnosis	Unclear	Serological and/or parasitological
Bhattacharya-2007	Compatible clinical diagnosis	Fever or splenomegaly or cytopenia	Serological and/or parasitological
Mueller-2007	Compatible clinical diagnosis	Fever + weight loss + splenomegaly/lymphadenopathy + hepatomegaly + epistaxis + anaemia	Serological and/or parasitological

Sundar-2008b	Compatible clinical diagnosis	Fever + splenomegaly/hepatosplenomegaly + weight loss/loss of appetite	Parasitological
Sundar-2008a	Compatible clinical diagnosis	Fever + splenomegaly/hepatosplenomegaly + weight loss/loss of appetite	Parasitological
Thakur-2008	Not presented	Unclear	Parasitological
Mueller-2008	Compatible clinical diagnosis	Unclear	Serological and/or parasitological
Sundar-2009a	Compatible clinical diagnosis	Fever + splenomegaly/hepatosplenomegaly + weight loss/loss of appetite	Parasitological
Das-2009	Compatible clinical diagnosis	Fever + rigor + hepatosplenomegaly + anorexia + weight loss + anaemia	Parasitological
Sundar-2009b	Compatible clinical diagnosis	Fever + splenomegaly/hepatosplenomegaly + weight loss/loss of appetite	Serological and/or parasitological
Adam-2009	Compatible clinical diagnosis	Fever + weight loss + anaemia + hepatosplenomegaly	Serological and/or parasitological
Shahian-2009	Compatible clinical diagnosis	Fever + hepatomegaly/splenomegaly	Serological and/or parasitological
Hailu-2010	Compatible clinical diagnosis	Fever + splenomegaly	Serological and/or parasitological
Thakur-2010	Compatible clinical diagnosis	Fever + shivering + hepatosplenomegaly + leukopenia	serological + parasitological
Musa-2010	Compatible clinical diagnosis	Unclear	Serological and/or parasitological

Sundar-2010	Compatible clinical diagnosis	Fever + splenomegaly/hepatosplenomegaly + weight loss/loss of appetite	Serological and/or parasitological
Mondal-2010	Compatible clinical diagnosis	Fever + hepatomegaly/splenomegaly	serological + parasitological
Rijal-2010	Compatible clinical diagnosis	Fever + splenomegaly	Serological and/or parasitological
Singh-2010	Compatible clinical diagnosis	Unclear	Serological and/or parasitological
Sinha-2010	Compatible clinical diagnosis	Fever + splenomegaly + weight loss + anaemia	Serological and/or parasitological
Sundar-2011b	Compatible clinical diagnosis	Fever + splenomegaly	Serological and/or parasitological
Sundar-2011c	Compatible clinical diagnosis	Fever + splenomegaly/hepatosplenomegaly + weight loss/loss of appetite	Parasitological
Sundar-2011a	Not presented	Unclear	Parasitological
Rahman-2011	Compatible clinical diagnosis	Fever + splenomegaly/hepatosplenomegaly + weight loss/loss of appetite	Serological and/or parasitological
Sinha-2011	Compatible clinical diagnosis	Unclear	Serological and/or parasitological
Sudarshan-2011	Not presented	Unclear	Parasitological
Sundar-2012	Compatible clinical diagnosis	Fever + chills + rigor + splenomegaly	Serological and/or parasitological
Musa-2012	Compatible clinical diagnosis	Fever + splenomegaly	Serological and/or parasitological
Patra-2012	Compatible clinical diagnosis	Fever + hepatosplenomegaly + anaemia + lymphadenopathy	serological + parasitological
Rijal-2013	Compatible clinical diagnosis	Fever + splenomegaly	Serological and/or parasitological
Khalil-2014	Compatible clinical diagnosis	Fever or splenomegaly or cytopenia	Serological and/or parasitological
Ostyn-2014	Not presented	Unclear	Serological and/or parasitological

Cota-2014	Compatible clinical diagnosis	Fever or splenomegaly or cytopenia	Serological and/or parasitological
Sundar-2014	Compatible clinical diagnosis	Fever + splenomegaly	serological + parasitological
Mondal-2014	Compatible clinical diagnosis	Fever + splenomegaly	Serological and/or parasitological
Jamil-2015	Compatible clinical diagnosis	Fever + splenomegaly/hepatosplenomegaly + weight loss/loss of appetite	Serological and/or parasitological
Sundar-2015	Not presented	Unclear	Parasitological
Goswami-2016	Compatible clinical diagnosis	Fever + splenomegaly	serological + parasitological
Wasunna-2016	Not presented	Unclear	Parasitological
Pandey-2016	Compatible clinical diagnosis	Fever + splenomegaly	Serological and/or parasitological
Borges-2017	Compatible clinical diagnosis	Fever + splenomegaly	Serological and/or parasitological
Rahman-2017	Not presented	Unclear	serological + parasitological
Romero-2017	Compatible clinical diagnosis	Fever + hepatomegaly/splenomegaly	Serological and/or parasitological
Alborzi-2017	Compatible clinical diagnosis	Fever + splenomegaly	Serological and/or parasitological
Pandey-2017	Compatible clinical diagnosis	Unclear	serological + parasitological
Kimutai-2017	Compatible clinical diagnosis	Unclear	Serological and/or parasitological
Mbui-2018	Compatible clinical diagnosis	Unclear	Serological and/or parasitological
Goyal-2018	Compatible clinical diagnosis	Fever + splenomegaly	Serological and/or parasitological
Sundar-2019	Compatible clinical diagnosis	Fever + chills + rigor + splenomegaly	Serological and/or parasitological

Diro-2019	Compatible clinical diagnosis	Fever + splenomegaly/hepatosplenomegaly + weight loss/loss of appetite	Serological and/or parasitological
Sinha-2019	Compatible clinical diagnosis	Fever + splenomegaly + anaemia + cytopenia	Serological and/or parasitological
Goswami-2020	Compatible clinical diagnosis	Fever + splenomegaly + hepatomegaly + anaemia	Serological and/or parasitological
Ekram-2021	Compatible clinical diagnosis	Unclear	Serological and/or parasitological

Supplemental table 7: Reasons for patient exclusion in studies that reported patient flow (n=46 studies)

Total screened	22,056
Total enrolled	13,878
Total Excluded	8,178
Reasons for exclusion (n=8,178)	
Unclear reasons	2,800
Negative for VL upon further tissue examination†	2,723
Biochemistry measurements outside of eligible range	687
Age outside the eligibility range	515
Refused to participate/consent not given	350
Didn't meet eligibility criteria (reasons not stated)	173
Unclear comorbidities	165
Severe VL	147
HIV	129
Previously treated	103
Pregnant	81
Nomadic lifestyle/inability to return to follow-up visits	80
Female of child bearing age	47
Malnutrition	42
History of drug abuse	26
Severe anaemia	25
Hepatitis	23
Hearing disorder	18

Shortage of drug	12
Drug contradicted	8
Cardiac disorder	6
Death before treatment	6
Kidney disorder	4
Diabetes	3
PKDL	2
TB/bronchitis/pneumonia	2
Hypertension	1

† Further details of these studies are presented in supplemental table 8

Supplemental table 8: Description of the studies with patients excluded for negative VL upon parasitological examination (n=13 studies)

**Author year**	**Region**	**Number Screened**	**Number excluded for being VL negative**	**Compatible signs and symptoms used as case definition**	**Diagnostic method used for VL confirmation**	**Tissue aspirate used**
Hailu-2010	Eastern Africa	926	221	Fever + splenomegaly	Parasitological	Bone marrow or spleen or lymph node
Musa-2010	Eastern Africa	104	35	Unclear	Parasitological	Bone marrow
Rijal-2010	India Subcontinent	301	77	Fever ≥2 weeks + splenomegaly	Parasitological	Bone marrow and/or splenic aspirate
Sundar-2011b	India Subcontinent	896	123	Fever + splenomegaly	Serological and/or parasitological	Bone marrow and/or splenic aspirate
Musa-2012	Eastern Africa	2862	953	Fever + splenomegaly	Parasitological	Bone marrow or spleen or lymph node
Rijal-2013	India Subcontinent	187	26	Fever ≥2 weeks + splenomegaly	Parasitological + culture	Bone marrow and/or splenic aspirate
Khalil-2014	Eastern Africa	811	468	Fever ≥2 weeks or splenomegaly or cytopenia	Parasitological and molecular	Bone marrow and/or splenic aspirate and blood
Cota-2014	South America	168	78	Fever ≥2 weeks or splenomegaly or cytopenia	Serological and/or parasitological	Bone marrow
Mondal-2014	India Subcontinent	594	269	Fever ≥2 weeks + splenomegaly	Serological	Blood
Wasunna-2016	Eastern Africa	970	439	Unclear	Parasitological	Bone marrow or spleen or lymph node
Rahman-2017	India Subcontinent	673	24	Unclear	Serological and/or parasitological	Bone marrow and/or splenic aspirate
Mbui-2018	Eastern Africa	158	3	Unclear	Parasitological	Bone marrow and/or splenic aspirate
Ekram-2021	India Subcontinent	41	7	Unclear	Parasitological	Bone marrow and/or splenic aspirate

Supplemental table 9: Diagnostic methods used for enrolment and outcome assessment for relapse (n=89 studies)

Author year	Diagnostic method used for Disease confirmation	Tissue aspirates	Quality control of laboratory procedures	Zymodeme analysis	Genotyping for relapse
Gaeta-2000	Parasitological + culture	Bone marrow	No information	No information	No
Sundar-2000c	Parasitological	Spleen	No information	No information	No
Thakur-2000a	Parasitological	Bone marrow and/or splenic aspirate	No information	No information	No
Sundar-2000b	Parasitological	Bone marrow and/or splenic aspirate	No information	No information	No
Sundar-2000a	Parasitological	Spleen	No information	No information	No
Thakur-2000b	Parasitological	Bone marrow and/or splenic aspirate	No information	No information	No
Veeken-2000	Serological and/or parasitological	Bone marrow or spleen or lymph node	Random samples checked	No information	No

Villanueva-2000	Parasitological + culture	Combination	No information	Yes	No
Thakur-2001a	Parasitological	Spleen	No information	No information	No
Moore-2001	Serological and/or parasitological	Spleen	independent microscopist	No information	No
Ritmeijer-2001	Serological and/or parasitological	Spleen	independent microscopist	No information	No
Thakur-2001b	Parasitological	bone marrow and/or splenic aspirate	No information	No information	No
Dietze-2001	Parasitological + culture	Spleen	No information	No information	No
Sundar-2001	Parasitological	Spleen	No information	No information	No
Haidar-2001	Parasitological	bone marrow and/or splenic aspirate	No information	No information	No
Das-2001	Parasitological	bone marrow and/or splenic aspirate	No information	No information	No
Sundar-2002	Parasitological	Spleen	No information	No information	No
Sundar-2002a	Parasitological	bone marrow and/or splenic aspirate	No information	No information	No
Laguna-2003	Parasitological + culture	Combination	No information	No information	Yes
Sundar-2003b	Parasitological	bone marrow and/or splenic aspirate	Single reader	No information	No

Figueras Nadal-2003	Serological and/or parasitological + culture	Bone marrow	No information	No information	No
Sundar-2003	Parasitological	Spleen	No information	No information	No
Syriopoulou-2003	Serological and/or Parasitological	Bone marrow	experienced technician used	No information	No
Rijal-2003	Parasitological	bone marrow and/or splenic aspirate	External checking of 10% of slides	No information	No
Thakur-2004a	Parasitological	bone marrow and/or splenic aspirate	No information	No information	No
Sundar-2004	Parasitological	Spleen	No information	No information	No
Thakur-2004b	Parasitological + culture	Bone marrow	No information	No information	No
Bhattacharya-2004	Parasitological	Spleen	No information	No information	No
Wasunna-2005	Parasitological	Spleen	No information	No information	No
Jha-2005	Parasitological	Spleen	No information	No information	No

Das-2005	Parasitological	bone marrow and/or splenic aspirate	No information	No information	No
Ritmeijer-2006	Serological and/or parasitological	Combination	No information	No information	No
Sundar-2006	Parasitological	Spleen	No information	No information	No
Singh-2006	Parasitological	Spleen	No information	No information	No
Singh-2006	Parasitological	Spleen	No information	No information	No
Sundar-2007b	Parasitological	Spleen	No information	No information	No
Sundar-2007a	Parasitological	bone marrow and/or splenic aspirate	No information	No information	No
Bhattacharya-2007	Parasitological	Spleen	No information	No information	No
Mueller-2007	Serological and/or parasitological	bone marrow or spleen or lymph node	No information	No information	No
Sundar-2008b	Parasitological	Spleen	No information	No information	No
Sundar-2008a	Parasitological	Spleen	No information	No information	No
Thakur-2008	Parasitological	bone marrow and/or splenic aspirate	No information	No information	No
Mueller-2008	Serological and/or parasitological	Spleen	No information	No information	No
Sundar-2009a	Parasitological	bone marrow and/or splenic aspirate	No information	No information	No
Das-2009	Parasitological	bone marrow and/or splenic aspirate	No information	No information	No

Sundar-2009b	Parasitological	Spleen	No information	No information	No
Adam-2009	Parasitological	Bone marrow	No information	No information	No
Shahian-2009	Serological	Unclear	No information	No information	No
Hailu-2010	Parasitological	bone marrow or spleen or lymph node	No information	No information	No
Thakur-2010	Serological and/or parasitological	bone marrow and/or splenic aspirate	No information	No information	No
Musa-2010	Parasitological	Bone marrow	Trained lab tech	No information	No
Sundar-2010	Parasitological	Spleen	Two readers	No information	No
Mondal-2010	Serological and/or parasitological	bone marrow and/or splenic aspirate	No information	No information	No
Rijal-2010	Parasitological	bone marrow and/or splenic aspirate	No information	No information	No
Singh-2010	Parasitological	Bone marrow	No information	No information	No
Sinha-2010	Serological and/or parasitological	Combination	No information	No information	No

Sundar-2011b	Serological and/or parasitological	bone marrow and/or splenic aspirate	External checking of 10% of slides	No information	No
Sundar-2011c	Parasitological	bone marrow and/or splenic aspirate	No information	No information	No
Sundar-2011a	Parasitological	Spleen	No information	No information	No
Rahman-2011	Serological	Blood	No information	No information	No
Sinha-2011	Serological and/or parasitological	bone marrow or spleen or lymph node	No information	No information	No
Sudarshan-2011	Parasitological	Spleen	No information	No information	PCR used on blood sample for parasite clearance but not for genotyping
Sundar-2012	Parasitological	Spleen	Two readers	No information	No
Musa-2012	Parasitological	bone marrow or spleen or lymph node	No information	No information	No
Patra-2012	Serological and/or parasitological + culture	bone marrow and/or splenic aspirate	No information	No information	No
Rijal-2013	Parasitological + culture	bone marrow and/or splenic aspirate	No information	No information	Yes

Khalil-2014	Parasitological and molecular	bone marrow and/or splenic aspirate and blood	No information	Yes (S1 text)	No
Ostyn-2014	Serological and/or parasitological	Unclear	No information	No information	No
Cota-2014	Serological and/or parasitological	Bone marrow	No information	No information	No
Sundar-2014	Serological and/or parasitological	bone marrow and/or splenic aspirate	No information	No information	No
Mondal-2014	Serological	Blood	No information	No information	No
Jamil-2015	Serological	Unclear	No information	No information	No
Sundar-2015	Parasitological	Unclear	No information	No information	No
Goswami-2016	Serological and/or parasitological	Unclear	No information	No information	No
Wasunna-2016	Parasitological	bone marrow or spleen or lymph node	Two slides per sample	No information	No
Pandey-2016	Serological and/or parasitological	Combination	No information	No information	No
Borges-2017	Serological and/or parasitological and/or molecular + culture	Combination	No information	No information	PCR used in 4 patients for diagnosis
Rahman-2017	Serological and/or parasitological	bone marrow and/or splenic aspirate	No information	No information	No
Romero-2017	Serological and/or parasitological + culture	Combination	No information	No information	No

Alborzi-2017	Serological and/or parasitological	Bone marrow	No information	No information	No
Pandey-2017	Serological and/or parasitological	bone marrow and/or splenic aspirate	No information	No information	No
Kimutai-2017	Serological and/or Parasitological and/or Clinical	Combination	No information	No information	No
Mbui-2018	Parasitological	bone marrow and/or splenic aspirate	No information	No information	No
Goyal-2018	Serological	Blood	No information	No information	No
Sundar-2019	Serological	Blood	No information	No information	No
Diro-2019	Parasitological	bone marrow and/or splenic aspirate	No information	No information	No
Sinha-2019	Parasitological	Spleen	No information	No information	No
Goswami-2020	Serological and/or parasitological	bone marrow and/or splenic aspirate	No information	No information	No
Ekram-2021	Parasitological	bone marrow and/or splenic aspirate	No information	No information	No

Supplemental table 10: Methodology adopted for outcome assessments (n=89 studies)

Author year	Test of cure assessment	End of study assessment	Tissue aspirates at test of cure	Tissue aspirates for confirmation of relapse
Gaeta-2000	Parasitological	Clinical + parasitological	Bone marrow	Aspiration not done
Sundar-2000c	Clinical + parasitological	Clinical + parasitological	Spleen	Bone marrow
Thakur-2000a	Parasitological	Clinical + parasitological	Bone marrow/spleen	Done (unclear aspirate)
Sundar-2000b	Clinical + parasitological	Clinical	Bone marrow/spleen	Unclear if aspiration done
Sundar-2000a	Clinical + parasitological	Clinical + parasitological	Spleen	Bone marrow
Thakur-2000b	Clinical + parasitological	Clinical + parasitological	Bone marrow/spleen	Done (unclear aspirate)
Veeken-2000	Clinical	Clinical + parasitological	Lymph node	Done (unclear aspirate)
Villanueva-2000	Clinical + parasitological	Clinical + parasitological	Bone marrow	Bone marrow
Thakur-2001a	Clinical + parasitological	Clinical + parasitological	Spleen	Spleen
Moore-2001	Clinical + parasitological	Clinical and/or parasitological	Spleen	Spleen
Ritmeijer-2001	Clinical and/or parasitological	Clinical and/or parasitological	Spleen	Done (unclear aspirate)
Thakur-2001b	Clinical + parasitological	Clinical + parasitological	Spleen	Done (unclear aspirate)
Dietze-2001	Parasitological	Clinical + parasitological	Bone marrow/spleen	Spleen
Sundar-2001	Clinical + parasitological	Clinical + parasitological	Spleen	Spleen

Haidar-2001	Clinical + parasitological	Unclear	Bone marrow/spleen	Monitored but not defined
Das-2001	Clinical + parasitological	Clinical + parasitological	Bone marrow/spleen	Bone marrow
Sundar-2002	Parasitological	Unclear	Done (unclear aspirate)	Spleen
Sundar-2002a	Clinical + parasitological	Clinical + parasitological	Bone marrow/spleen	Bone marrow and/or spleen
Laguna-2003	Parasitological	Clinical + parasitological	Bone marrow/spleen/liver	Bone marrow or tissue biopsy
Sundar-2003b	Clinical + parasitological	Clinical + parasitological	Bone marrow/spleen	Spleen
Figueras Nadal-2003	Clinical + parasitological	Clinical + parasitological	Bone marrow	Reported but not defined
Sundar-2003	Clinical + parasitological	Clinical + parasitological	Spleen	Spleen
Syriopoulou-2003	Clinical + parasitological	Unclear	Bone marrow	Bone marrow
Rijal-2003	Clinical + parasitological	Clinical + parasitological	Done (unclear aspirate)	Done (unclear aspirate)
Thakur-2004a	Clinical + parasitological	Clinical + parasitological	Spleen	Spleen
Sundar-2004	Clinical + parasitological	Clinical + parasitological	Spleen	Done (unclear aspirate)
Thakur-2004b	Clinical + parasitological	Clinical + parasitological	Bone marrow/spleen	Done (unclear aspirate)
Bhattacharya-2004	Clinical + parasitological	Clinical + parasitological	Done (unclear aspirate)	Spleen
Wasunna-2005	Parasitological	Clinical + parasitological	Spleen	Spleen
Jha-2005	Parasitological	Clinical + parasitological	Spleen	Spleen
Das-2005	Clinical + parasitological	Clinical + parasitological	Done (unclear aspirate)	Bone marrow and/or spleen

Ritmeijer-2006	Clinical/parasitological	Clinical + parasitological	Spleen or lymph	Done (unclear aspirate)
Sundar-2006	Clinical + parasitological	Clinical + parasitological	Spleen	Spleen
Singh-2006	Parasitological	Clinical + parasitological	Done (unclear aspirate)	Spleen
Singh-2006	Parasitological	Clinical + parasitological	Done (unclear aspirate)	Spleen
Sundar-2007b	Clinical + parasitological	Clinical + parasitological	Spleen	Done (unclear aspirate)
Sundar-2007a	Clinical + parasitological	Clinical + parasitological	Done (unclear aspirate)	Bone marrow and/or spleen
Bhattacharya-2007	Parasitological	Clinical + parasitological	Spleen	Spleen
Mueller-2007	Clinical + parasitological	Clinical	Lymph node	Not actively assessed
Sundar-2008b	Clinical + parasitological	Clinical + parasitological	Spleen	Done (unclear aspirate)
Sundar-2008a	Clinical + parasitological	Clinical + parasitological	Spleen	Unclear if aspiration done
Thakur-2008	Clinical + parasitological	Clinical + parasitological	Done (unclear aspirate)	Bone marrow/spleen
Mueller-2008	Clinical + parasitological	Clinical + parasitological	Spleen	Mentioned but not defined

Sundar-2009a	Parasitological	Clinical + parasitological	Spleen	Unclear if aspiration done
Das-2009	Clinical + parasitological	Clinical + parasitological	Bone marrow/spleen	Spleen
Sundar-2009b	Clinical + parasitological	Clinical + parasitological	Spleen	Spleen
Adam-2009	No information	Clinical	No information	No information
Shahian-2009	No information	Clinical	Unclear	Clinical suspicion only
Hailu-2010	Not defined	Clinical + parasitological	Spleen/lymph/bone marrow	spleen/lymph/bone marrow
Thakur-2010	Clinical + parasitological	Clinical + parasitological	Spleen	Spleen
Musa-2010	Parasitological	Clinical + parasitological	Done (unclear aspirate)	Done (unclear aspirate)
Sundar-2010	Clinical + parasitological	Clinical	Spleen	Done (unclear aspirate)
Mondal-2010	Clinical	Clinical + parasitological	Unclear	Spleen
Rijal-2010	Clinical + parasitological	Clinical + parasitological	Done (unclear aspirate)	Done (unclear aspirate)
Singh-2010	Parasitological	Clinical + parasitological	Done (unclear aspirate)	Bone marrow
Sinha-2010	Clinical + parasitological	Clinical + parasitological	Bone marrow/spleen	Bone marrow/spleen
Sundar-2011b	Clinical + parasitological	Clinical + parasitological	Bone marrow/spleen	Unclear if aspiration done
Sundar-2011c	Parasitological	Clinical	Spleen	Spleen and/or bone marrow
Sundar-2011a	Clinical + parasitological	Clinical + parasitological	Spleen	spleen

Rahman-2011	Clinical + parasitological	Clinical + parasitological	Bone marrow/spleen	Bone marrow/spleen
Sinha-2011	Clinical	Clinical	Unclear	Bone marrow/spleen
Sudarshan-2011	Parasitological	Clinical + parasitological	Peripheral blood	Peripheral blood
Sundar-2012	Clinical + parasitological	Clinical + parasitological	Spleen	Spleen
Musa-2012	Parasitological	Clinical + parasitological	Spleen/bone marrow/lymph	Bone marrow/lymph/spleen
Patra-2012	Parasitological	Clinical + parasitological	Done (unclear aspirate)	bone marrow and/or spleen
Rijal-2013	Clinical + parasitological	Clinical + parasitological + molecular	Bone marrow	Done (unclear aspirate)
Khalil-2014	Parasitological	Clinical + parasitological	Done (unclear aspirate)	Bone marrow/lymph/spleen
Ostyn-2014	Clinical	Clinical + parasitological	Unclear	Done (unclear aspirate)
Cota-2014	Clinical	Clinical	Not carried out	Bone marrow
Sundar-2014	Parasitological	Clinical	Bone marrow/spleen	Mentioned but not defined
Mondal-2014	Clinical	Clinical + parasitological	Unclear	Spleen
Jamil-2015	Clinical	Clinical	Unclear	Mentioned but not defined
Sundar-2015	Clinical + parasitological	Clinical + parasitological	Bone marrow/spleen	Spleen
Goswami-2016	Clinical + parasitological	Clinical + parasitological	Bone marrow/spleen	Spleen
Wasunna-2016	Parasitological	Clinical + parasitological	Bone marrow/spleen/lymph node	Done (unclear aspirate)
Pandey-2016	Clinical	Unclear	Unclear	Mentioned but not defined
Borges-2017	Clinical	Clinical	Unclear	Reported but not defined

Rahman-2017	Clinical + parasitological	Clinical	Bone marrow/spleen	Done (unclear aspirate)
Romero-2017	Clinical	Clinical	Unclear	Clinical suspicion only
Alborzi-2017	Clinical	Clinical + parasitological/serological	Spleen	Done (unclear aspirate)
Pandey-2017	Clinical + parasitological	Unclear	Bone marrow/spleen	Spleen
Kimutai-2017	Clinical + parasitological	Clinical + parasitological	Done (unclear aspirate)	Done (unclear aspirate)
Mbui-2018	Clinical + parasitological	Clinical + parasitological	Bone marrow/spleen	Reported but not defined
Goyal-2018	No information	Clinical + parasitological	Unclear if aspiration done	bone marrow and/or spleen
Sundar-2019	Clinical	Clinical + parasitological	Spleen	spleen
Diro-2019	Parasitological	Unclear	Bone marrow/spleen	Assessed but not reported in this publication
Sinha-2019	Clinical + parasitological	Clinical + parasitological	Done (unclear aspirate)	Spleen
Goswami-2020	Not defined	Clinical + parasitological	Done (aspirates not clear)	Spleen
Ekram-2021	Clinical	Clinical	Unclear	Mentioned but not defined

Supplemental table 11: Details of sample size estimation (n=34 studies)

Author-year	Details of the sample size estimation	Adjustment for lost to follow-up
Veeken-2000	Power of 90% and a significance level of *P*=0.05 (two-tailed), to detect a difference of 10% in cure, death, or relapse rate between the two groups, a sample size of 207 patients in each arm was required.	No information
Moore-2001	Makuch & Simon's formula (3, 4) can be used to calculate sample sizes for comparative studies if the objective is to show that two treatments are equally effective. For this purpose, the difference in outcome that would lead one of the treatments to be discarded as inferior needs to be specified. For kala-azar treatment we judged this to be a 20% difference in cure rate. Since PSM has repeatedly been found to have a cure rate of about 95% for kala-azar in Africa, we considered that a 75% cure rate for generic sodium stibogluconate would be clinically unacceptable. Taking a = 0.05 and b = 0.1, we required 25 patients per treatment group. We also tested a second set of assumptions suitable for an unpaired prospective study with a dichotomous outcome (cure versus failure). Assuming the drugs differ by 20% in their cure rate, 95% in one group versus 75% in the other (a = 0.05; b = 0.2), we required 49 patients per treatment group.	No information
Ritmeijer-2001	A sample size of 91 patients in each arm was used, with a 90% power to detect at a significance level of P = 0.05 (2-tailed), a difference of 20% in cure, death, or relapse rate between the 2 group.	No information
Das-2001	Assuming a difference of 15% in the efficacy rate between two regimen groups and at level of significance p=0.05 and power of the test at 90%, a sample size of 158 patients unresponsive to antimony were allocated randomly into two groups, using random number table.	No information
Sundar-2002	The considerations for the sample size and the confirmatory analysis were determined on the basis of the restricted maximum-likelihood estimation of the variance of the test statistic, 15 with a one-sided alpha of 0.025, a power of 0.80, a margin of noninferiority of 15 percent, assumed cure rates for Miltefosine of 88 to 92 percent and for amphotericin of 94 to 98 percent, and a ratio of 3:1 for the random assignment of patients to Miltefosine and amphotericin. The number of patients required overall was 400.	No information
Sundar-2002a	The sample size calculation was based on a complete cure rate hypothesis of 90% for the highest dose and 50% for the lowest dose. To detect a difference of 40% in cure rates with a power of 80% and a significance level of 5% in a 2-tailed approach, 25 patients per treatment arm were required.	No information
Sundar-2003b	Sample-size calculation was based on the definitive-cure rate of 90%, with a one-sided lower-bound CI of 0.84, a power of 80%, and a significance level of 5%; for this, a minimum of 200 patients were required.	No information

Thakur-2004a	The target sample size, set assuming there would be no drop-outs or spontaneous cures and that the inter-drug difference in the frequencies of cure would be 20% (Dowd, 1979), was 120 patients (i.e. 60/treatment arm).	No drop-outs assumed
Wasunna-2005	Assuming a true response rate of 85%, this calculation showed that 61 patients would be required to detect within 9% of the true response rate at a confidence level of 95%, assuming that the binomial distribution can be approximated by a normal curve. It was subsequently decided to present exact 95% confidence intervals for the primary efficacy outcome calculated using the Clopper Pearson method. With 61 patients and assuming a true response rate of 85%, this method would detect within −11% to +8% of the true response rate at a two-sided confidence level of 95%.	No information
Sundar-2007b	The proportion of patients with a final cure determined the end point of this trial, which was designed to assess noninferiority of the experimental regimens. With a 1-sided a of 0.05, a power of 90%, and a margin of noninferiority of 5%, samples sizes were determined assuming cure rates of 91%-95% for 15 daily infusions of 0.75 or 1 mg/kg of amphotericin B and of95%-99% for 15 alternate- day infusions at the same doses and a ratio of 2:1 for random assignment to daily and alternate-day infusions.	No information
Sundar-2007a	Assuming a 99% cure rate for amphotericin, 666 patients were needed in a 3:1 ratio to support a one-sided, non-inferiority analysis without stratification and with 80% power to detect a type I error rate of 5%.	No information
Sundar-2008b	The study was designed to have a 5% type 1 error and 95% power, considering a failure rate of and <10% to indicate adequate efficacy (the minimum detectable failure rate at the ß = 0.05 level) and a failure rate ≥ 25% to indicate insufficient efficacy. The boundaries of the test were calculated for H0 (p= p0) and Ha (p < pa) with p0= 0.25 and pa=0.10. Based on simulations, we expected the sample path to cross the H0 rejection line with an average sample size of 40 patients and the H0 non-rejection line with an average sample size between 20 and 25 patients. When, after enrolling 45-46 patients per arm, all treatments appeared to be equally and highly effective, an additional 45 consecutive patients were enrolled and non-randomly assigned to a fifth regimen.	No information
Thakur-2008	We needed a sample size of at least 60 based on past experience and assuming a rate of drop out and spontaneous cure of zero and a difference in the rate of cure between the standard treatment and the new treatment of 20 per cent.	No drop-outs assumed
Sundar-2009a	Sample size was determined after discussion with regulatory authorities for this phase II study and Schedule Y (amended 2005) of Drug and Cosmetic act of India.	No information
Das-2009	We believed that the reported cure rate of AMB (based on the literature) would be 95% in the Amphotericin B group, while it would be about 75% in the Pentamidine group. Based on 90% power to detect a significant difference (P = 0.05, two-sided) 100 patients were required for the study. To compensate for a 10%, drop out, an optimum sample size for this clinical trial was 110 patients, which came to 55 patients in each group.	10% drop-out assumed

Sundar-2009b	Assuming a 95% cure rate after 21 days of treatment with paromomycin, 327 patients were needed in a 2:1 ratio to support a 1-sided noninferiority analysis without stratification and with 80% power to detect a type I error rate of 5%.	No information
Hailu-2010	At the start of the trial, it was estimated that 217 patients would be required per arm to detect a 10% difference in efficacy among HIV-uninfected patients at the 5% level, assuming 90% power, 95% efficacy in the reference (SSG) arm, and a 20% drop-out rate from the analysis (10% due to HIV co-infection and 10% due to loss to follow-up between end of treatment and 6-month assessment).	20% drop-out assumed
Thakur-2010	The aim of this study was to reduce complications such as rigor and acute renal failure caused by treatment with only amphotericin B by simultaneous administration of 500mL of physiologic saline and 30 mL (60 meq/L) of KCl in adults and a correspondingly lower dose in children. A sample size of 100 patients in each group was required for 90% power to detect a difference of 11% reduction in complications in the test regimen group compared with the control regimen group at 5% significance level. On the basis of our previous experience, the drop-out rate was assumed to be 15% at this centre. When we adjusting for the compliance rate, the final sample size was estimated to be 115 patients in each group.	15% drop-out assumed
Sundar-2010	Assuming a 99% cure rate for standard treatment with amphotericin B deoxycholate, we determined that we would need to enrol at least 400 patients in a 3:1 ratio to support a one-tailed noninferiority analysis without stratification and with a power of 90% to detect the probability of a type I error of 5%.	No information
Sinha-2010	The patient sample size was set at 250, which is sufficient, with an expected 10% loss to follow-up, to detect a treatment success rate of 95% with ± 4% precision.	10% loss to follow-up
Sundar-2011b	We assumed a definitive cure rate of 97% with the reference drug (amphotericin B) and a non-inferiority margin of 7% for the test groups. With a power of 90% and equal allocation ratio, the sample size per group was 148; on the assumption of a drop-out rate of 5%, 156 patients per group were needed, with a total sample size for four groups of 624 patients. A non-inferiority margin of 7% was chosen because 90% was thought to be the minimum acceptable rate of definitive cure; it was the rate attained by a single 5 mg/kg dose of liposomal amphotericin B in a previous trial.	5% drop-out
Musa-2012	The trial was designed to have 90% power (b=0.1) to detect, at the 5% significance level (a=0.05), an absolute difference in efficacy of 15% between PM and SSG and 10% between SSG & PM and SSG regimens [16]. An 85% efficacy was assumed in the reference arm and adjusting for 10% HIV co-infection and 10% loss to follow- up at 6 months post end of treatment, it was estimated that 404 and 195 patients per arm were required for the respective comparisons. Being HIV-positive was not an exclusion criterion but the original protocol stated that there was to be a sufficient number of patients for a subgroup analysis excluding HIV patients (if deemed necessary).	10% loss to follow-up

Khalil-2014	For the primary endpoint comparison, 120 patients per arm would provide 80% power to detect non-inferiority within a margin of 10%, assuming 95% cure in the reference arm, a one-sided alpha of 0?05 and 15% loss-to-follow-up. In interim analyses, 20 patients per arm would provide 90% power to detect a difference of at least 35% in parasite clearance rate at day 30, assuming 95% cure in the reference arm and a two-sided alpha of 0.05. With 40 patients per arm, there would be 90% power to detect a difference of at least 25% under the same assumptions.	15% loss to follow-up
Sundar-2014	A total of 500 patients in a 3:1 ratio was planned to be enrolled assuming a dropout rate of 20% and non-inferiority margin fixed at 20.10. This was expected to provide an estimated difference in proportions of patients achieving definitive cure for ABLE vs. LAmB equals to zero, with at least 80% power for the non-inferiority test.	20% drop-out
Mondal-2014	A purposive sample size was determined, assuming that at least 85% of patients with visceral leishmaniasis who attended the hospital were eligible to be treated with single-dose liposomal amphotericin B, and aiming to obtain at least 95% cure rate at 6 months and with up to 3% of treated patients developing drug-related adverse events requiring referral to a tertiary hospital for management.	No information
Goswami-2016	A sample size of 75 patients per group was sufficient to detect a clinically important difference of 11% between groups in the definitive cure rate at 6 months using a two- sided Z-test with 80% power and 5% significance level.	No information
Wasunna-2016	The trial used a sequential design with a triangular continuation region [14]. The null hypothesis was that the proportion cured at day 28 (p) is less than or equal to a value p0 which we set to 75%. The alternative hypothesis is that p>p0. If the upper boundary is crossed during an interim analysis, then the null hypothesis is rejected and we conclude p>75%. Crossing the lower boundary at the time of an interim analysis implies that null hypothesis (proportion for which we chose a value of90%. The type I error rate and power of the study were pre-specified cured 75%) is not rejected and there is specified power to exclude a proportion cured as 5% and 95%, respectively (α = β = 0.05). Interim analyses were specified after every 15 patients in each arm. The maximum sample size per arm was 63. The study was designed and analysed according to sequential methods, which have been developed to allow for discrete data analysis after a pre-specified number of patients are recruited. The triangular test is one such method and uses straight line stopping boundaries [13]. The continuation region is closed, which ensures a maximum sample size. A minimum sample size of 30 per arm was imposed to allow for adequate PK assessment. The trial was non-comparative and the sequential analysis was applied to each arm independently, allowing them to potentially stop at different times.	No information
Borges-2017	The original sample size was determined on the basis of the primary outcome endpoint of efficacy. To calculate sample size, an ideal setting with unlimited number of patients was simulated and the formula suggested by Pocock was applied. The required sample size was 283 subjects in each group. As a pilot trial, this study was projected with 50 individuals in each arm to evaluate the protocol.	No information

Rahman-2017	The sample size was calculated assuming a treatment success of 97% in the reference arm (AmBisome) and a margin of non-inferiority of any tested treatment of 7%, leading to a minimally acceptable cure rate of 90% for each treatment. With a power of 90%, the sample size per group in the sample of married women, men and children would be 140. Based on the possible teratogenic effect of Miltefosine and the assumption that 20% of the patients would be single women of child bearing potential, 26 extra patients were to be recruited among these women for both non-Miltefosine groups. Assuming a drop-out rate of 10%, 154 patients were needed in both Miltefosine groups and 183 in both non-Miltefosine groups. The total sample size was calculated to be 674 patients.	10% drop-out
Romero-2017	As the trial objective was to compare three interventions with the standard treatment (MA), the sample size was determined to allow for detection, with 80% power (1- β) and 5% significance level (α = 0.05), of at least 8% difference in efficacy of each treatment arm in relation to the reference arm. Assuming a 90% efficacy in the MA reference arm, and adjusting for a 10% loss to follow-up and 10% to maintain the power of comparison between the patient subgroups with parasitological diagnostic confirmation and only a positive rk39 diagnostic test (as defined in the inclusion criteria), it was estimated 165 participants would be required per treatment arm, for a total sample size of 660 participants. After the AmphoB arm was stopped due to its higher toxicity, the trial's sample size was reviewed. Adjustment for loss to follow-up was reduced to 5%, in accordance with the observations from the trial up to that moment. No more adjustment was made for those patients diagnosed with only a positive rK39 test, due to the wide validation of these tests by TDR/WHO on three continents, which allows for the inclusion of patients with clinical signs and a positive immunochromatographic test [18]. A final total sample size of 426 was calculated, with 142 participants per treatment arm.	5% loss to follow (initially 10% anticipated)
Kimutai-2017	Given the emphasis on safety, the sample size required was based on the probability of detecting AEs with an expected incidence of 1/1000. With a cohort of 2000 patients, there would be an 86% probability of detecting at least one AE with an expected 1/1000 incidence and a 95% probability with a cohort of 3000 patients [20–22]. When the PV program reached a sample size of 3126, the Steering Committee held a final meeting to recommended termination of the PV data collection activities.	No information
Mbui-2018	The minimal sample size was based on pharmacokinetic clinical trial simulations for the primary pharmacokinetic endpoint using the method of Dorlo and colleagues [18]. Including potential non-compliance, this provided a trial sample of 30 patients. For the primary pharmacokinetic endpoint, plasma Miltefosine concentrations were measured in all patients receiving at least 1 Miltefosine dose.	Non-compliance was adjusted for but details not presented

Goyal-2018	Since the objective of the study was to evaluate the effectiveness and safety of each new treatment modality, the sample size requirement was based around the precision with which effectiveness and safety could be estimated. Assuming a risk of failure of 5% at 6-months follow-up, a sample size of 225 patients per arm would allow for an effectiveness estimation with 3% precision. Since treatment modality allocation was planned to be different between sites, and the patient population might not be homogeneous (referral hospital vs PHC in different districts), an adjustment was applied using a conservative design effect of4 to account for between-centre variability. In this case, a failure risk of 5% could be estimated at around 5% precision with 300 patients per arm.	No information
Goswami-2020	We assumed a definitive cure rate of 90.3% with the reference drug (Miltefosine monotherapy) and a non-inferiority margin of 7% for the test groups. With a power of 90% and equal allocation ratio, the sample size per group was 53, with a total sample size of 106. A non-inferiority margin of 7% was chosen because 83% was thought to be the minimum acceptable rate of definitive cure as it was the rate attained by a phase IV trial of Miltefosine for the treatment of Indian patients with VL.	No information

Supplemental table 12: Details of analytical approach used for efficacy estimation (n=89 studies)

Author-year	Description of efficacy estimation	Analysis principle
Ritmeijer-2006	Proportion cured is reported. Data were analysed on an intent-to-treat basis with Epi Info software, 2002 revision 2 (Centers for Disease Control and Prevention). From the discussion section of the manuscript: The final cure rate at 6 months is probably better than indicated in the intent-to-treat analysis, in which patients lost to follow-up are counted as having experienced treatment failure; many of the patients lost to follow-up might, in fact, have been cured.	ITT explicitly reported
Thakur-2001a	A paired t-test and analysis of variance were used to calculate the significance. All 34 patients were cured.	No specific distinction (PP and ITT identical as 100% cured)
Laguna-2003	The proportions of patients with treatment success were compared among treatment groups by means of the Fisher's Exact Test, and their 95% CIs estimated using binomial distribution. Analysis was presented by ITT and on-treatment estimation.	On-treatment analysis and ITT
Sundar-2011b	The primary endpoint of definitive cure rate was analysed for all randomly assigned patients (intention to treat [ITT]) and for the per-protocol patients. The per- protocol group consisted of all patients who were enrolled, had no major protocol deviation, received the full treatment, and were assessed at day 15 or 31, day 45, and 6 months (-2 to +6 weeks).	Both PP and ITT reported
Gaeta-2000	Defervescence occurred by day 3 of treatment (median 48 h, range 36±72) and spleen size was reduced to 3.191 cm below left costal margin by day 7, and to impalpable by day 180. All patients showed rapid clinical response without significant adverse events.	No specific distinction (PP and ITT identical as 100% cured)
Sundar-2008b	Cure rate at day 16 was considered to be the primary end point and, for the purposes of this study, was used as a surrogate for cure rate at month 9. The day 16 cure rate was analysed for every 5 subjects in each non-comparative arm using the triangular test. Overall cure rates, by ITT is presented.	ITT explicitly reported
Sundar-2002	The end point of this trial designed to assess noninferiority was the proportion of patients with a final cure. See Table 2 of the manuscript for further details.	No specific distinction
Sundar-2011c	Safety, and efficacy comparisons were investigated by using descriptive statistics. For safety and efficacy evaluations, combined sitamaquine cohorts were compared with AmB.	No specific distinction
Sundar-2009a	Safety analyses included all patients who received at least one dose of study drug. Patients were considered eligible for efficacy evaluation if study drug was administered on both days and at least one efficacy assessment was conducted. Data are expressed as mean ± SD. Analysis of variance was used to detect differences among the clinical and biochemical results of the four regimens. Frequency and the percentage of patients who achieved efficacy endpoints (clinical improvement, initial cure, and definite cure) were calculated and compared across the treatment groups by using the Mantel- Haenszel chi-square test or the Mantel-Haenszel chi-square exact test as applicable.	No specific distinction

Sundar-2008a	Frequency and the percentage of patients who achieved the apparent and definitive cure were calculated and compared across the treatment groups using Mantel-Haenszel chi-square test or Mantel-Haenszel chi-square exact test as applicable.	No specific distinction
Sundar-2000c	Cure was achieved in 89% of group A, 100% of group B, and 100% of group C.	No specific distinction
Thakur-2004a	The data for all the patients combined and for those in each treatment arm were checked for normality by calculating the Kolmogorov-Smirnov statistic (KSS) for each variable recorded. For the between-group comparisons, Mann-Whitney U-tests, unpaired Student's t-tests or large-sample tests of difference in proportion were used, as appropriate.	No specific distinction
Das-2009	Comparison of the two treatment groups and their baseline characteristics were done, using large sample test based on Gaussian distribution. Yates's corrected Chi-square test was used for comparing the efficacy and relapse rate of the two regimens. See Table 3.	No specific distinction
Wasunna-2005	All other data were summarized using descriptive statistics only. The efficacy and safety analyses were carried out on the ITT and safety populations, respectively. The ITT population included all patients that had a valid baseline assessment and received at least one dose of study medication. It was subsequently decided to present exact 95% confidence intervals for the primary efficacy outcome calculated using the Clopper Pearson method.	ITT explicitly reported
Thakur-2000a	Cured proportion presented.	No specific distinction
Jha-2005	The efficacy and safety analyses were carried out on the ITT and safety population, respectively. The ITT population was prospectively defined as those patients that had a valid baseline assessment, received at least one dose of study medication and had at least one on-study assessment after Day 21. However, during post hoc analysis, it was thought that this definition may result in censorship of early failures or withdrawals due to adverse events and would not represent a conservative analysis. The ITT population was, therefore, redefined to include all patients that had at least one dose of study medication and was the same as the safety population.	ITT explicitly reported
Sundar-2011a	ITT and per-protocol cure rate presented in Table1 of the manuscript.	Both PP and ITT reported
Sundar-2006	Treatment response rate, were detected by chi-square analysis. Paired-sample t testing was used to compare pre-treatment and posttreatment values in each dose group. The Fleiss quadratic method for proportions was used for the estimation of 95% CIs for the individual proportions. The 3 patients in group B who were lost to follow-up in group B were considered to have treatment failure in the intention-to-treat analysis (table 2).	ITT explicitly reported
Sundar-2004	Chi-square analysis was used to detect differences in sex, previous treatment, and response rates. Paired-sample t testing was used to compare pre- and post-treatment values in each group. The Fleiss quadratic method for proportions was used to compute 95% CIs for the individual proportions of patients who responded to each treatment regimen. $P<0.05$ was considered significant.	No specific distinction

Sundar-2007b	Cured proportion presented in Table 1. Patients who died or were removed from study, defaulted (i.e., left against advice), experienced relapse, or were lost to follow-up at month 6 were considered to have experienced treatment failure. Thus, in the intention-to-treat analysis, overall cure rates, which were not significantly different, were as follows: group A, 96%; group B, 92%; group C, 97%; and group D, 96%.	ITT explicitly reported
Moore-2001	Not a separate statistics section but the following analysis were reported in the manuscript: Fisher's exact test, except * = Mann-Whitney U-test. Chi2 proportions.	No specific distinction
Thakur-2008	Mann-Whitney test, t test and Boxplots method were used to compare the two groups, chi square and Fisher Exact test were used to determine the significance between the outcome of treatment group.	No specific distinction
Sundar-2012	The comparison between the mean values for patients at baseline and day 29 was done by a paired t test and the Wilcoxon signed ranked test. At the end of treatment, the initial cure rate was 97.5% (intention to treat), and 6 months after the end of treatment the final cure rate was 90.3%.	ITT explicitly reported
Thakur-2004b	Differences between proportions was analysed by Chi- square test (unpaired, paired).	No specific distinction
Ritmeijer-2001	For categorical data the x2 test and Fisher's exact test were used.	No specific distinction
Sundar-2000b	Standard binomial 95% CIs for the individual proportions of patients responding to each treatment regimen were computed. Overall, in an ITT analysis, 73 (35%; 95% CI, 28%–42%) of 209 patients from Bihar and 95 (86%; 95% CI, 79%–93%) of 111 subjects from Uttar Pradesh were healthy and relapse free 6 months after treatment and were designated as having definitive cure. On-treatment analysis of subjects who completed 30 days of therapy and who were not lost to follow-up indicated definitive cure in 73 (40%; 95% CI, 33%–47%) of 182 patients from Bihar and in 95 (97%; 95% CI, 94%–99%) of 98 patients from Uttar Pradesh.	On-treatment analysis and ITT
Hailu-2010	Primary and secondary efficacy analyses were by intention to treat (ITT). Where missing parasitological data at the primary endpoint (6-month follow-up) occurred due to loss to follow-up or death unrelated to VL in patients not receiving rescue medication before loss, two analysis approaches were taken within the ITT framework: complete-case analysis, where patients with missing data were excluded, and worst-case analysis, where the missing outcome was assumed to be treatment failure.	ITT explicitly reported
Thakur-2010	Cured proportion; See Table2 of the manuscript.	No specific distinction
Rijal-2013	One hundred twenty patients were started on MIL (intention to treat) for whom a complete 12-month follow-up record was available in all but 1.	ITT explicitly reported
Sundar-2007a	Primary efficacy was calculated as the proportion of patients achieving a final cure; an exact confidence interval for that proportion was computed; the exact, one-sided, upper bound of the 97.5% confidence interval for the difference in success probabilities was compared with the use of $\delta = 0.10$ (the chosen margin for noninferiority).	No specific distinction
Sundar-2002a	Comparison of the trial groups was performed by the Kruskal-Wallis test for ordinal/continuous data (and the chi-square test for nominal data).	No specific distinction
Thakur-2001b	Analysis of variance and chi-squared test was used for statistical analysis of data.	No specific distinction

Musa-2010	Analyses were done on the intention-to-treat (ITT) population. In case of missing data, efficacy analyses were by complete-case analysis, excluding patients with missing data, and by worst-case analysis, where missing efficacy data are assumed to be treatment failures.	ITT explicitly reported
Dietze-2001	Cure proportion presented. See Table 2 of the manuscript.	No specific distinction
Rahman-2011	The per-protocol population was defined as patients who received at least 90% of the planned treatment (i.e., at least 25 days of treatment). The ITT 1 group was defined as patients who received 14–24 days of therapy. The intent-to-treat 2 group was defined as patients who received 1–13 days of therapy. Non-evaluable patients were defined as patients who were lost to follow-up after receiving at least one dose of drug. See Table 1 for the estimates of ITT and PP analysis.	Both PP and ITT reported
Sinha-2011	Of the 494 patients in the intent-to-treat (ITT) population, 98% received a full course of treatment. The overall study completion rate was 94% (462/494) for the ITT population and 96% (461/479) for the efficacy-evaluable (EE) population. Safety analyses were based on the protocol-specified definition of the intent-to-treat (ITT) population, which included all patients who enrolled in the study and received at least one dose of paromomycin. Efficacy analyses were based on the efficacy-evaluable (EE) population, which included all patients without major protocol violations who received at least 20 doses of paromomycin. Table 2 of the manuscript presents estimates for Initial and final clinical cure rates by analysis population	Evaluable population analysis and ITT
Bhattacharya-2007	ITT, intention to treat analysis (including lost patients); PP, per protocol analysis (evaluable patients) are presented in flow diagram. The final cure rate was 82% by intention to treat analysis and 95% by per protocol analysis (similar to the 94% cure rate in hospitalized patients.	Evaluable population analysis and ITT
Mueller-2008	Between-cohort comparisons were made using one-way analyses of variance for the continuous variables, Kruskal–Wallis tests for the discrete variables and x2 or Fisher’s exact tests for the proportions. Results are presented with 95% confidence intervals (CI), where appropriate. See Table 2 for results.	No specific distinction
Khalil-2014	Intention-to-Treat and Per-Protocol complete-case analysis populations were identical at day 30 & day 210. Cumulative data for each treatment regimen were used to calculate the percentage of patients cured, with exact binomial 95% confidence intervals (CI), at day 30- and 6-months follow-up in ITT and per-protocol (PP) analysis populations. Patients with missing outcome data were excluded from analyses.	Both PP and ITT reported
Sundar-2009b	Definitive cure at 6 months of follow up was seen in 82% of patients in group A and 92% of patients in group B by intention-to-treat analysis and in 84.3% of patients in group A and 92.8% of patients in group B by per protocol analysis. See Table 2 of the manuscript.	Both PP and ITT reported
Sundar-2000a	Exact binomial 95% confidence intervals (95% CI) for the individual proportions responding at day 30 and 6 months were computed. "Although this patient showed no evidence of relapse at 3 months, he is included as a treatment failure in this intention-to-treat analysis."	ITT explicitly reported
Sundar-2003b	The intent-to-treat population included all patients who received the trial medication.	ITT mentioned

Sundar-2010	The intention-to-treat analysis included all subjects who received at least one dose of a study drug. The per-protocol analysis included all patients who completed treatment and all protocol requirements. See Table 2.	Both PP and ITT reported
Musa-2012	Efficacy data were analysed according to Intention-to-Treat (ITT) and Per-Protocol (PP). The PP population excluded those with pre-specified major protocol deviations (i.e. consent withdrawal after taking a dose of study medication, receipt of under 70% or over 130% of the expected treatment dosage, or receipt of alternative treatment to that of random allocation). See Table 2 Efficacy is measured as the percentage of patients cured per arm.	Both PP and ITT reported
Sundar-2001	Compared response rates in the treatment groups by Fisher's exact test and calculated exact binomial 95% confidence intervals for the individual proportions responding to each treatment regimen. The objective of this study was to gather preliminary data on the relative efficacy of two treatments that would, if the results warranted it, inform the design of a more precise trial with appropriate statistical power.	No specific distinction
Thakur-2000b	Cured proportion presented in Table 1.	No specific distinction
Mondal-2010	Cured proportion presented. No lost to follow-up occurred.	No specific distinction
Veeken-2000	For parametric data, Z score test for comparison of means was used; for nonparametric data, the Mann Whitney test; for categorical data, the x2 test for trend.	No specific distinction
Rijal-2010	For the analysis of risk factors for failure of SSG, a per-protocol perspective was adopted, i.e. only kala-azar patients who received ≥25 doses of SSG were included in the analysis. Those who died before Day 25 while being treated with SSG were excluded from the analysis as their death was most likely related to the disease severity with complications such as bleeding or septicaemia. Also, those for whom SSG therapy was switched to the second-line drug amphotericin B owing to side effects were excluded, as were cases that could not be followed up for 12 months after therapy. From an intention-to-treat perspective, SSG cure rates were much lower (77.3%).	Both PP and ITT reported
Patra-2012	Cured proportion presented.	No specific distinction
Figueras Nadal-2003	Cured proportion presented.	No specific distinction
Singh-2010	Cured proportion presented.	No specific distinction
Bhattacharya-2004	Cured proportion presented; The final cure rate was 94% for all enrolled patients and 95% for evaluable patients.	No specific distinction
Singh-2006	Cured proportion presented.	No specific distinction
Singh-2006	The final cure rates on per protocol basis, in which all patients who could be followed for trial period analysed, were 93.2%, 95%, 92.10% and 91.30% in-group 1, 2, 3 and 4 respectively. Intent to treat analysis reported in Table 2.	Both PP and ITT reported
Sundar-2003	Analysis was done by ITT and PP approach; See Table 2 for the estimates.	Both PP and ITT reported
Syriopoulou-2003	Number of cured patients reported along with denominator.	No specific distinction

Haidar-2001	Cured proportion presented.	No specific distinction
Das-2005	Cured proportion presented.	No specific distinction
Rijal-2003	Cured proportion presented.	No specific distinction
Das-2001	Cured proportion presented.	No specific distinction
Ostyn-2014	Calculated the cure and failure rates at the end of treatment and at 6- and 12-months post-treatment in an intent-to-treat (ITT) and per protocol (PP) perspective (definitions for ITT and PP analysis are provided in Table S1). For the per-protocol analysis, only patients with a complete 28 days treatment were considered. For the 6- and 12-months post-treatment outcomes, we included only those patients with a complete follow-up in the PP analysis, while in ITT, all lost-to-follow ups were considered as failures.	Both PP and ITT reported
Sinha-2010	Treatment success rates were calculated according to intention- to-treat (ITT), per protocol, and worst-case ITT scenario analyses; these approaches were used to estimate treatment efficacy in human African trypanosomiasis trials. An ITT analysis considered relapses and all deaths as failures; successes included lost to follow-up patients considered cured at least one time without a relapse. A per protocol analysis considered only relapses and deaths clinically attributed to kala-azar as failures. A worst-case ITT scenario analysis considered #all relapses, deaths, and lost to follow-up patients as failures.	Both PP and ITT reported
Mueller-2007	Cured proportion.	No specific distinction
Cota-2014	Cured proportion.	No specific distinction
Sudarshan-2011	Parasite clearance.	No specific distinction
Adam-2009	Proportion presented.	No specific distinction
Shahian-2009	No relapse occurred.	No specific distinction
Villanueva-2000	The Kruskal-Wallis test was employed for the statistical analysis of continuous variables. Qualitative variables were compared using the chi-square test with the Mantel-Haenszel correction or Fisher's exact test. "Relapses were observed in 5 of 20 patients. These results indicate that HAART neither prevents the incidence of VL relapse nor modifies the clinical picture described in the pre-HAART era."	No specific distinction
Goswami-2016	For safety, the number and percentage of patients experiencing toxicities and AEs (including laboratory abnormalities) across two treatment groups were recorded as per protocol and was presented in a tabular form. No specific distinction made for efficacy estimation.	No specific distinction
Jamil-2015	One hundred fifty-three children and adults were screened, of which 120 patients were enrolled and included in the intent-to-treat (ITT) population (Fig 1). A total of 117 subjects completed the study as per protocol (1 subject withdrew consent before receiving study drug and was excluded from the Safety population; 1 subject had a serious adverse event [SAE], and 1 subject died [described below in Safety Results]).	Both PP and ITT reported

Sundar-2015	Data were analysed using an intent-to-treat (ITT) population.<br>Remark: Data was complete with only 1 relapse in each arm of the 15 patients with no further deviations or lost to follow-up. Hence PP and ITT will coincide in this study.	ITT explicitly reported
Sundar-2014	The efficacy analysis was performed on modified intent-to-treat (mITT) population, which includes all patients who received study drug as per the protocol specified duration and had at least one efficacy assessment throughout the study. Safety analysis was performed on intent-to-treat (ITT) population, which includes all patients who received the treatment of study drug.	mITT explicitly reported
Mondal-2014	Estimated efficacy in terms of initial cure (at day 30) and final cure (at 6 months), and safety in all patients who were enrolled (intention-to-treat analysis). Also assessed efficacy in all patients who completed treatment and 6-month follow-up after treatment with or without visceral leishmaniasis relapse (per protocol analysis).	Both PP and ITT reported
Sundar-2019	At day 30, the initial cure rate was 100%; however, at 6 months and 12 months, cure rates were 97.0% and 94.2% by per-protocol analysis and 96.9% and 93.9% by intension-to-treat analysis, respectively.	Both PP and ITT reported
Diro-2019	The intention to treat (ITT) population was pre-specified as primary analysis population for the sequential interim analyses of treatment success at D29. Both ITT and per-protocol (PP) populations for treatment success at D29 and D58 were used for final analyses of treatment success at D29 and D58. ITT was considered primary analysis population.	Both PP and ITT reported
Mbui-2018	The primary population for efficacy analysis at days 28 and 210 was the intention- to-treat population (ITT). The per-protocol (PP) population included patients with no pre-specified major protocol deviations relating to treatment compliance and baseline exclusion criteria. The trial was completed without any loss to follow-up. The ITT and PP populations were of identical sizes (n = 30).	Both PP and ITT reported
Borges-2017	The analysis of primary outcome was planned on an intention-to-treat basis (ITT) considering all subjects as originally assigned to the two arms. Patients who needed therapy change due to adverse effects and those lost during treatment or follow-up were considered as treatment failures. No subgroup analysis was initially planned. According to per protocol analysis, all (except one child from ABD group) patients who completed treatment were cured. In this analysis, 48 children treated with MA and 48 treated with ABD were included.	Both PP and ITT reported
Wasunna-2016	The primary analysis was by intention- to-treat (ITT). 147 patients were included in the per-protocol (PP) population, 50 for the AmBisome + SSG arm, 48 for the AmBisome + miltefosine arm and 49 for the miltefosine monotherapy arm. Results from the per protocol analysis were similar to the ITT population.	Both PP and ITT reported
Rahman-2017	The Intention to Treat population (ITT) included all patients randomised to the treatment groups who gave informed consent and who took at least one dose of study medication. The Per Protocol population (PP) included all patients in the ITT population with no major protocol deviations and who completed the 6-month follow-up visit or were classified as a treatment failure and received rescue medication.	Both PP and ITT reported

Romero-2017	The primary efficacy endpoint of cure at 6 months was analysed as to the intention-to-treat (ITT) and per-protocol (PP) approaches. The ITT analysis included all participants randomized to the remaining three interventions, except for one participant randomized to the MA arm and withdrawn from the trial at D1 after a stool tested positive for schistosomiasis. Therefore, the ITT analysis included a total of 332 participants. The PP analysis excluded participants lost to follow-up and withdrawn from the trial because of the occurrence of AE/SAE.	Both PP and ITT reported
Goswami-2020	The primary end point of definitive cure rate was analysed for all randomly assigned patients (intention to treat [ITT]) and for the per-protocol patients. The per-protocol group consisted of all patients who were enrolled, had no major protocol deviation or serious adverse effect mandating complete withdrawal of drug therapy, received the full treatment, and were assessed both at baseline and at the end of treatment.	Both PP and ITT reported
Alborzi-2017	All principal analyses were performed in the intention to-treat population, which consisted of all the patients who underwent randomization, regardless of the treatment received. The patients who were lost to follow-up and in whom no known event had occurred were not included in the denominator for calculation of binary end points.	ITT explicitly reported
Pandey-2017	Definitive cure rate at 6 months by per protocol analysis was 97.9% [95% confidence interval: 92.7–99.4].	PP explicitly reported
Pandey-2016	Text and Figure 1; The initial and final cure rate was 97.4% and 85.6%, respectively.	No specific distinction
Kimutai-2017	The analysis population was the intention-to-treat (ITT) population, meaning that any patient who received at least one dose of SSG-PM combination treatment was included in the analysis population.	ITT explicitly reported
Goyal-2018	Two effectiveness analyses were performed. In the intention-to-treat (ITT) analysis, all patients who received at least one drug dose were included; those with treatment stopped, treatment default, or lost to follow-up at 6 months were considered as treatment failures. In the complete case analysis, those with treatment stopped, default, or lost to follow-up at 6 months were excluded.	Complete case analysis and ITT
Sinha-2019	Cured proportion presented.	No specific distinction
Ekram-2021	Cured proportion presented.	No specific distinction

ITT = Intention-to-treat; PP = Per-protocol; CC = Complete case analysis; mITT = Modified ITT.
